# Precision peptide theranostics: developing *N*- to *C*-terminus optimized theranostics targeting cholecystokinin-2 receptor

**DOI:** 10.7150/thno.89701

**Published:** 2024-02-24

**Authors:** Marwa N. Rahimi, Alicia Corlett, Jessica Van Zuylekom, Marc Antoine Sani, Benjamin Blyth, Philip Thompson, Peter D. Roselt, Mohammad B. Haskali

**Affiliations:** 1 Department of Radiopharmaceutical Sciences, Cancer Imaging, The Peter MacCallum Cancer Centre, Victoria 3000, Australia.; 2 Sir Peter MacCallum Department of Oncology, The University of Melbourne, Victoria 3010, Australia.; 3 Cancer Imaging, Peter MacCallum Cancer Centre, Melbourne, Victoria 3000, Australia.; 4 Models of Cancer Translational Research Centre, The Peter MacCallum Cancer Centre, Victoria 3000, Australia.; 5 The Bio21 Institute, School of Chemistry, The University of Melbourne, Melbourne, Victoria, 3010 Australia.; 6 Medicinal Chemistry, Monash Institute of Pharmaceutical Sciences, Faculty of Pharmacy and Pharmaceutical Sciences, Monash University (Parkville Campus), Parkville, Victoria 3052, Australia.

## Abstract

Peptides are ideal for theranostic development as they afford rapid target accumulation, fast clearance from background tissue, and exhibit good tissue penetration. Previously, we developed a novel series of peptides that presented discreet folding propensity leading to an optimal candidate [^68^Ga]Ga-DOTA-**GA1** ([D-Glu]_6_-Ala-Tyr-*N*MeGly-Trp-*N*MeNle-Asp-Nal-NH_2_) with 50 pM binding affinity against cholecystokinin-2 receptors (CCK_2_R). However, we were confronted with challenges of unfavorably high renal uptake.

**Methods:** A structure activity relationship study was undertaken of the lead theranostic candidate. Prudent structural modifications were made to the peptide scaffold to evaluate the contributions of specific *N*-terminal residues to the overall biological activity. Optimal candidates were then evaluated in nude mice bearing transfected A431-CCK_2_ tumors, and their biodistribution was quantitated *ex vivo*.

**Results:** We identified and confirmed that D-Glu^3^ to D-Ala^3^ substitution produced 2 optimal candidates, [^68^Ga]Ga-DOTA-**GA12** and [^68^Ga]Ga-DOTA-**GA13**. These radiopeptides presented with high target/background ratios, enhanced tumor retention, excellent metabolic stability in plasma and mice organ homogenates, and a 4-fold reduction in renal uptake, significantly outperforming their non-alanine counterparts.

**Conclusions:** Our study identified novel radiopharmaceutical candidates that target the CCK_2_R. Their high tumor uptake and reduced renal accumulation warrant clinical translation.

## Introduction

Over the last 60 years, advances in molecular and cell biology techniques have transformed the foundations of our modern medical scientific understanding. This has allowed researchers and clinicians to work concertedly to unravel the cellular pathways that drive cancer and develop new techniques for non-invasive, high-resolution, *in vivo* imaging technologies 1-3. Targeted molecular imaging is an indispensable research and clinical tool that provides diagnostic, prognostic, and predictive information in oncology as well as cardiology, neurology, and infectious and inflammatory disorders 4. Unlike anatomical imaging techniques such as computed tomography (CT) and magnetic resonance imaging (MRI), molecular imaging techniques such as positron emission tomography (PET) examine the cellular abnormalities that are the basis of malignancies, quantifying target expression using imaging probes 5, 6. The evolution of PET and single photon emission computed tomography (SPECT) imaging methods has afforded new opportunities to produce exceptional streamlined diagnostic and therapeutic radiopharmaceuticals using the same targeting vector 4, 7. By combining therapeutics and diagnostics using the same vector but different radionuclides, we can produce theranostic radiopharmaceuticals 8.

In recent years, work in theranostic radiopharmaceuticals has seen unprecedented acceleration with various radioisotope-labeled ligands being introduced for the diagnosis and therapy of metastatic neuroendocrine tumors, prostate cancer and many other malignancies 9, 10. These successes have inspired the development of many other peptide-based radiopharmaceuticals that deliver systemic treatment designed to eliminate both residual primary lesions and metastatic cancer cell deposits 11, 12.

The cholecystokinin-2 receptor (CCK_2_R) is a highly promising target in nuclear medicine and has been the focus of radiopharmaceutical development over the past two decades 13. CCK_2_R is overexpressed in multiple tumor states, including medullary thyroid carcinomas 14, small cell lung cancer 14, somatostatin-2 negative neuroendocrine tumors 15, stromal ovarian cancer 16, 17 and gastrointestinal stromal tumors 18, 19. Additionally, targeting CCK_2_R has shown promising results for gastroenteropancreatic and bronchopulmonary neuroendocrine tumors, especially insulinomas and vipomas, as well as bronchial and ileal carcinoids 13, 18. The absence of CCK_2_R expression in normal, healthy surrounding tissues and overexpression in the tumor environment provides favorable opportunities for high-contrast diagnostic imaging and radionuclide therapy of these tumors, making CCK_2_R an excellent target for the development of radiolabeled peptides for diagnosis and therapy in nuclear medicine.

**Figure 1:**
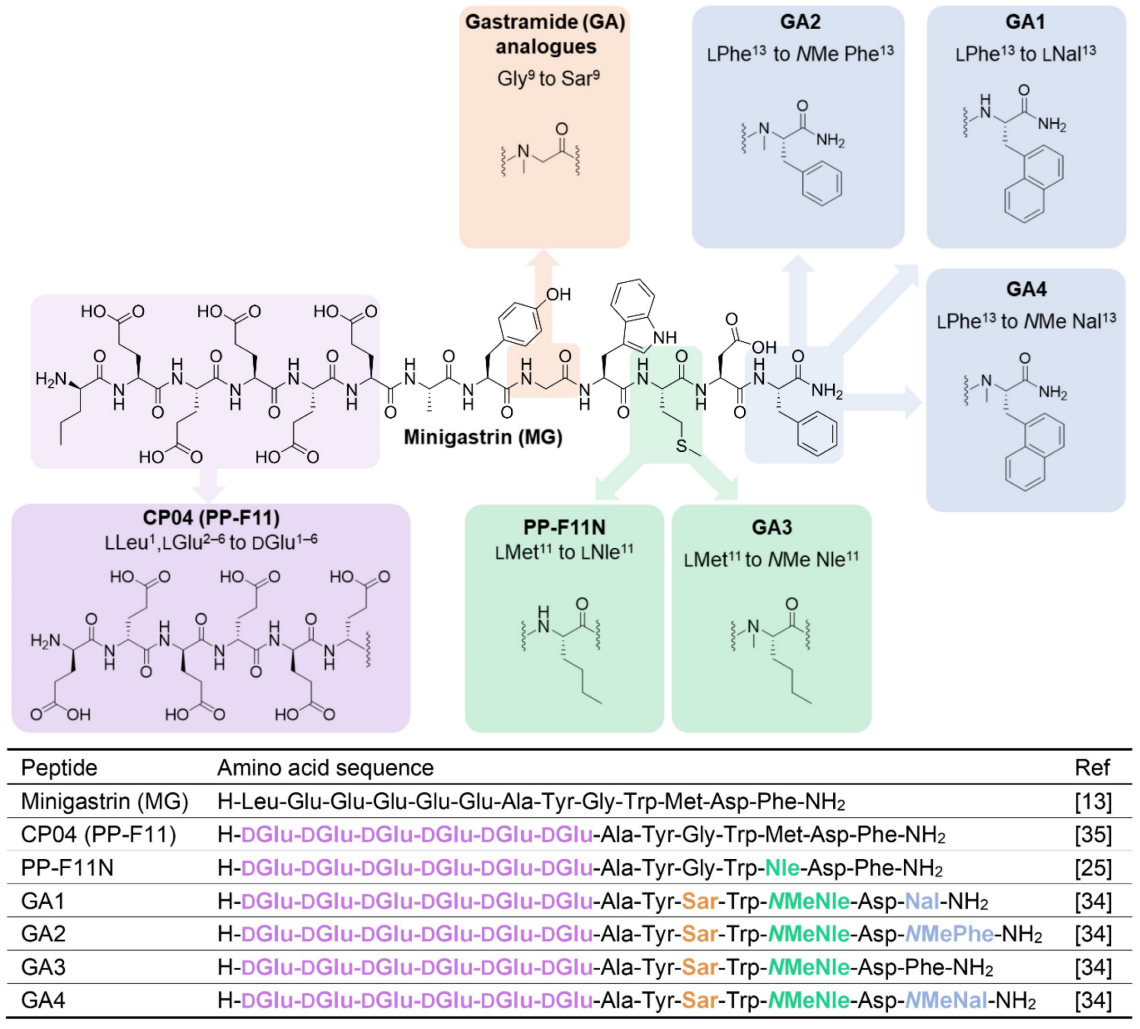
Chemical structures (above) and amino acid sequences (below) of CCK_2_R binding peptides. Minigastrin (**MG**), the lead molecule, and structural modifications made to produce high affinity binding Gastramide (**GA**) peptides.

Gastrin (**G17**), the natural ligand of CCK_2_R, and minigastrin (**MG**) (Figure 1), the truncated variant of the endogenous molecule, have undergone several stages of development by various groups to identify critical binding features, including truncating the lengthy peptide and optimizing several other characteristics to make it suitable for use as a theranostic radiopharmaceutical in nuclear medicine. The pentameric glutamic acid chain located on the *N*-terminus of the **MG** peptide contributes to the increased biological potency and higher binding affinity by promoting the peptide to fold in a hairpin structure, thereby stabilising the peptide in a biologically active conformation 20-22. The *C*-terminus of the **MG** peptide houses the critical tetrapeptide sequence [Trp-Met-Asp-Phe-NH_2_], responsible for the recognition of the peptide by the CCK_2_R target 23. Many modifications have been explored in these regions, seeking to address issues presented with the overall metabolic stability and biodistribution of the peptide. These endeavors have produced the clinically translated minigastrin analogs **CP04** (**PP-F11**) and **PP-F11N** 24, 25. Substitution of the penta-L-glutamic acid chain to its D-glutamic acid (D-Glu) and the addition of a sixth D-Glu residue in both **CP04** and **PP-F11N** resulted in enhanced metabolic stability to the parent **MG** peptide, a known technique to safeguard peptides against enzymatic attack in the cellular environment.

Renal reabsorption of radiolabeled peptides is a clinical problem, often restricting the maximum therapeutic dose of the theranostic, with the kidneys being the dose-limiting organ in peptide receptor radionuclide therapy (PRRT) 26, 27. There have been several strategies explored by groups to address this key issue of renal accumulation of CCK_2_R targeting peptides. It is established that high kidney retention of the **MG** analogs is related to the presence of *N*-terminal ionic glutamic acid residues 28. Therefore, a popular approach to suppress unfavorable kidney uptake has been the truncation of the implicated pentameric glutamic acid chain in the **CP04** sequence to produce **MG11** 21, 29-32.

Roques and coworkers were early explorers of the CCK_2_R landscape. They conducted a comprehensive investigation of promising CCK_2_R ligands, primarily on the CCK_8_ and CCK_4_ scaffold 33. They substituted *N*-methylated norleucine (*N*Me-Nle) for methionine (Met) and introduced bulky aromatic naphthylalanine (Nal) to the scaffold, which vastly improved affinity, selectivity, and bioavailability of peptides to CCK_2_R 33. These modifications were incorporated in **MG11** to produce the high-affinity ligand **MGS5** 32. Its truncated length due to deletion of the *N*-terminal glutamic acid residues and *N*-methylation of the *C*-terminus led to reduced kidney accumulation by a factor greater than 25, resulting in significant improvement of the tumor-to-kidney ratio 29.

Recent work by our group dramatically improved upon the biological and pharmacological properties of **CP04** by employing the modifications explored by Roques and coworkers concurrently and independently of **MGS5** developments. By incorporating conformational constraints such as *N*-methylation of several residues and enhancing the amphiphilic character of the peptides, we developed a novel class of optimized peptides which we denoted as Gastramide (**GA**). **GA** peptide analogs afforded low pM affinity against CCK_2_R (Figure 1) and confirmed the important contributions of secondary peptide conformations in enhancing the biological affinity of peptides to their target 34. This investigation resulted in the first generation of optimized candidates **GA1**, **GA3**, and **GA4** (Figure 1) 34. Despite these astounding improvements to the overall stability and affinity of the peptides, reducing their uptake and retention in the kidneys would further increase their suitability for clinical theranostic applications 26.

In the present work, our structural investigation of the **GA** scaffold resumes with a comprehensive evaluation of the established **GA1**. Previously shown to have low picomolar affinity (IC_50_ 50 pM for CCK_2_R), **GA1** is an ideal candidate for conducting a structure activity relationship (SAR) study to determine the importance of individual *N*-terminal glutamic acid (Glu) residues in contributing to target affinity. We aim to improve pharmacokinetic properties and reduce renal uptake of the peptide by identifying nonessential residues and substituting them with more favorable alternatives, to produce optimal theranostic candidates.

## Methods, Results, and Discussion

**Figure 2:**
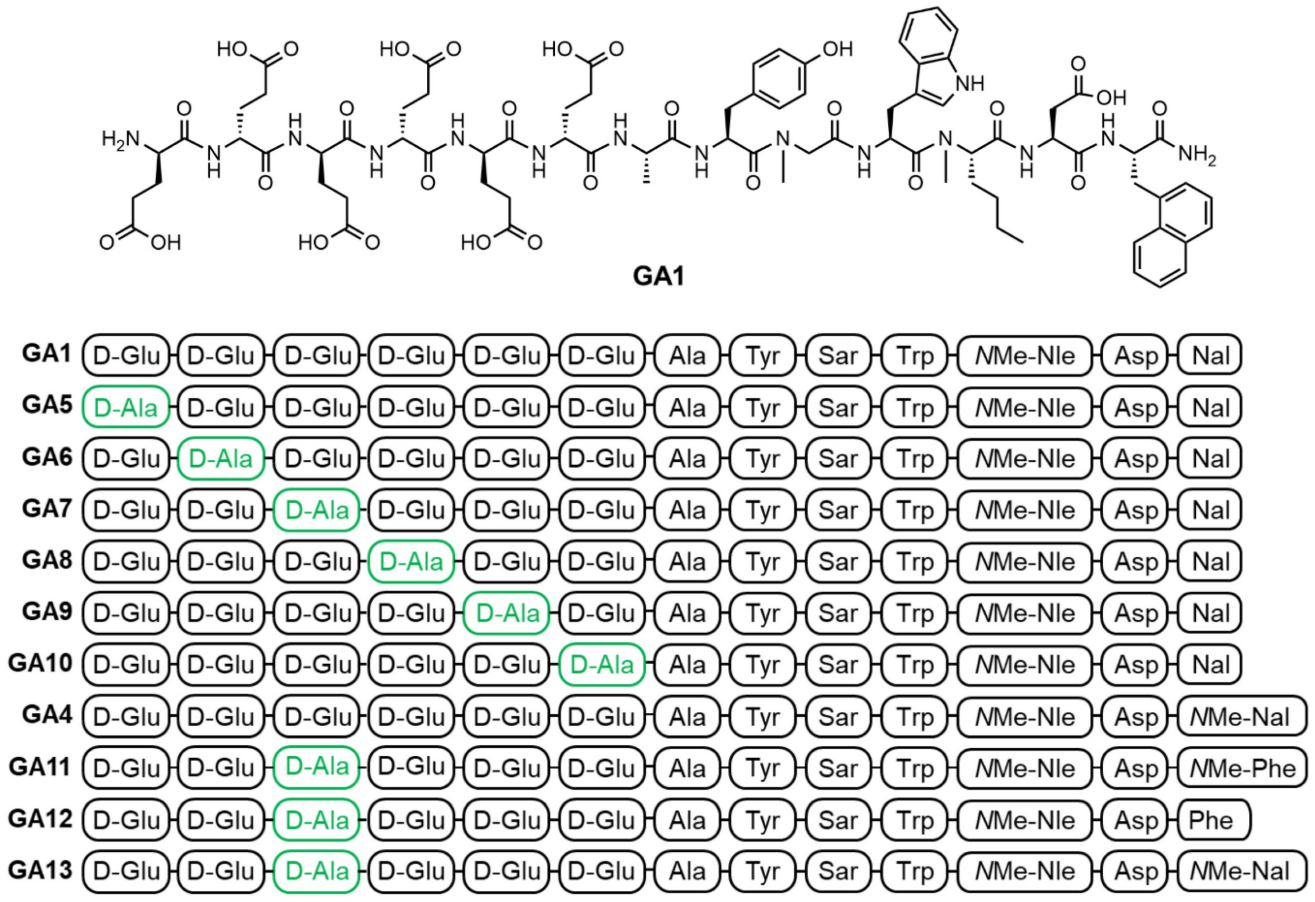
The chemical structure of linear peptides **GA1** (top) and peptide sequences of alanine analogs **GA1**, **GA4-GA13** (bottom). Residues highlighted in green indicate positions of D-Ala substitution.

*** Peptide synthesis, DOTA coupling and ^nat^Ga/^68^Ga-labeling:*** We prepared a D-Ala scan library (**GA5-GA10**) based on **GA1** by systematically substituting each one of the *N*-terminal D-Glu residues (Figure 2). This enabled the evaluation of the synergistic effect of each D-Glu on the peptides' overall potency. Peptides tolerating D-Ala substitution were then modified at the *C*-terminal residue (**GA4, GA11-13**) to prepare metabolically stable and potent theranostic candidates (Figure 2).

**Figure 3:**
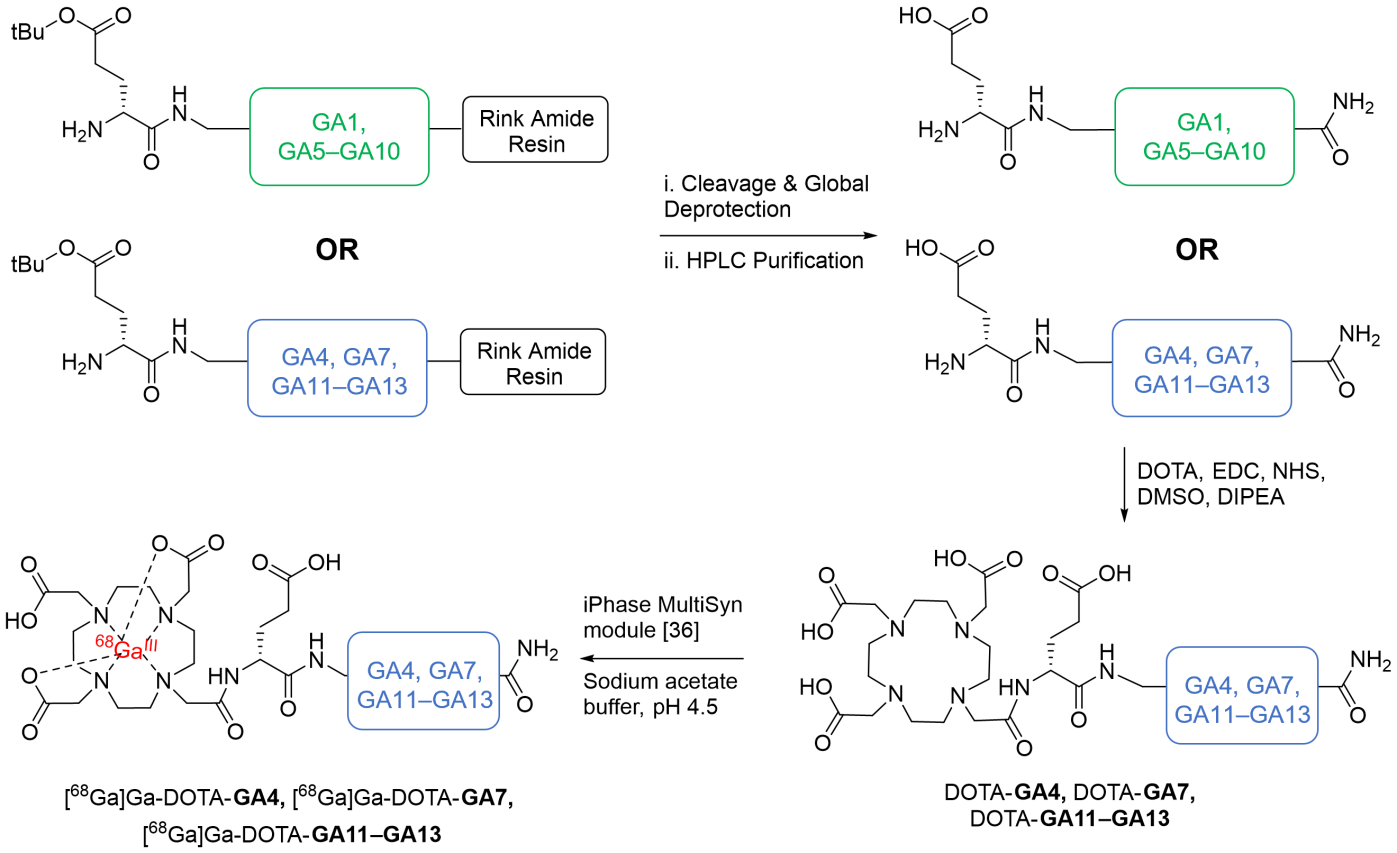
The chemical synthesis of linear peptides **GA1**, alanine analogs **GA5-GA10** (in green), and **GA4, GA7, GA11-13** (in blue). Peptides **GA4, GA7, GA11-13** were carried forward for DOTA conjugation and subsequent radiolabeling, yielding DOTA-**GA4**, DOTA-**GA7**, DOTA-**GA11-GA13** and their ^68^Ga labeled peptides.

The established minigastrin analogue **CP04** (PP-F11) (Figure 1), its derivative **GA1,** D-Ala substituted **GA5-GA10**, and other CCK_2_R scaffolds **GA4, GA11-13** were synthesized following the method described in our supplementary data and afforded moderate yields using standard DIC/Oxyma based Fmoc solid phase peptide synthesis conditions (Figure 3) 34. The highly acidic *N*-terminus of the peptide led to frequent solubility issues in aqueous compositions. All peptides synthesized were dissolved in an acetonitrile:water mixture and purified immediately after trituration with ethyl ether to minimize undesirable aggregation. All linear peptides were synthesized, isolated, and lyophilized to afford an amorphous white powder with a >20 % yield and >94% purity (Table 1).

The synthesis and purification of **GA5**, **GA6** and **GA10** encountered specific challenges. Substitution of the alanine at positions 1, 2 and 6 on the parent **GA1** scaffold, reducing the acidic nature at the *N*-terminus of the peptide, led to increased product aggregation and lower overall yield post-purification (Table 1). In comparison, the substitution of alanine at the remaining positions did not face similar aggregation issues.

Another synthetic challenge common to all peptides discussed in this work was the deletion of glutamic acid residues, which led to the formation of side products with deletion sequences. During on-resin solid phase peptide synthesis, amino acid couplings were confirmed *via* small-scale cleavage and deprotection, and the resulting product mass was confirmed using mass spectrometry. All linear peptides generated incomplete coupling of D-Glu residues at positions 3 and 4, with deletion products being observed during initial HPLC purification. Amongst the structures discussed in this work, deletion product peptides constituted 5-30% of the final isolated peptide, and their structural and chemical similarity to the full-length peptide led to difficulties separating the products during HPLC purification. The challenge of glutamic acid deletions at positions 3 and 4 was addressed by increasing the coupling times of these residues under microwave conditions and performing double couplings of the glutamic acid residues. These specific modifications eliminated the deletion products altogether, leading to the yields reported in Table 1.

### Competition binding studies

Once all *N*-terminal free peptides were in hand, **CP04**, the parent **GA1,** and its D-Ala substituted analogs **GA5**-**GA10** were evaluated for their ability to disrupt the binding between CCK_2_R and [^177^Lu]Lu-DOTA-**CP04** *in vitro,* using the epidermoid carcinoma cell line A431 transfected to stably express CCK_2_R (A431-CCK_2_R) 35, 36. [^177^Lu]Lu-DOTA-**CP04** was used as the competitive radioligand to determine the CCK_2_R binding affinity of the linear peptides as it has an established high binding affinity to the receptor of interest and thus serves as an ideal positive control in these assays 34.

**Figure 4:**
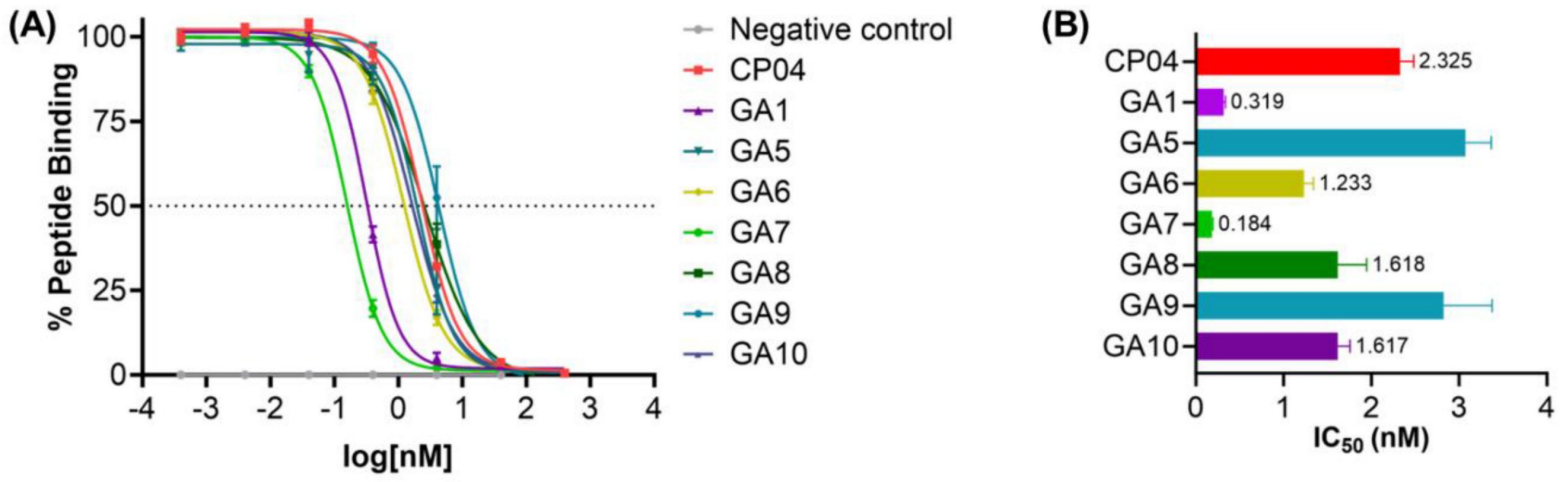
** (A)** Binding curves for IC_50_ determination of **CP04**, **GA1** and D-Ala scan peptides **GA5-GA10** against [^177^Lu]Lu-DOTA-**CP04** in A431-CCK_2_R cells. Curves are representative of 2-4 biological replicates performed in three technical replicates (n=3). **(B)** Binding affinities (IC_50_) for the human CCK_2_R, expressed as an average across 2-4 biological replicates performed in three technical replicates.

The apparent binding affinities (IC_50_) (Figure 4) demonstrate the impact of D-Ala substitution on each of the glutamic acid residues on the critical hexameric peptide sequence. Linear **CP04** without gallium coordinated DOTA at the *N*-terminus showed a greater than 3-fold decrease in affinity for the CCK_2_R target, from 0.76 nM to 2.33 nM. This loss of affinity very clearly demonstrates the importance of the radiometal-DOTA complex in improving the affinity of the peptide to the receptor of interest. Similarly, **GA1** had previously been assessed in its ^nat^Ga-DOTA-**GA1** form with an affinity of 0.05 nM for CCK_2_R but observed a 5-fold reduction in its affinity to the receptor without the gallium-coordinated DOTA at the *N*-terminus 34. Despite this, **GA1** with a free *N*-terminus established an important baseline for the subsequent peptides, with an affinity of 0.32 nM.

**GA5**, with D-Ala substituted at terminal position 1, saw a significant loss of binding affinity to CCK_2_R (Figure 4). This clearly demonstrates the important role of the *N*-terminal D-Glu residue in improving the binding affinity of the peptide sequence to the target, as observed by the greater than 10-fold loss of activity. Similarly, **GA9** observed an equivalent loss of activity for the receptor. Evaluating the biological activity of substitutions made at positions 2, 4, and 6 in peptides **GA6**, **GA8**, and **GA10,** respectively, found that the removal of the D-Glu moiety was also poorly tolerated. The resulting activity across these peptides exhibited an approximately 5-fold loss in binding affinity to the target receptor across all three peptides (Figure 4).

**GA7**, with the substitution of D-Ala at position 3, yielded the most active ligand of the library of peptides evaluated. With an IC_50_ of 0.18 nM, **GA7** displayed a greater than 10-fold improvement upon the activity of the established **CP04**. It also exceeded the already outstanding affinity of its predecessor **GA1** by almost 2-fold. Position 3 therefore, is not only highly tolerable of modifications, but the substitution of a neutral hydrophobic moiety such as D-Ala greatly improves the binding between the ligand and receptor. Considering the 3-dimensional β-hairpin structure of this scaffold, it is possible that the highly polar and bulky D-Glu at this position repels favorable intramolecular interactions and may also negatively influence the binding in CCK_2_R. Furthermore, alanine residues are helix-stabilising in peptides and may infer conformational stability when substituted in position 3 of **GA7** 37.

The D-Ala substitutions at positions 1 and 5 may suggest at the vital nature of the D-Glu residues in the formation and stability of a secondary structure; the loss of these critical residues results in a loss of activity. Previous work in this scaffold has shown that the linear peptide preferentially folds into a β-hairpin structure through backbone and residue intramolecular interactions and that this secondary structure is critical for its exquisite activity 34. Furthermore, Moroder *et al.*, and Peggion *et al.*, demonstrated that the *N*-terminus of CCK and Gastrin peptides adopt α-helical orientation, which induces ordered conformations of the *C*-terminus involving a β-turn 38-41. Interestingly, Peggion and coworkers also demonstrated that Glu-residues at positions 1 and 2 of des-Trp^1^,Nle^2^-**MG** form hydrogen bonds to Glu residues 5 and 6, to form the anticipated α-helical structure of *N*-terminus 40. Recently, cryo-electron microscopy confirmed that the 17 amino acid long hormone gastrin-17 indeed forms a β-hairpin structure upon binding to CCK_2_R with the five *C*-terminal residues inserting deeply into the binding pocket of CCK_2_R 42. Furthermore, the *N*-terminal section of gastrin-17 also orientates into a shallow binding cavity of CCK_2_R 42.

The *C*-terminal residue of gastrin-17 based peptides is prone to metabolic degradation with *N*-methylation of this position, leading to a metabolically stable analogue named **GA4** (Figure 2). This peptide presented advantageous biostability resulting in the highest tumor uptake in biodistribution mouse studies when evaluated alongside other potential ligands 34. However, **GA4** also had the highest renal uptake of compounds tested 34. This is in large part due to the highly acidic hexameric terminal peptide chain, known to promote kidney uptake 29, 43. **GA4** serves as an important lead scaffold to investigate the impact of D-Ala substitution, reducing the overall acidic nature of the peptide and screening the impact of this structural change on the peptide binding affinity of the peptide to CCK_2_R target. **GA7, GA11-GA13** (Figure 2) were designed to implement the favorable D-Ala substitution at position 3 alongside changes to the critical *C*-terminal residue. By systematically incorporating bulky aromatic naphthylalanines and *N*-methyl amino acids, we intended to enhance the peptide's structural conformity, increase affinity, and improve metabolic stability.

DOTA coupled peptides DOTA-**GA4**, DOTA-**GA7,** DOTA-**GA11-GA13** were then coordinated to natural gallium (^nat^Ga), producing ^nat^Ga**-**DOTA-**GA4,** ^nat^Ga**-**DOTA-**GA7**, ^nat^Ga**-**DOTA-**GA11**-**GA13,** respectively. Previous work from our laboratory had demonstrated that a DOTA conjugation in solution was a far more effective strategy to produce peptides in good yields compared to the traditional on-resin coupling of DOTA-tris (*tert*-butyl ester) 34. As such, **GA4, GA7**,** GA11**-**GA13** were conjugated to DOTA in solution (Figure 3) to obtain DOTA-peptides DOTA-**GA4,** DOTA-**GA7,** DOTA-**GA11**-**GA13** at yields of 64-86% from the respective linear peptides (Table S2).

Natural gallium was then coordinated to peptides DOTA-**GA4,** DOTA-**GA7,** DOTA-**GA11**-**GA13** in a sodium ascorbate-buffered solution (pH 4.5) to generate the non-radioactive reference peptides ^nat^Ga**-**DOTA-**GA4,** ^nat^Ga**-**DOTA-**GA7**, ^nat^Ga**-**DOTA-**GA11**-**GA13**. ^nat^Ga-DOTA-peptides were then purified using preparative HPLC to remove salts and afford high-purity products for ligand binding assays (Table S3).

**Figure 5:**
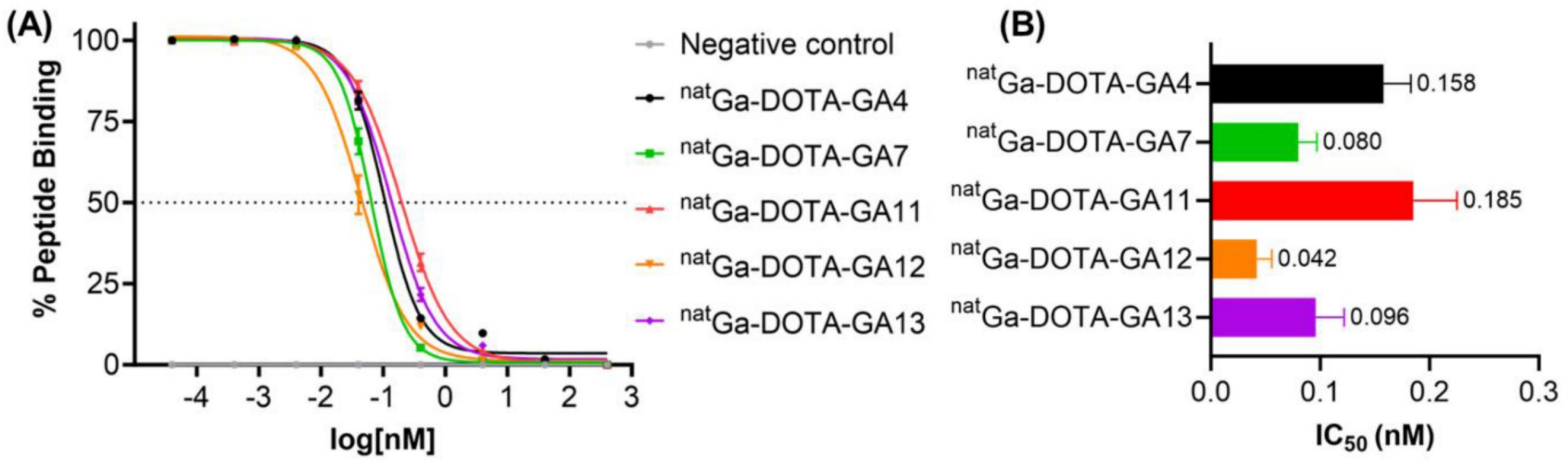
** (A)** Binding curves for IC_50_ determination of peptides ^nat^Ga-DOTA-**GA4**, ^nat^Ga-DOTA-**GA7** and ^nat^Ga-DOTA-**GA11-GA13** against [^177^Lu]Lu-DOTA-**CP04** in A431-CCK_2_R cells. Curves are representative of 2-4 biological replicates performed in three technical replicates (n=3). **(B)** Binding affinities (IC_50_) for the human CCK_2_R, expressed as an average across 2-4 biological replicates performed in three technical replicates

Modification of the *N*-terminus of all **CP04**-based peptides with ^nat^Ga-DOTA had previously been determined to improve binding affinity of the peptide to CCK_2_R 34. Peptides **GA1**, **GA3**, and **GA4** have previously been investigated and shown to have a sub-nanomolar affinity to the CCK_2_R target 34. To determine the impact of D-Ala substitution at position 3, ^nat^Ga-DOTA peptides were assayed against [^177^Lu]Lu-DOTA-**CP04** and shown to tolerate the substitution extremely well, with less than 2-fold loss of binding affinity across all peptides tested with varying substitutions at *C*-terminus (Figure 5). Affinity assays demonstrated conclusively that substitution of D-Ala for D-Glu at position 3 on this scaffold has little to no negative impact on the affinity of the peptides to the target receptor while minimizing overall negative ionic charge.

### Circular Dichroism (CD) Spectroscopy

**Figure 6:**
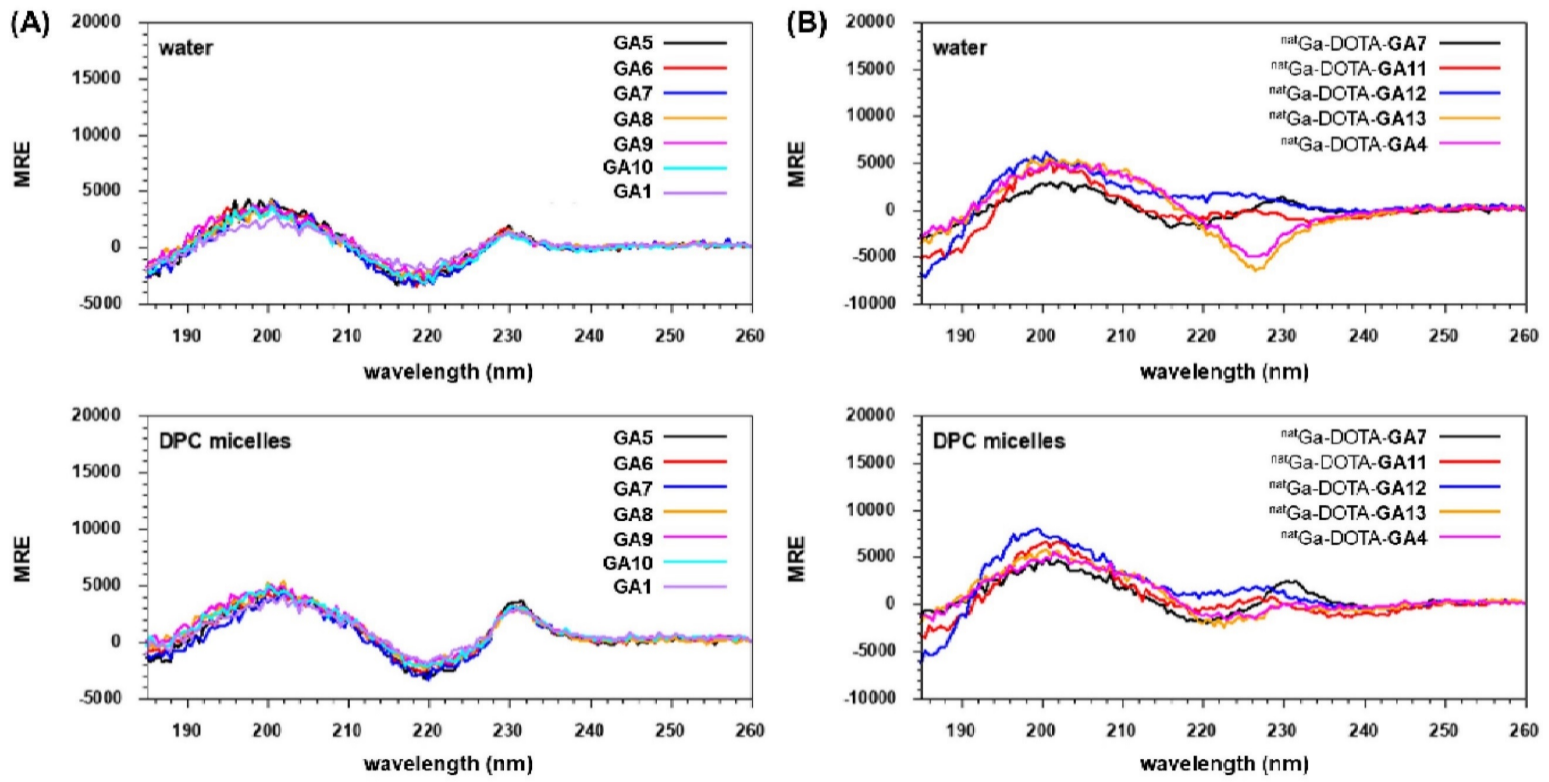
CD Spectroscopy of **(A)** *N*-terminal free linear peptides **GA1, GA5-GA10** and **(B)** natural gallium co-ordinated ^nat^Ga**-**DOTA-**GA4,** ^nat^Ga**-**DOTA-**GA7,** ^nat^Ga**-**DOTA-**GA11**-**GA13** in water and DPC micelles.

*N*-terminal free peptides **GA1**, **GA5**-**GA10,** and natural gallium co-ordinated peptides ^nat^Ga**-**DOTA-**GA4,** ^nat^Ga**-**DOTA-**GA7**, ^nat^Ga**-**DOTA-**GA11**-**GA13** were analyzed by CD spectroscopy to investigate the impact of chemical structure changes on conformational preferences of the peptides (Figure 6). **GA1**, **GA5**-**GA10** presented CD spectra of fully folded β-hairpin peptides with two maxima at *ca.* 200 and 230 nm and a minimum at *ca.* 220, both in water and DPC micelles (Figure 6A) 34, 44. However, the minimum at *ca.* 220 nm and the maximum at 230 nm were more pronounced in DPC compared to water for the **GA1** peptide and its analogs (Figure 6A).

Interestingly, ^nat^Ga-DOTA-peptides demonstrated more pronounced differences between structural conformations during CD spectroscopy. ^nat^Ga**-**DOTA-**GA4** and its D-Ala substituted variant ^nat^Ga**-**DOTA-**GA13** exhibited one broad maximum at *ca.* 200 and a minimum at *ca.* 227 nm in water (Figure 6B). All other **GA1**, D-Ala modified analogs, and ^nat^Ga chelated peptides demonstrated the expected two maxima at *ca.* 200 and 230 nm and a minimum at *ca.* 220. However, the intensities of their respective maxima and minima varied in water. In DPC micelles, ^nat^Ga**-**DOTA-**GA7** presented the most intense maxima at *ca.* 230 nm and the most intense minima at *ca*. 220 nm in comparison to other peptides (Figure 6B).

All ^nat^Ga-DOTA-peptides also revealed the fingerprint of fully folded β-hairpin peptides with two maxima at *ca.* 200 and 230 nm and a minimum at *ca.* 220 in DPC micelles (Figure 6B). It has been suggested that peptides based on the gastrin hormone interact with the cell membrane in a way that induces the preferred conformation, thus facilitating recognition by CCK_2_R 35, 45. This is further supported by our CD experiments, where all evaluated peptides adopted intensified and distinct structural conformations in DPC micelles.

### LogD_7.4_ and Serum Protein Binding Assays

Lipophilicity, expressed as the octanol-water partition coefficient, constitutes an important property in drug action, influencing both pharmacokinetic and pharmacodynamics processes. The distribution coefficient (Log*D*_7.4_) of [^68^Ga]Ga-DOTA-peptides was determined by measuring radioactivity distribution in the aqueous phase (phosphate buffer pH_7.4_) and the organic phase (*n*-octanol).

Peptides in this series were radiolabeled according to the protocol outlined in supplementary data. All radiolabeled peptides displayed high hydrophilicity with low Log*D*_7.4_ values ranging from -4.1 to -2.5 (Table 2). A low Log*D*_7.4_ is an important parameter required in the development of peptide-based theranostics with a suitable biodistribution profile 46. Substitution of the D-Glu in [^68^Ga]Ga-DOTA-**GA4** by D-Ala in analogs [^68^Ga]Ga-DOTA-**GA7** and [^68^Ga]Ga-DOTA-**GA13** did not influence lipophilicity a great deal as demonstrated by their similar Log*D*_7.4_ values: -2.97 - -2.59 (Table 2). The reduced hydrophilicity of these peptides is likely due to the hydrophobicity afforded by the bulky naphthylalanine (Nal) residue shared by all three peptides. The contribution of the Nal residue towards the overall lipophilicity of the corresponding peptides can be further demonstrated by comparison to peptides [^68^Ga]Ga-DOTA-**GA11** and [^68^Ga]Ga-DOTA-**GA12** with *N*-methylated phenylalanine (^13^NMe-Phe) and phenylalanine (^13^Phe) respectively as their terminal residue. Both these peptides presented with decreased LogD*_7.4_* values in comparison to peptides [^68^Ga]Ga-DOTA-**GA4**, [^68^Ga]Ga-DOTA-**GA7**, and [^68^Ga]Ga-DOTA-**GA13** (Table 2).

Serum or plasma protein binding is recognized as an important factor in theranostic development as their interactions influence the pharmacokinetics and pharmacodynamics of viable candidates. Correlative relationships between lipophilicity and serum protein binding of radiotracers have previously been established, showing strong linear trends where protein binding in serum increases with increasing lipophilicity 47, 48. The [^68^Ga]Ga-DOTA-peptides examined in this series demonstrated a moderate to high degree of plasma protein binding (59-84%), with higher plasma protein binding corresponding to more lipophilic (higher LogD*_7.4_*) peptides (Table 2).

### In Vitro Metabolic Assays

Metabolic stability is an important determinant of the efficacy of peptide radiopharmaceuticals and their progression as promising theranostic candidates. *In vitro,* metabolic degradation through hepatic and renal enzymes can be assessed by utilizing liver and kidney tissue homogenates. Radiolabeled peptides [^68^Ga]Ga-DOTA-**GA4_,_** and D-Ala substituted variants [^68^Ga]Ga-DOTA-**GA7,** [^68^Ga]Ga-DOTA-**GA11-GA13** were incubated in liver and kidney mouse homogenates, in human serum at 37°C, and in HEPES buffer (pH 7.4) as a negative control. Following a time course experiment, the metabolites were analyzed using HPLC to quantify the % breakdown of the respective parent peptide.

**Figure 7:**
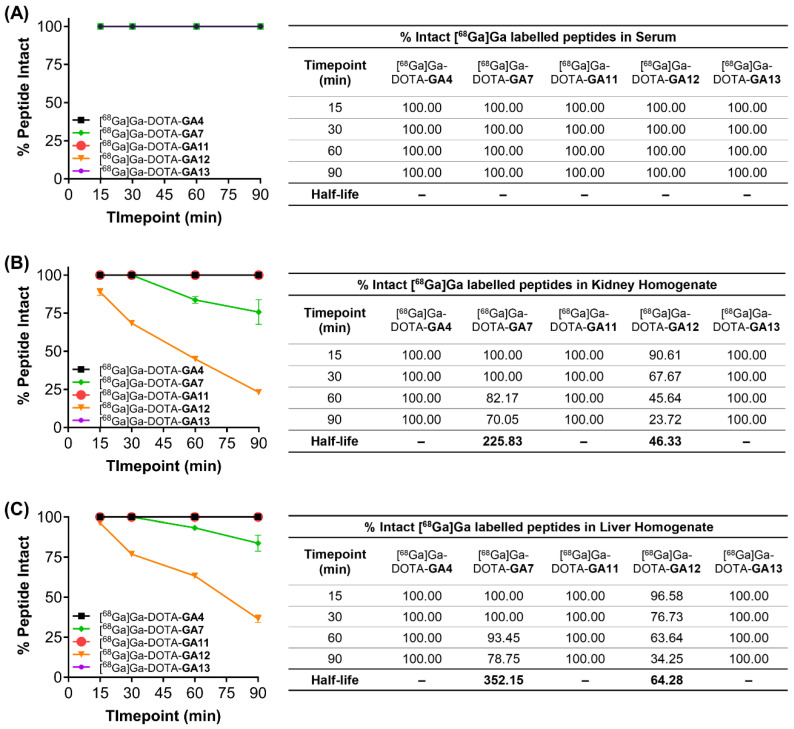
Metabolic stability of peptides [^68^Ga]Ga-DOTA-**GA4**, and D-Ala variants [^68^Ga]Ga-DOTA-**GA7,** [^68^Ga]Ga-DOTA-**GA11-GA13** incubated in (**A**) human serum, (**B**) kidney homogenates, and (**C**) liver homogenates. Samples were assayed at various time points over 90 min, and the intact peptides were assessed using liquid chromatography. The results are mean values (n=2). Half-life (t_1/2_) is displayed where appropriate in minutes.

All peptides tested remained intact when incubated with human serum and HEPES buffer at 37°C over the 90 min time course of the experiment (Figure 7A). [^68^Ga]Ga-DOTA-**GA7** and [^68^Ga]Ga-DOTA-**GA12** both underwent significant metabolism in liver and kidney homogenates over the course of the experiment (Figure 7B, 7C). [^68^Ga]Ga-DOTA-**GA12** was particularly prone to degradation, with approximately 35% peptide intact when incubated with liver microsomes (T_1/2_ 64.3 min) and only 20% peptide intact in kidney homogenates (T_1/2_ 46.3) after 90 min. [^68^Ga]Ga-DOTA-**GA7** demonstrated a more stable metabolic profile, with approximately 80% of peptide intact when incubated with liver microsomes (T_1/2_ 352.15 min) and 70% of the parent peptide intact in kidney homogenates at 90 min (T_1/2_ 225.83 min). The remaining peptides [^68^Ga]Ga-DOTA-**GA4**, [^68^Ga]Ga-DOTA-**GA11**, and [^68^Ga]Ga-DOTA-**GA13** were extremely stable in liver and kidney homogenates, with no observable metabolites detected at the 90 min timepoint (Figure 7).

These studies demonstrate the metabolically labile nature of the C-terminal phenylalanine residue on [^68^Ga]Ga-DOTA-**GA12**. Enzymatic cleavage at the Phe residue can be prevented by substituting the natural Phe moiety for the non-natural *N*-methylated Phe, as demonstrated in [^68^Ga]Ga-DOTA-**GA11**. The incorporation of *N*-methylated residues is a known strategy to enhance protease resistance 49-51. This is demonstrated yet again in [^68^Ga]Ga-DOTA-**GA7** and its *N*-methylated naphthylalanine variant at position 13, [^68^Ga]Ga-DOTA-**GA13**. Although not as prone to metabolic degradation as [^68^Ga]Ga-DOTA-**GA12**, *N*-methylation of the C-terminal naphthylalanine residue in [^68^Ga]Ga-DOTA-**GA13** prevents enzymatic degradation of the peptide, leaving 100% of the parent peptide intact.

### Small Animal PET Imaging and Biodistribution

In order to assess these promising ligands as potential theranostics, the library of [^68^Ga]Ga-DOTA-peptides was evaluated using static small animal PET imaging and quantifying biodistribution studies. This study was performed to investigate tumor uptake as well as the accumulation of radioligands in other organs. Mice bearing A431-CCK_2_R tumors were imaged at 1- and 2 hours post tracer injection, and tracer biodistribution was evaluated at the 1 hour and 2.5 hour time points to quantify tumor uptake and retention.

The work presented employed the A431-CCK_2_R/NSG mouse model; we have observed that this model consistently yields reduced tumor uptake of radioligands compared to the AR42J/Balbc model. This was demonstrated using the clinically relevant **CP04** and the lead **GA** peptide in this series, **GA4**. When tested in AR42J xenografts, **CP04** demonstrated only moderate tumor uptake at 5.5 ± 0.8% ID/g, and in the A431-CCK_2_R/NSG mouse model, **CP04** had a significantly lower tumor uptake of 2.5 ± 0.3% ID/g (Table 3). The marked difference between tumor uptake in the two models was more noticeably illustrated with **GA4**. Our previous work evaluated [^68^Ga]Ga-DOTA-**GA4** (**13b** as published in Corlett et al.) in AR42J tumor xenografts with a tumor uptake of 31.1 ± 5.3% ID/g 34. In this study, the same radioligand was assessed at a similar specific activity and time post-injection in the A431-CCK_2_R/NSG mouse model and showed a tumor uptake of only 8.77 ± 1.21% ID/g (Table 3).

The A431-CCK_2_R model reportedly has variable uptake of the same ligand in different studies. A study by Laverman and coworkers showed that ^111^In-radiolabeled **CP04** (reported molar activity: 11 MBq/nmol) had a tumor uptake of 9.7 ± 1.8% ID/g in an A431-CCK_2_R mouse model, while Roosenburg and coworkers investigated the same radioligand (reported molar activity: 30 MBq/nmol) and demonstrated a tumor uptake of 1.89 ± 0.74% ID/g in the same tumor model 43, 52. Despite the molar activity being lower for the study performed by Laverman and co-workers, the tumor uptake was higher than that observed by Roosenburg and co-workers. This comparison clearly demonstrates the variability in the A431-CCK_2_R model which cannot be explained only in terms of molar activity changes.

Differences in radioligand tumor uptake can also be attributed to different mouse models used in each investigation. Preclinical mouse models critically underpin early stages of the progression of a theranostic candidate. Differences in mouse size, physiology, and variable homology between models can produce inconsistent therapeutic outcomes. This is highlighted particularly in a comparative analysis of biodistribution of **PP-F11** in studies undertaken by Laverman and Roosenburg, who used athymic BALB/c and SCID/Bg mice respectively 43, 52.

Nevertheless, Roosenburg and co-workers reported that ^68^Ga-radiolabeled **CP04** had tumor uptake of 5.21 ± 2.19% ID/g in their A431-CCK_2_R mouse model at 1 hr post injection, which is analogous to our results 52. Collectively, the experimental and reported data confirm that the moderate uptake observed for **GA4** in this work could be due to variability in the model. A head-to-head comparison between **CP04** and **GA4** performed by Haskali and co-workers clearly confirms that **GA4** is far superior to **CP04** in tumor uptake (up to 6-fold increase in tumor uptake for the ^68^Ga-labeled counterparts). Thus, we have decided to benchmark all ongoing development presented herein against the optimal **GA4** scaffold. In the future, an independently performed head-to-head comparison of **GA4** and the reported developments in this manuscript should be done against **MGS5** to evaluate their relative performance.

The maximum standardised uptake value (SUV_max_) for the reference standard [^68^Ga]Ga-DOTA-**GA4** was 1.41, at 1 hour post-injection (Supplementary Data, pg. S51). The D-Ala substituted peptides [^68^Ga]Ga-DOTA-**GA7,** [^68^Ga]Ga-DOTA-**GA11-GA13** displayed a similar range of SUV_max_ (1.12-1.86) at the same time point (Supplementary Data, pg. S52-S55), highlighting the potency of all these ligands (Figure 7A). In particular, [^68^Ga]Ga-DOTA-**GA13**, which had SUV_max_ of 1.86, had a considerably higher tumor uptake compared to the reference compound [^68^Ga]Ga-DOTA-**GA4**.

At 1 hour post-injection, a tumor uptake of 8.77% ID/g was observed for [^68^Ga]Ga-DOTA-**GA4**. Its D-Ala substituted variant [^68^Ga]Ga-DOTA-**GA13** demonstrated a similarly high uptake of 9.68% ID/g (Table 4). There was a slight increase in tumor uptake at the 2.5 hour mark for [^68^Ga]Ga-DOTA-**GA13**, rising to 11.38% ID/g in contrast to [^68^Ga]Ga-DOTA-**GA4**, for which a small reduction to 7.54% ID/g was observed at the same time point. The remaining peptides [^68^Ga]Ga-DOTA-**GA7**, [^68^Ga]Ga-DOTA-**GA11-GA12** demonstrated good tumor uptake at both 1 hour (5.60-6.70% ID/g) and 2.5 hours (5.42-7.36% ID/g) post-injection (Table 4).

The specific uptake of [^68^Ga]Ga-DOTA-peptides was then demonstrated in blocking experiments by co-injection of the CCK_2_R antagonist YM022 (Figure 7B). Co-administration of YM022 reduced tumor uptake to 2.05-2.76% ID/g, indicating a >50% blockade at the 1 hour mark for all radiopeptides, with [^68^Ga]Ga-DOTA-**GA4** and analogue [^68^Ga]Ga-DOTA-**GA13** showing the most substantial reduction in uptake (Table 4). All radioligands tested presented low uptake in the stomach, an organ known to have high physiological CCK_2_R expression (Figure 7C) 53-56. Furthermore, all peptides investigated presented good renal clearance with low background uptake, leading to high tumor-to-background imaging ratios of 11.43 ± 0.90 for [^68^Ga]Ga-DOTA-**GA4** and 17.27 ± 1.84, 14.37 ± 2.23, 24.93 ± 4.57, and 19.33 ± 1.49 for D-Ala substituted peptides [^68^Ga]Ga-DOTA-**GA7** and [^68^Ga]Ga-DOTA-**GA11-GA13** respectively (Supplementary Data, pg. S51-S55). No substantial uptake of [^68^Ga]Ga-DOTA-peptides was observed in any other organ/healthy tissue, including blood, lungs, heart, muscle, spleen and liver, which eloquently presents the suitability of these radioligands for clinical translation (Table 5).

A major consideration in this investigation was to evaluate the impact of alanine substitution in the peptide scaffold on renal uptake of the radiolabeled peptides. High kidney uptake of radioligands is a significant issue, as it can lead to nephrotoxicity in patients. Renal uptake is primarily mediated by the resorption of the tracer or its metabolites through receptor or transporter-mediated processes 57-59. Various approaches have been employed to decrease kidney uptake and offer renal protection. These include the use of radioprotectors, utilizing albumin-binding moieties, and competitive inhibition *via* co-administration of positively charged amino acids such as L-lysine and L-arginine 60, 61. However, we believe that the most efficient and immediate method of reducing renal uptake is decreasing the radioligand's positive charge by fine-tuning the hexameric *N*-terminal sequence.

Biodistribution analysis of the series of radioligands confirmed that whilst [^68^Ga]Ga-DOTA-**GA4** showed a kidney uptake of 20.34% ID/g, this was reduced by 2-2.5 fold across the range of alanine substituted [^68^Ga]Ga-DOTA-**GA7**, and [^68^Ga]Ga-DOTA-**GA11-GA13** peptides to 8.67, 7.38, 4.52, 12.26% ID/g respectively (Table 5). These results confirmed that substituting alanine for glutamic acid in peptides significantly reduces renal uptake of peptides by lowering their overall ionic nature. In particular, [^68^Ga]Ga-DOTA-**GA12** demonstrated the highest tumor-to-kidney ratio (Figure 7D) with the lowest renal uptake (4.52% ID/g) observed in comparison to all other candidates evaluated, making it a truly promising candidate for clinical translation.

The *N*-methylated [^68^Ga]Ga-DOTA-**GA13** remained fully intact against enzymatic degradation compared to its non-methylated pair [^68^Ga]Ga-DOTA-**GA7**. This modification also improved tumor uptake of the radiopeptide in mice (Table 4) but resulted in an increase in renal uptake (Table 5). The peptides [^68^Ga]Ga-DOTA-**GA11** and [^68^Ga]Ga-DOTA-**GA12** share *N*-methylated Phe and free Phe terminal residues, respectively. Unlike the 1-Nal substituted peptides, *N*-methylation of [^68^Ga]Ga-DOTA-**GA11** gave reduced uptake in tumor and increased uptake in the kidneys compared to its non-methylated [^68^Ga]Ga-DOTA-**GA12**. This highlights the need to strike a careful balance between metabolic stability, renal uptake and tumor retention.

## Conclusion

Our work presents detailed and careful optimisation of peptide-based ligands for managing CCK_2_R-positive tumors. Through a series of meticulous optimisations to the C-terminus of **CP04**, we previously developed peptides with high binding affinity against CCK_2_R and high metabolic stability. This study presents the first exploration of how systemic replacement of each glutamic acid residue in the N-terminus with D-Ala affects the binding affinity to CCK2R. We identified that D-Ala substitution of the third D-Glu residue in the gastramide (**GA**) scaffold led to candidates with improved affinity, excellent metabolic stability and reduced overall ionic charge. In particular, peptides [^68^Ga]Ga-DOTA-**GA12** and [^68^Ga]Ga-DOTA-**GA13** demonstrated high tumor uptake with a significant reduction in renal uptake in comparison to the previous generation of peptides, making them truly exciting candidates that warrant clinical translation. This study marks a significant milestone in our understanding of the structure activity relationship of **CP04** based peptides for theranostic application against CCK_2_R.

## Supplementary Material

Supplementary materials and methods, results, images.

## Figures and Tables

**Figure 8:**
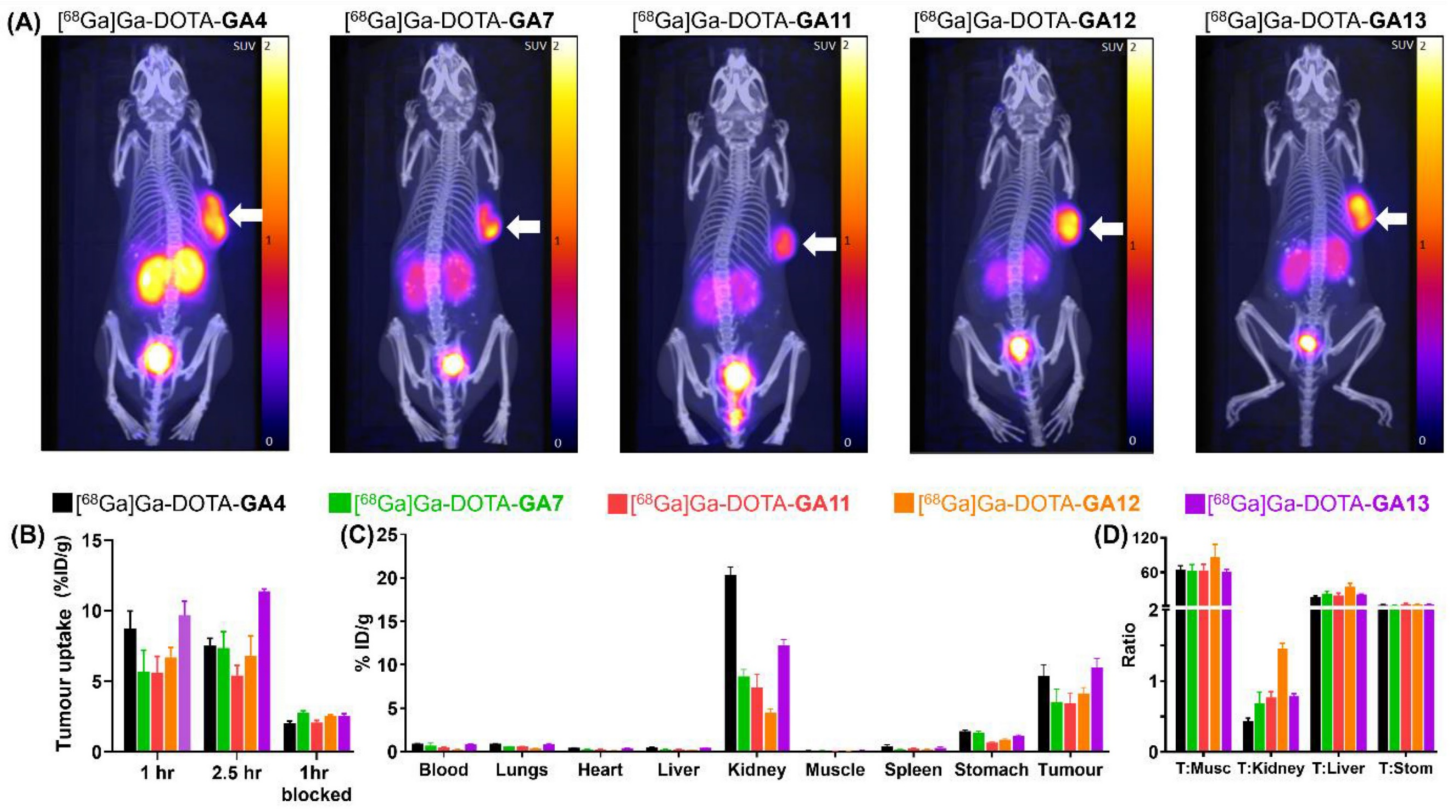
The imaging and biodistribution characteristics of radiolabeled standard [^68^Ga]Ga-DOTA-**GA4**, and analogs [^68^Ga]Ga-DOTA-**GA7,** [^68^Ga]Ga-DOTA-**GA11-GA13**. (**A**) Imaging of mice at 1 hour post radiolabeled peptide injection, showing the CCK_2_R positive tumor on the right flank. The highest tumor uptake was observed for [^68^Ga]Ga-DOTA-**GA4** and the alanine-substituted variant [^68^Ga]Ga-DOTA-**GA13**. (**B**) The tumor uptake study of [^68^Ga]Ga-DOTA-peptides at 1 hour, 2.5 hours, and 1 hour (blocked). (**C**) The biodistribution study of [^68^Ga]Ga-DOTA-peptides in different organs at 1 hour post-injection.(**D**) Tumor:Organ ratios of [^68^Ga]Ga-DOTA-peptides, notably showing reduced renal uptake and maintained high tumor uptake for [^68^Ga]Ga-DOTA-**GA12**. (T= tumor, Musc = muscle, Stom = stomach)

**Table 1:** Analytical data for all linear peptides generated in this study.

Peptide	Exact Mass (Calc.)	% Yield*^a^*	Mass obtained (mg)*^b^*	ESI-MS *m/z^c^*	HPLC Purity (%)*^d^*
GA1	1721.71	35.00	43.21	830.90	>95%
GA5	1663.70	20.68	22.60	831.80	94%
GA6	1663.70	21.68	27.24	830.90	>95%
GA7	1663.70	37.56	47.62	831.15	94%
GA8	1663.70	36.64	44.82	830.90	>95%
GA9	1663.70	32.92	42.10	830.90	>95%
GA10	1663.70	22.32	27.03	867.15	>95%
GA4	1735.73	25.20	31.39	831.15	>95%
GA11	1627.70	32.04	41.34	806.05	>95%
GA12	1613.69	32.36	39.87	838.15	>95%
GA13	1677.72	31.76	41.11	830.90	>95%

*^a^*% Yield reported represents % pure peptide obtained following assembly and subsequent HPLC purification. *^b^* Mass Obtained (mg) represents the mass of pure peptide obtained after HPLC purification. *^c^* ESI-MS base peak corresponds to [M-2H]^2+^. *^d^* Analytical conditions: Kinetex C18 XB 5µm 4.6x150mm, flow rate 1.5mL/min, gradient 15-90% MeCN in water containing 0.1% formic acid over 10 min with column heating at 40°C.

**Table 2:** Log*D*_7.4_ and protein binding (%) of [^68^Ga]Ga**-**DOTA-**GA4**, [^68^Ga]Ga**-**DOTA-**GA7**, [^68^Ga]Ga-DOTA-**GA11-GA13**. Log*D*_7.4_ values (n=5) obtained after incubation in PBS/*n*-octanol mix. Serum protein binding results after peptides were incubated with pooled human serum for 1 h at 37^o^C.

Peptide	Log*D*_7.4_	Plasma Protein Binding (%)
[^68^Ga]Ga-DOTA-**GA4**	-2.97 ± 0.13	82.43 ± 0.38
[^68^Ga]Ga-DOTA-**GA7**	-2.88 ± 0.09	78.69 ± 2.11
[^68^Ga]Ga-DOTA-**GA11**	-3.56 ± 0.01	59.75 ± 1.04
[^68^Ga]Ga-DOTA-**GA12**	-4.06 ± 0.14	63.62 ± 0.45
[^68^Ga]Ga-DOTA-**GA13**	-2.59 ± 0.02	83.16 ± 0.13

**Table 3:** Comparison of tumor uptake of [^68^Ga]Ga labeled **CP04** and **GA4** in AR42J and A431-CCK_2_R xenograft models. All data tumor uptake values are 1 hour post-injection. **GA4** exhibited reduced uptake in A431-CCK_2_R xenografts when treated at the same dose as in AR42J xenografts. ^1^Data shown for [^177^Lu]Lu-**CP04**.

	AR42J		A431-CCK_2_R	
	**CP04 (% ID/g)**	**GA4 (% ID/g)**	**CP04 (% ID/g)^1^**	**GA4 (% ID/g)**
Tumor	5.5 ± 0.8 34	31.1 ± 5.3 34	2.5 ± 0.3 62	8.8 ± 1.2

**Table 4:** Uptake of radiolabeled peptides in tumors of mice at 1 hour, and 2.5 hours post-injection. Blockade of radiolabeled peptides reported at 1 hour post-injection. Mice treated with CCK_2_R antagonist **YM022** at dose of 3mg/Kg dose for blockade experiments.

	Uptake in A431-CCK_2_R Tumors (% ID/g)			
**Peptide**	**1 hr**	**2.5 hr**	**1 hr Blocked**	**Blockade (1hr)**
[^68^Ga]Ga-DOTA-**GA4**	8.77 ± 1.21	7.54 ± 0.50	2.05 ± 0.14	77%
[^68^Ga]Ga-DOTA-**GA7**	5.71 ± 1.50	7.36 ± 1.16	2.76 ± 0.14	52%
[^68^Ga]Ga-DOTA-**GA11**	5.60 ± 1.16	5.42 ± 0.70	2.07 ± 0.15	63%
[^68^Ga]Ga-DOTA-**GA12**	6.70 ± 0.69	6.81 ± 1.40	2.53 ± 0.11	62%
[^68^Ga]Ga-DOTA-**GA13**	9.68 ± 1.02	11.38 ± 0.17	2.53 ± 0.17	74%

**Table 5:** The biodistribution of radiolabeled standard [^68^Ga]Ga-DOTA-**GA4**, and analogs [^68^Ga]Ga-DOTA-**GA7,** [^68^Ga]Ga-DOTA-**GA11-GA13** in key organs of mice 1 hour post-injection (n=3).

	Uptake in Organs (% ID/g)				
**Organ**	[^68^Ga]Ga-DOTA-**GA4**	[^68^Ga]Ga-DOTA-**GA7**	[^68^Ga]Ga-DOTA-**GA11**	[^68^Ga]Ga-DOTA-**GA12**	[^68^Ga]Ga-DOTA-**GA13**
Blood	0.92 ± 0.05	0.68 ± 0.31	0.46 ± 0.08	0.29 ± 0.01	0.83 ± 0.07
Lungs	0.92 ±0.04	0.57 ± 0.03	0.59 ± 0.02	0.34 ± 0.04	0.85 ± 0.11
Heart	0.45 ± 0.03	0.30 ± 0.02	0.27 ± 0.08	0.14 ± 0.01	0.40 ± 0.03
Liver	0.51 ± 0.01	0.30 ± 0.01	0.29 ± 0.01	0.19 ± 0.02	0.44 ± 0.03
Kidneys	20.34 ± 0.89	8.67 ± 0.80	7.38 ± 1.54	4.52 ± 0.38	12.26 ± 0.63
Muscle	0.14 ± 0.01	0.12 ± 0.02	0.09 ± 0.02	0.09 ± 0.02	0.16 ± 0.03
Spleen	0.62 ± 0.14	0.24 ± 0.02	0.34 ± 0.08	0.25 ± 0.04	0.43 ± 0.13
Stomach	2.37 ± 0.12	2.22 ± 0.14	1.04 ± 0.06	1.38 ± 0.08	1.81 ± 0.12
Tumor	8.77 ± 1.21	5.71 ± 1.50	5.60 ± 1.16	6.70 ± 0.69	9.68 ± 1.02
