# Supplementary material for: Precision peptide theranostics: developing N- to C-terminus optimized theranostics targeting cholecystokinin-2 receptor
Source: Theranostics. 2024 Feb 24;14(5):1815–28. doi: 10.7150/thno.89701 (PMC10945332; doi:10.7150/thno.89701)
Supplement: Supplementary file 1 — Supplementary materials and methods, results, images. [file thnov14p1815s1.pdf]

# Precision peptide theranostics: developing *N*- to *C*-terminus optimized theranostics targeting cholecystokinin-2 receptor

*Authors:* Marwa N. Rahimi[1,2], Alicia Corlett[3], Jessica Van Zuylekom[4], Marc Antoine Sani[5], Benjamin Blyth[4], Philip Thompson[6], Peter D. Roselt[1,2], Mohammad B. Haskali[1,2]

[1] *Department of Radiopharmaceutical Sciences, Cancer Imaging, The Peter MacCallum Cancer Centre, Victoria 3000, Australia;*
[2] *Sir Peter MacCallum Department of Oncology, The University of Melbourne, Victoria 3010, Australia.*
[3] *Cancer Imaging, Peter MacCallum Cancer Centre, Melbourne, Victoria 3000, Australia.*
[4] *Models of Cancer Translational Research Centre, The Peter MacCallum Cancer Centre, Victoria 3000, Australia.*
[5] *The Bio21 Institute, School of Chemistry, The University of Melbourne, Melbourne, Victoria, 3010 Australia*
[6] *Medicinal Chemistry, Monash Institute of Pharmaceutical Sciences, Faculty of Pharmacy and Pharmaceutical Sciences, Monash University (Parkville Campus), Parkville, Victoria 3052, Australia.*

Materials and Methods: Chemistry ..... S4
- General Methods and Reagents ..... S4
- Peptide Synthesis and Purification: General method ..... S4
- Peptide Synthesis and Purification of *N*-terminal free peptides: GA1, GA5–GA10 ..... S6
- Peptide Synthesis and Purification of *N*-terminal free peptides: GA4, GA7, GA11–GA13 ..... S7
- Peptide Synthesis and Purification of DOTA coupled peptides: DOTA-GA4, DOTA-GA7, DOTA-GA11–GA13 ..... S7
- Synthesis and Purification of $^{nat}$Ga co-ordinated peptides: $^{nat}$Ga-DOTA-GA4, $^{nat}$Ga-DOTA-GA7, $^{nat}$Ga-DOTA-GA11–GA13 ..... S8
- Radiolabeling peptides: [$^{68}$Ga]Ga-DOTA-GA4, [$^{68}$Ga]Ga-DOTA-GA7, [$^{68}$Ga]Ga-DOTA-GA11–GA13 ..... S9
- Radiosynthesis: [$^{177}$Lu]Lu-DOTA-CP04 (PP-F11) ..... S10
- Quality control: [$^{68}$Ga]Ga-DOTA-GA4, [$^{68}$Ga]Ga-DOTA-GA7, [$^{68}$Ga]Ga-DOTA-GA11–GA13 S11

Chemistry Remarks and Results ..... S12
- Quality Control Specifications of Ga-68 labelled peptides ..... S12
- Radiolabeled peptide characterisation ..... S13
- Spectral data of Synthesised Peptides ..... S14
  - N-terminal free GA1 ..... S14
  - N-terminal free GA5 ..... S15
  - N-terminal free GA6 ..... S16
  - N-terminal free GA7 ..... S17
  - N-terminal free GA8 ..... S18
  - N-terminal free GA9 ..... S19
  - N-terminal free GA10 ..... S20
  - N-terminal free GA4 ..... S21

N-terminal free GA11 ... S22
N-terminal free GA12 ... S23
N-terminal free GA13 ... S24
DOTA-GA4 ... S25
DOTA-GA7 ... S26
DOTA-GA11 ... S27
DOTA-GA12 ... S28
DOTA-GA13 ... S29
Radiolabeled [$^{68}$Ga]Ga-DOTA-GA4 ... S30
Radiolabeled [$^{68}$Ga]Ga-DOTA-GA7 ... S31
Radiolabeled [$^{68}$Ga]Ga-DOTA-GA11 ... S32
Radiolabeled [$^{68}$Ga]Ga-DOTA-GA12 ... S33
Radiolabeled [$^{68}$Ga]Ga-DOTA-GA13 ... S34
Materials and Methods: Biology ... S35
Circular Dichroism (CD) ... S35
*In Vitro* $CCK_2R$-Ligand Competitive Binding Assays ... S35
LogD7.4 Determination ... S36
Protein Binding and Free Fraction Determination ... S36
Cell Internalisation Experiments ... S37
Metabolic Studies ... S37
Small Animal PET Imaging and Biodistribution Studies ... S38
Biological assays results ... S40
Metabolism studies: [$^{68}$Ga]Ga-DOTA-GA4 ... S40
HEPES (control) ... S40
Serum ... S41
Kidney ... S41
Liver ... S41
Metabolism studies: [$^{68}$Ga]Ga-DOTA-GA7 ... S43
HEPES (control) ... S43
Serum ... S43
Kidney ... S44
Liver ... S44
Metabolism studies: [$^{68}$Ga]Ga-DOTA-GA11 ... S45
HEPES (control) ... S45
Serum ... S45
Kidney ... S46
Liver ... S46
Metabolism studies: [$^{68}$Ga]Ga-DOTA-GA12 ... S47
HEPES (control) ... S47

Serum ........ S47
Kidney ........ S48
Liver ........ S48
Metabolism studies: [$^{68}$Ga]Ga-DOTA-GA13 ........ S49
HEPES (control) ........ S49
Serum ........ S49
Kidney ........ S50
Liver ........ S50
Imaging SUV: [$^{68}$Ga]Ga-DOTA-GA4 ........ S51
Imaging SUV: [$^{68}$Ga]Ga-DOTA-GA7 ........ S52
Imaging SUV: [$^{68}$Ga]Ga-DOTA-GA11 ........ S53
Imaging SUV: [$^{68}$Ga]Ga-DOTA-GA12 ........ S54
Imaging SUV: [$^{68}$Ga]Ga-DOTA-GA13 ........ S55

# Materials and Methods: Chemistry

## General Methods and Reagents

Reagents and solvents were purchased in their highest purified condition from Sigma-Aldrich, Merck (Germany), Macrocyclics, Fluka and NovaBiochem (Germany). Purified water was obtained from an in-house Millipore water purification system. Amino acids used were purchased from GL Biochem (China) and ChemPep (USA). Anhydrous solvents were used in peptide synthesis. Anhydrous ether, dichloromethane (DCM), and triethylamine (TEA) were obtained from Sigma Aldrich.

Radioactivity measurements of radiolabeled peptides were carried out with a CRC-15PET dose calibrator (Capintec) that was calibrated daily using Cs-137 and Co-57 sources (Isotope Products Laboratories). Analytical Radio-HPLC was performed using a Shimadzu HPLC system consisting of a SCL-10AVP system controller, SIL-0ADVP auto-injector, LC-10 ATVP solvent delivery unit, CV-10AL control valve, DGU-14A degasser, and SPD-10AVPV detector. This was coupled to a radiation detector consisting of an Ortec model 276 photomultiplier base with a 925-SCINTACE-mate preamplifier, amplifier, bias supply, and SCA and a Bicron 1M11/2 photomultiplier tube.

## Peptide Synthesis and Purification: General method

Peptides were assembled using standard Fmoc-based solid phase peptide synthesis (SPPS) procedures using Rink amide resin (0.8 mmol/g; ChemPep, Florida, USA) on an automated CEM Liberty Blue microwave peptide synthesizer (John Morris Group, Victoria, Australia). Peptides were assembled on a 0.1 mmol reaction scale. Fmoc-deprotection was performed in two stages as follows: peptide-resin was treated with 20% piperidine/DMF (v/v; 5 mL) containing oxyma (0.1 M) under microwave irradiation for 30 s (40 W, 40°C), followed by filtration and a second treatment of the same deprotection cocktail under microwave irradiation

(45 W, 75°C; 3 min). The peptide-resins were then rinsed with DMF (3 x 4 mL). Coupling of all standard Fmoc-amino acids was achieved by the addition of Fmoc-amino acid (5 eq, 0.5 mmol), DIC (5 eq) and oxyma (10 eq) in DMF (4 mL) to the Nα-deprotected peptide-resin and the mixture agitated under microwave radiation for 3 min (30 W, 90°C). Following sequence assembly, the peptide-resins were rinsed manually with dichloromethane (DCM) (3 x 5 mL) prior to the cleavage step. Global deprotection and cleavage of peptides from the solid support was performed using a TFA:TIPS:$H_2O$:DODT:1,3-dimethoxybenzene (85:5:5:2.5:2.5, % v/v/v/v/v; 4 mL) cocktail for 2 h. The cleavage mixtures were then filtered, the TFA solutions evaporated under $N_2$ flow, and the crude products isolated by trituration and subsequent washes with di-ethyl ether (3 x 30 mL).

Once crude peptide was isolated, they were purified using the preparative HPLC system Agilent 1290 Infinity II. Quality control analysis of the isolated peptides was performed using an analytical HPLC/MS system (Shimadzu HPLC system, SIL-20A HT auto-injector, LCMS-8030 Triple Quadrupole Mass Spectrometer) using a Phenomenex Kinetex XB-C18 Sum 150 mm × 4.6 mm 5 µm column.

Pure fractions were combined and lyophilised to afford the corresponding pure peptide as an amorphous white solid.

**Peptide Synthesis and Purification of *N*-terminal free peptides: GA1, GA5–GA1**0

All peptides in this series (Figure S1) were synthesised following **Peptide Synthesis and Purification: General methods.** All peptides were purified *via* HPLC using on a Phenomenex Gemini 5µm C18 110Å LC Column 250 mm x 30mm AXD on a gradient of MeCN: 0.1% (v/v) TFA, starting at 30% MeCN for 1 min, increased to 70% over 20 min and then instantly increased to 90% and maintained at that for 2 min then back down to 30% MeCN, with a flow rate of 30mL/min.

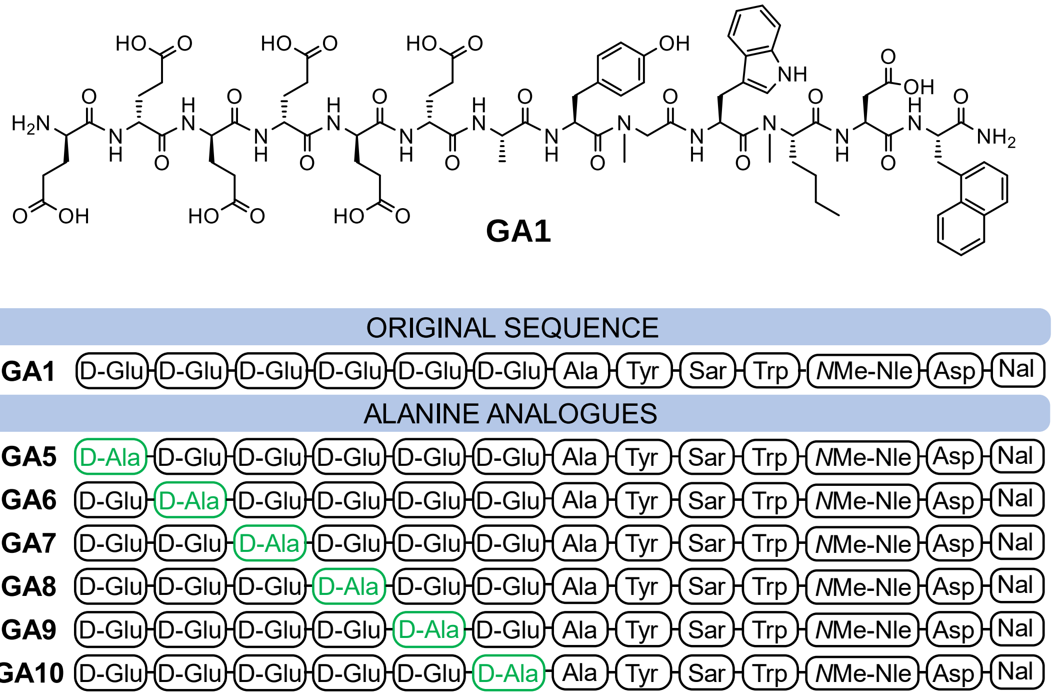


**Figure S1**: **GA1** structure and D-alanine (DAla) scan peptide sequences (**GA5–GA10**).

**Peptide Synthesis and Purification of *N*-terminal free peptides: GA4, GA7, GA11–GA13**

All peptides in this series (**GA4, GA7, GA11–GA13**) (Figure S2) were synthesised following **Peptide Synthesis and Purification: General methods**. Once crude peptides were obtained, they were purified using HPLC on a Phenomenex Gemini 5µm C18 110Å LC Column 250 mm x 30mm AXD on a gradient of MeCN: 0.1% (v/v) TFA, starting at 30% MeCN for 1 min, increased to 70% over 20 min and then instantly increased to 90% and maintained at that for 2 min then back down to 30% MeCN, all with a flow rate of 30mL/min.

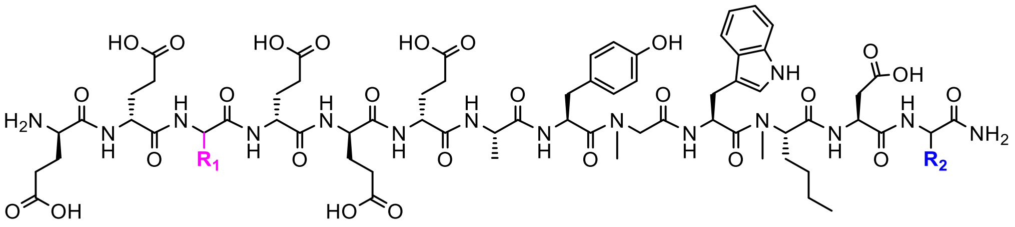

| **Peptide** | **$R_1$** | **$R_2$** |
|---|---|---|
| **GA4** | D-Glu | $N^{\alpha}$ Me-1-Nal |
| **GA7** | D-Ala | 1-Nal |
| **GA11** | D-Ala | $N^{\alpha}$ Me-L-Phe |
| **GA12** | D-Ala | L-Phe |
| **GA13** | D-Ala | $N^{\alpha}$ Me-1-Nal |

**Figure S2**: **GA4** and D-Ala substituted peptides **GA7, GA11–GA13**.

**Peptide Synthesis and Purification of DOTA coupled peptides: DOTA-GA4, DOTA-GA7, DOTA-GA11–GA13**

The conjugation of free DOTA to peptides was completed in solution as previously reported.[32] Briefly, DOTA (1.5 eq. relative to peptide) was pre-activated with *N*-hydroxysuccinimide (NHS) (2.25 eq.) using N-(3-dimethylaminopropyl)-N'-ethylcarbodiimide hydrochloride (EDCI) (2.25 eq.) and N,N-diisopropylethylamide (DIPEA) (3 eq.) in anhydrous dimethyl sulfoxide (DMSO) (typically 500 µL). The reaction mixture was sonicated at 50 °C for 30 min until all starting material dissolved. *N*-terminal free peptides

**GA4, GA7, GA11–GA13** (1 eq.) were then dissolved in a minimum volume of anhydrous DMSO (typically 200–500 µL). The resulting mixture was shaken for 30 min at room temperature and the reaction progress was monitored by LC-MS analysis. When reactions were complete (typically within 1 hour), as indicated by the disappearance of the *m/z* peak of the starting peptide by MS analysis, distilled water was added to form a 20:80 mixture of DMSO:water and the crude peptide was purified using reverse phase HPLC. HPLC conditions for the purification of DOTA-**GA4**, DOTA-**GA7**, DOTA-**GA11–GA13** (Figure S3) were identical to those reported above for the purification of their respective *N*-terminus free peptides.

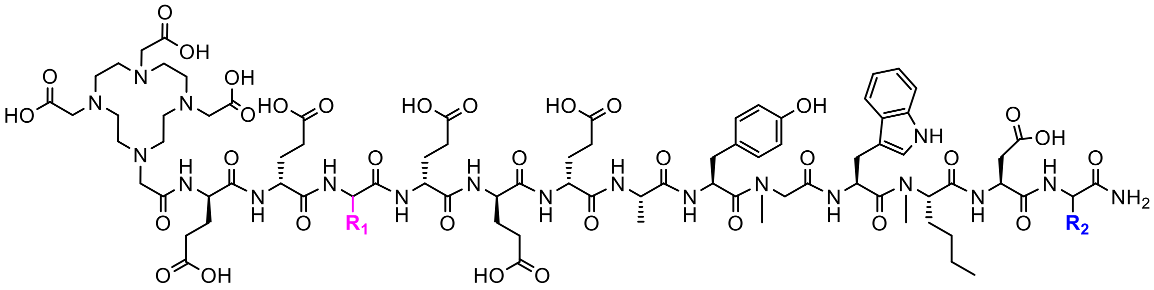

| **Peptide** | **R₁** | **R₂** |
|---|---|---|
| DOTA-**GA4** | D-Glu | $N^{\alpha}$ Me-1-Nal |
| DOTA-**GA7** | D-Ala | 1-Nal |
| DOTA-**GA11** | D-Ala | $N^{\alpha}$ Me-L-Phe |
| DOTA-**GA12** | D-Ala | L-Phe |
| DOTA-**GA13** | D-Ala | $N^{\alpha}$ Me-1-Nal |

**Figure S3**: Chemical structures for DOTA conjugated peptides, DOTA-**GA4,** DOTA-**GA7,** DOTA-**GA11–GA13**.

## Synthesis and Purification of $^{nat}$Ga co-ordinated peptides: $^{nat}$Ga-DOTA-GA4, $^{nat}$Ga-DOTA-GA7, $^{nat}$Ga-DOTA-GA11–GA13

The chelation of $^{nat}$Gallium to DOTA-peptides DOTA-**GA4,** DOTA-**GA7,** DOTA-**GA11–GA13** (Figure S3) was performed in accordance with previously published procedure.[32, 41] Briefly, DOTA-peptides (2–8 µmol) were suspended in freshly prepared ascorbic acid buffer

(3–5 mL of 50mg/mL), pH adjusted to 4.5 using a concentrated solution of NaOH (5M). Gallium nitrate (4 eq. relative to DOTA-peptide) was then added, and the resulting mixture was heated at 95°C for 15 min. Following this, the mixture was cooled to room temperature, centrifuged to remove any precipitate, and purified using reverse phase HPLC adopting the same chromatographic conditions for the purification of the respective *N*-terminus free peptides. Using these conditions, we obtained $^{nat}$Ga-DOTA-**GA4,** $^{nat}$Ga-DOTA-**GA7,** $^{nat}$Ga-DOTA-**GA11**–**GA13** (Figure S4).

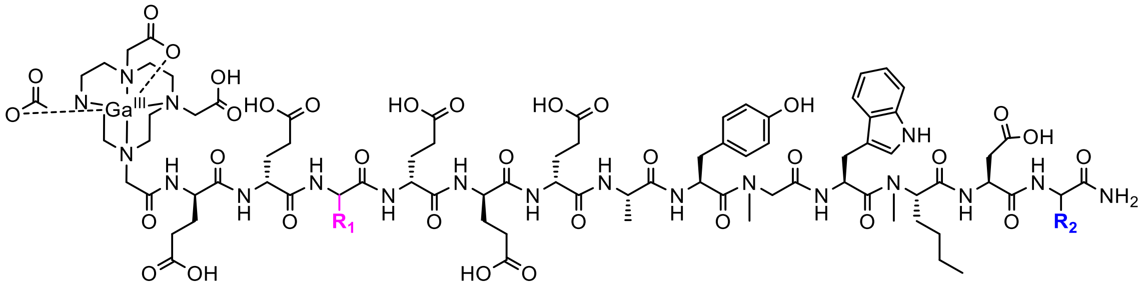


| **Peptide** | **R1** | **R2** |
|---|---|---|
| $^{nat}$Ga-DOTA-**GA4** | D-Glu | $N^{\alpha}$ Me-1-Nal |
| $^{nat}$Ga-DOTA-**GA7** | D-Ala | 1-Nal |
| $^{nat}$Ga-DOTA-**GA11** | D-Ala | $N^{\alpha}$ Me-L-Phe |
| $^{nat}$Ga-DOTA-**GA12** | D-Ala | L-Phe |
| $^{nat}$Ga-DOTA-**GA13** | D-Ala | $N^{\alpha}$ Me-1-Nal |

**Figure S4**: Chemical structures for natural gallium conjugated peptides $^{nat}$Ga-DOTA-**GA4,** $^{nat}$Ga-DOTA-**GA7,** $^{nat}$Ga-DOTA-**GA11–GA13**.

**Radiolabeling peptides: [$^{68}$Ga]Ga-DOTA-GA4, [$^{68}$Ga]Ga-DOTA-GA7, [$^{68}$Ga]Ga-DOTA-GA11–GA13**

DOTA-**GA4**, DOTA-**GA7**, DOTA-**GA11–GA13** were radiolabeled in accordance with our published procedure.[32, 41] Briefly, a mixture of the corresponding DOTA-peptide precursor (30 µg) in 0.5 M sodium acetate solution (800 µL), ethanol (200 µL), 0.05 M sodium ascorbate (200 µL), 0.05 M 2,5-dihydroxybenzoic acid sodium salt (200 µL) and 10 mg/mL methionine (100 µL) was freshly prepared before radiosynthesis. The reaction mixture was then transferred

into the reactor of an iPHASE MultiSyn radiochemistry module. Gallium-68 was then delivered to the reaction vessel by elution of an ITG $^{68}$Ge/$^{68}$Ga generator using 0.05 M HCl (4 mL). The reaction mixture was then heated to 90 °C for 8 minutes (pH 4.5) and then diluted with water (5 mL). The gallium-68 labelled peptide was then trapped on a Strata-X RP SPE cartridge. The trapped product was rinsed with water (5 mL), eluted with ethanol (~ 0.5 mL) and diluted with saline (9 mL) to afford [$^{68}$Ga]Ga-DOTA-**GA4** (Figure S5), and D-Ala substituted variants [$^{68}$Ga]Ga-DOTA-**GA7**, [$^{68}$Ga]Ga-DOTA-**GA11–GA13** (Figure S5) in a final formulation with less than ≤10% ethanol in saline at activities of 380-700MBq.

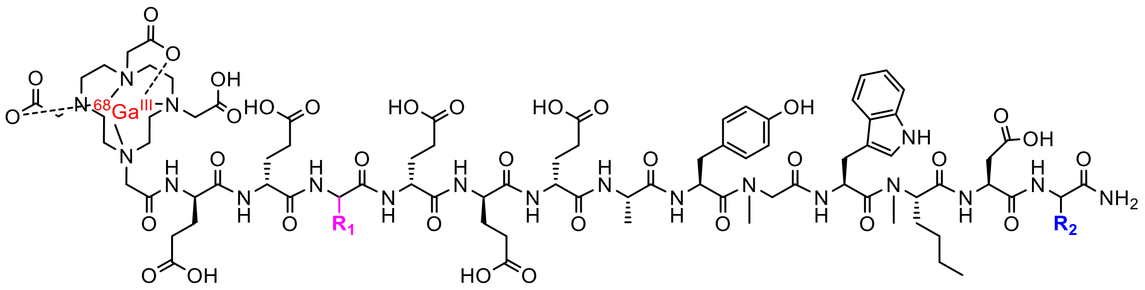

| Peptide | $R_1$ | $R_2$ |
|---|---|---|
| [$^{68}$Ga]Ga-DOTA-**GA4** | D-Glu | $N^{\alpha}$ Me-1-Nal |
| [$^{68}$Ga]Ga-DOTA-**GA7** | D-Ala | 1-Nal |
| [$^{68}$Ga]Ga-DOTA-**GA11** | D-Ala | $N^{\alpha}$ Me-L-Phe |
| [$^{68}$Ga]Ga-DOTA-**GA12** | D-Ala | L-Phe |
| [$^{68}$Ga]Ga-DOTA-**GA13** | D-Ala | $N^{\alpha}$ Me-1-Nal |

**Figure S5**: Chemical structures for Gallium-68 radiolabeled DOTA conjugated peptides [$^{68}$Ga]Ga-DOTA-**GA4,** [$^{68}$Ga]Ga-DOTA-**GA7,** and [$^{68}$Ga]Ga-DOTA-**GA11–GA13**.

## Radiosynthesis: [$^{177}$Lu]Lu-DOTA-CP04 (PP-F11)

DOTA-**CP04** (30 µg, 14.6 nmol) dissolved in sodium acetate (0.5 M, 100µL (0.3µg/µL peptide solution)) was constituted in 0.4 M ammonium acetate/0.24 M 2,5-dihydroxybenzoic acid (200 µL, pH 4.5) containing ethanol (50 µL), L-methionine (50 µL of 10 mg/mL solution in milliQ water) and sodium ascorbate (50 µL, 0.05M in milliQ water). 200–500 MBq of non-carrier added [$^{177}$Lu]$LuCl_3$ in 0.04 M HCl (50–100 µL) was added to this mixture and the resulting solution was heated at 80 °C for 30 min. A small fraction (10–40 µL containing ca. 20 MBq)

of this was dispensed into binding buffer (DMEM + 1% foetal bovine serum) (5 ml) to give [$^{177}$Lu]Lu-DOTA-**CP04** (Figure S6) ready for cell binding assays.

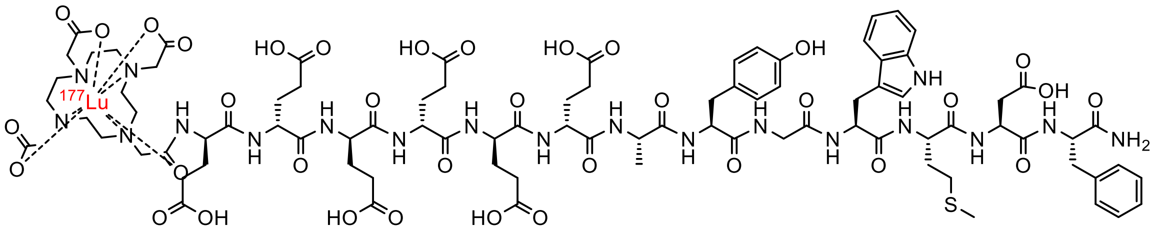

**Figure S6**: Chemical structure of Lutetium-177 labelled DOTA-CP04, [$^{177}$Lu]Lu-DOTA-**CP04**.

**Quality control: [$^{68}$Ga]Ga-DOTA-GA4, [$^{68}$Ga]Ga-DOTA-GA7, [$^{68}$Ga]Ga-DOTA-GA11–GA13**

Final formulation appearance, pH, radionuclidic identity (half-life test), purity and radiochemical identity were assessed for each of the radiolabeled peptides produced (see Table S1 for specifications and results summary). Radiochemical identity and purity of Ga-68 labelled peptides was assessed by radio-HPLC. Radiochemical identity was confirmed by matching retention time (and co-mobility) of the gallium-68 labelled peptides and their respective non-radioactive reference standards (chelated with $^{nat}$gallium) (Table S1). The radiochemical purity was identified by integration of all observed radioactive peaks and comparison of their relative percentage area (Table S1).

Radiochemical purity was further analysed using Radio-TLC. A small drop (ca. 2µL) of the gallium-68 labelled peptides was placed on an iTLC-SG strip (2 cm width × 10 cm length) and then processed in a chamber containing 7.7% w/v ammonium acetate ($NH_4OAc$) in methanol (1:1) as mobile phase. The iTLC-SG strip was then removed and placed into a Raytest TLC reader to determine percentage area of radioactivity as free gallium-68 and as labelled peptide.

# Chemistry Remarks and Results

## Quality Control Specifications of Ga-68 labelled peptides

**Table S1**: Acceptable quality control specifications of the produced [$^{68}$Ga]Ga-labelled peptides and the observed respective results. Specific activity of radiopharmaceuticals used in our experiments varied depending on the amount of Ga-68 eluted from the ITG 68Ge/68Ga generator, which is contingent on its age.

| **Parameters** | **Acceptable specifications** | [$^{68}$Ga]Ga-DOTA-**GA4** | [$^{68}$Ga]Ga-DOTA-**GA7** | [$^{68}$Ga]Ga-DOTA-**GA11** | [$^{68}$Ga]Ga-DOTA-**GA12** | [$^{68}$Ga]Ga-DOTA-**GA13** |
|---|---|---|---|---|---|---|
| Appearance | Clear & colourless | pass | pass | pass | pass | pass |
| pH | 4-8 | 5-6 | 5-6 | 5-6 | 5-6 | 5-6 |
| Radionuclidic identity (half-life) | 62-74 min | 63.0 min | 63.0 min | 69.3 min | 65.0 min | 63.0 min |
| Radionuclidic identity (retention time) | Reference standard ± 1 min | Ref stnd: 4.79 min Product: 4.84 min | Ref stnd: 4.82 min Product: 4.92 min | Ref stnd: 4.60 min Product: 4.66 min | Ref stnd: 4.60 min Product: 4.60 min | Ref stnd:4.75 min Product: 4.88 min |
| ***Radiochemical purity (HPLC) | ≥ 90% labelled peptide | 95-98% | 92-96% | 93-96% | 92-94% | 93-95% |
| Radiochemical purity (TLC) | ≥ 98% labelled peptide | > 98% | > 98% | > 98% | > 98% | > 98% |
| | ≤ 2% free Ga-68 | < 2% | < 2% | < 2% | < 2% | < 2% |
| Specific activity | ≥ 3.0 MBq/µg | 7-10 MBq/µg | 7-10 MBq/µg | 7-10 MBq/µg | 7-10 MBq/µg | 7-10 MBq/µg |

## Radiolabeled peptide characterisation

**Table S2**: Analytical data for DOTA coupled peptides.

| **Peptide** | **% Yield**[a] | **Exact Mass (Calc.)** | **Retention time (min)** | **ESI-MS (*m/z*)** [a] | **HPLC Purity (%)**[a] |
|---|---|---|---|---|---|
| DOTA-**GA4** | 79.20 | 2121.91 | 5.11 | 1060.20 | >95% |
| DOTA-**GA7** | 75.46 | 2049.88 | 5.08 | 1024.05 | >95% |
| DOTA-**GA11** | 64.03 | 2013.88 | 4.79 | 1006.20 | >95% |
| DOTA-**GA12** | 86.66 | 1999.87 | 4.79 | 999.55 | >95% |
| DOTA-**GA13** | 78.89 | 2063.90 | 5.16 | 1031.20 | >95% |

[a] ESI-MS base peak corresponds to $[M-2H]^{2+}$. Analytical conditions: Kinetex C18 XB 5µm 4.6x150mm, flow rate 1.5mL/min, gradient 15–90% MeCN in water containing 0.1% formic acid over 10 min with column heating at 40°C.

**Table S3**: Analytical data for natural gallium co-ordinated peptides.

| **Peptide** | **% Yield**[a] | **Exact Mass (Calc.)** | **Retention time (min)** | **ESI-MS (*m/z*)** [a] | **HPLC Purity (%)**[a] |
|---|---|---|---|---|---|
| $^{nat}$Ga-DOTA-**GA4** | 60.51 | 2188.82 | 4.79 | 1093.63 | >95% |
| $^{nat}$Ga-DOTA-**GA7** | 67.65 | 2116.79 | 4.82 | 1057.48 | >95% |
| $^{nat}$Ga-DOTA-**GA11** | 58.92 | 2080.79 | 4.60 | 1039.63 | >95% |
| $^{nat}$Ga-DOTA-**GA12** | 66.21 | 2066.78 | 4.60 | 1032.99 | >95% |
| $^{nat}$Ga-DOTA-**GA13** | 62.27 | 2188.82 | 4.75 | 1093.61 | >95% |

[a] ESI-MS base peak corresponds to $[M-2H]^{2+}$. Analytical conditions: Kinetex C18 XB 5µm 4.6x150mm, flow rate 1.5mL/min, gradient 15–90% MeCN in water containing 0.1% formic acid over 10 min with column heating at 40°C.

## Spectral data of Synthesised Peptides

### N-terminal free GA1

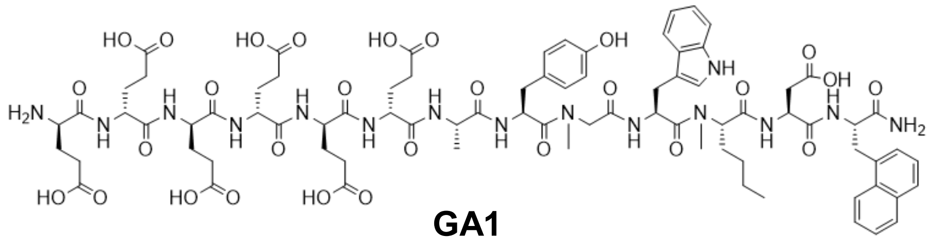

**GA1**

Exact Mass: 1721.71
Molecular Weight: 1722.78

SHIMADZU LabSolutions

# Analysis Report

<Sample Information>

| | | | |
|---|---|---|---|
| Sample Name | : GA1 | | |
| Method Filename | : 15to90ov7min_LCMS_210325.lcm | | |
| Batch Filename | : 20210330.lcb | | |
| Injection Volume | : 10 uL | | |
| Date Acquired | : 3/03/2022 1:27:01 PM | Acquired by | : Marwa Rahimi |
| Date Processed | : 18/05/2022 10:36:50 PM | Processed by | : Marwa Rahimi |

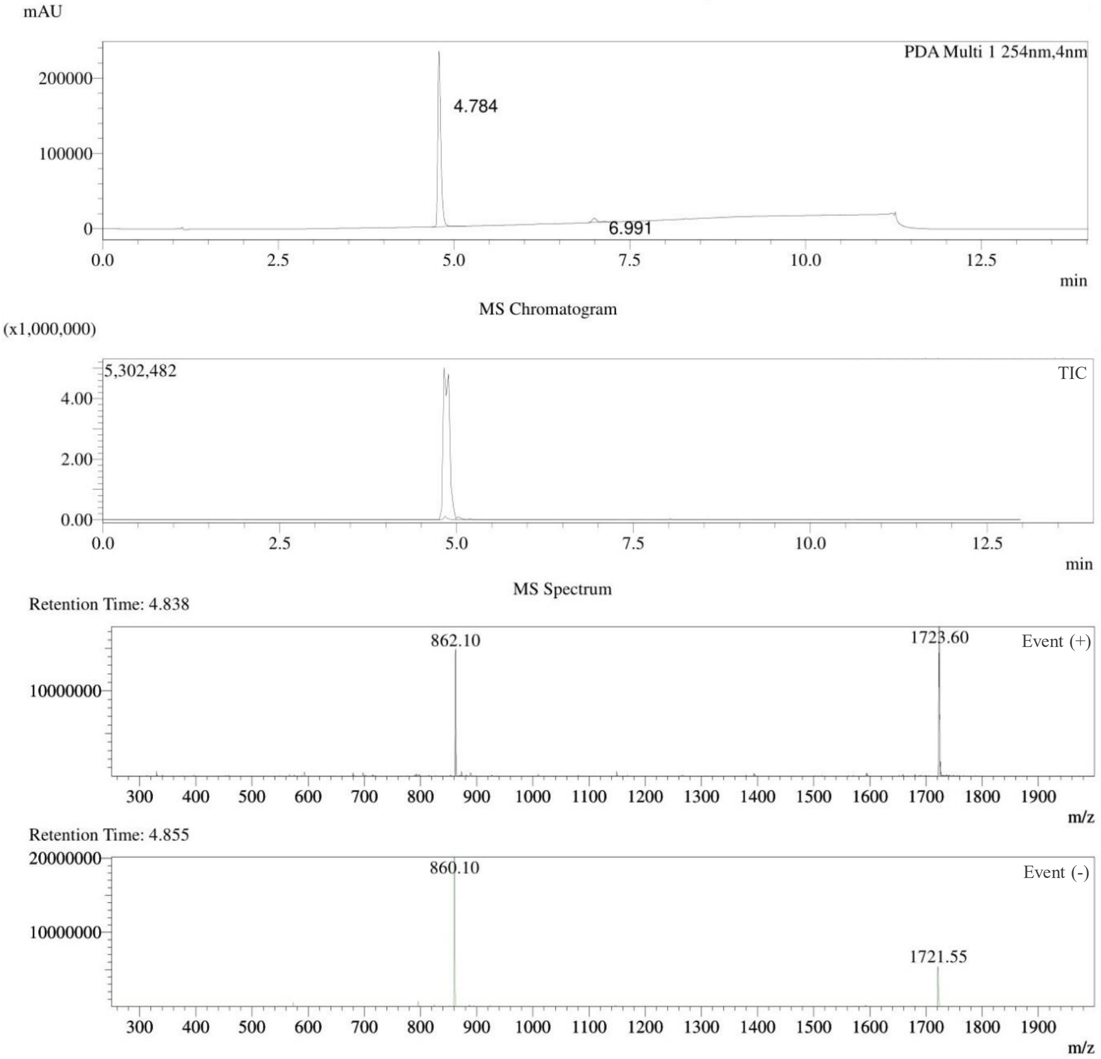

**N-terminal free GA5**

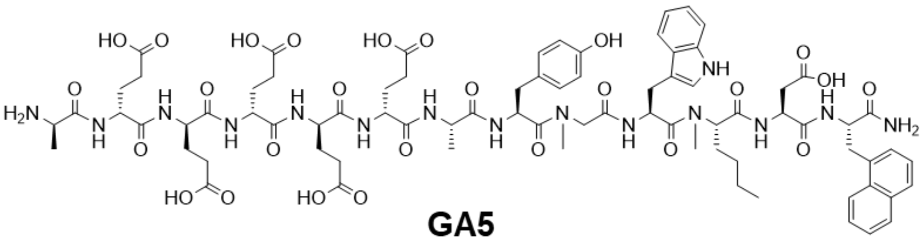

**GA5**

Exact Mass: 1663.70
Molecular Weight: 1664.75

SHIMADZU LabSolutions **Analysis Report**

**<Sample Information>**

| | |
|---|---|
| Sample Name | : GA5 |
| Method Filename | : 15to90ov7min_LCMS_210325.lcm |
| Batch Filename | : 20210329.lcb |
| Injection Volume | : 10 uL |
| Date Acquired | : 29/03/2021 1:15:24 PM |
| Date Processed | : 29/03/2021 1:29:28 PM |
| Acquired by | : Marwa Rahimi |
| Processed by | : Marwa Rahimi |

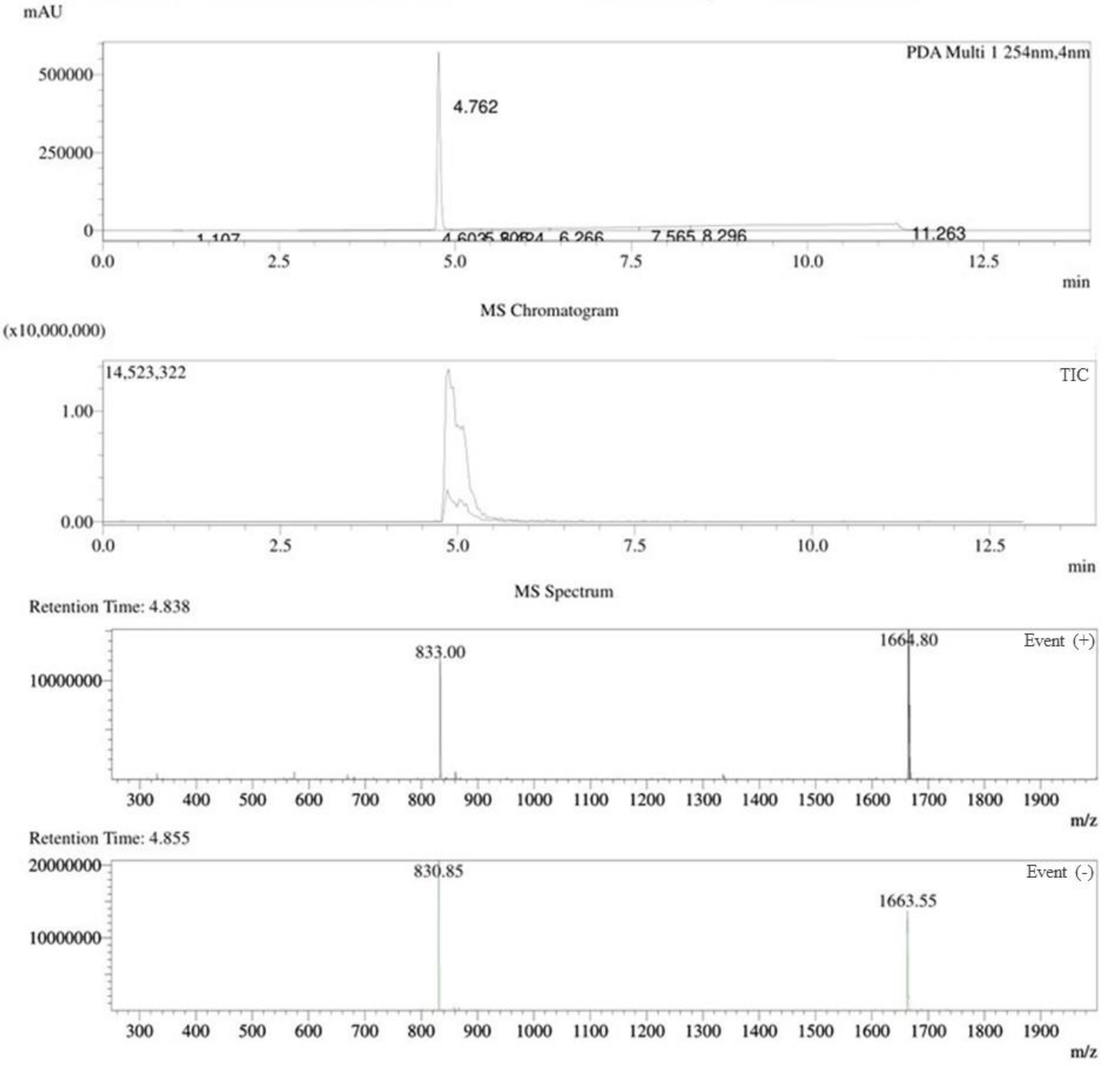

**N-terminal free GA6**

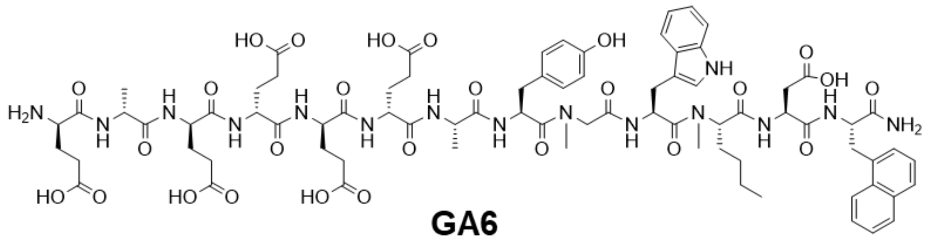

**GA6**

Exact Mass: 1663.70
Molecular Weight: 1664.75

SHIMADZU LabSolutions **Analysis Report**

**<Sample Information>**

| | |
|---|---|
| Sample Name | : GA6 |
| Method Filename | : 15to90ov7min_LCMS_210325.lcm |
| Batch Filename | : 20210329.lcb |
| Injection Volume | : 10 uL |
| Date Acquired | : 29/03/2021 1:30:05 PM |
| Date Processed | : 29/03/2021 1:44:07 PM |
| Acquired by | : Marwa Rahimi |
| Processed by | : Marwa Rahimi |

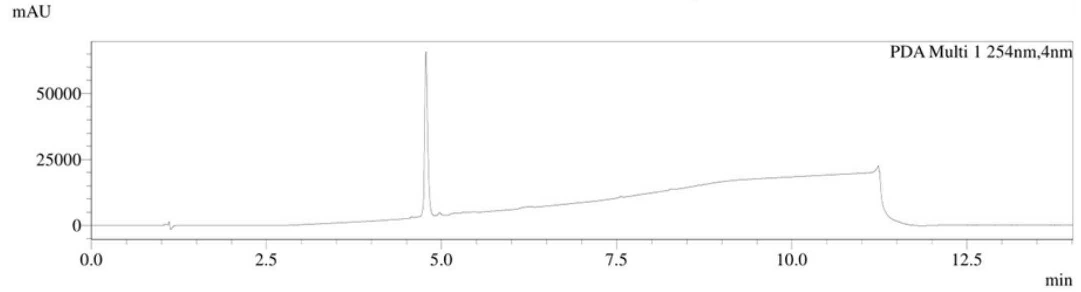


MS Chromatogram

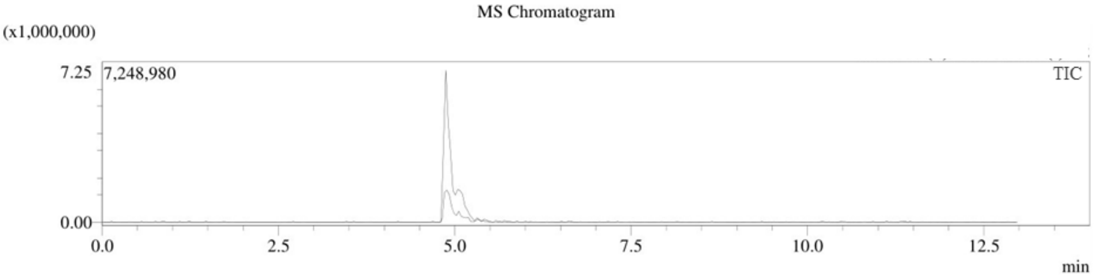


MS Spectrum

Retention Time: 4.872

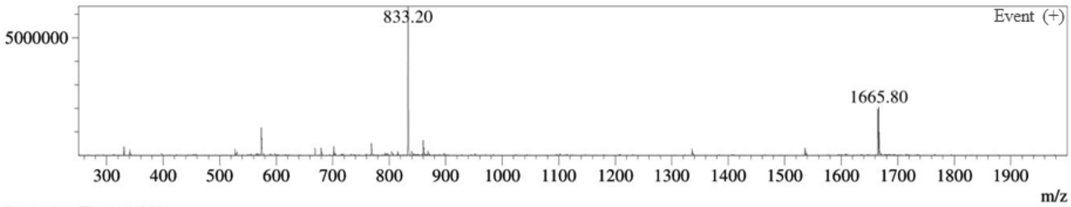


Retention Time: 4.889

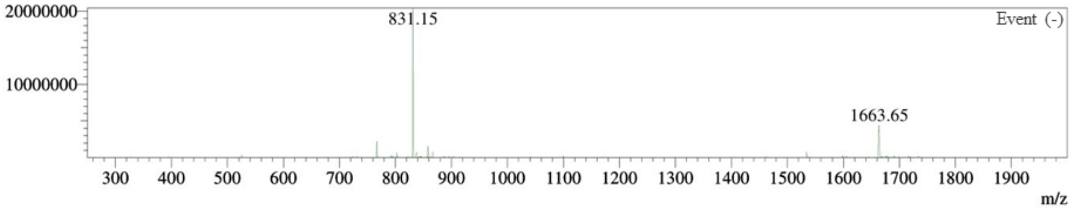

**N-terminal free GA7**

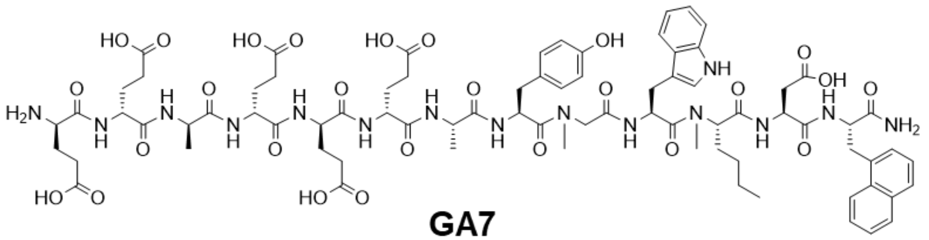


**GA7**

Exact Mass: 1663.70
Molecular Weight: 1664.75

SHIMADZU LabSolutions **Analysis Report**

**<Sample Information>**

Sample Name : GA7
Method Filename : 15to90ov7min_LCMS_210325.lcm
Batch Filename : 20210415.lcb
Injection Volume : 10 uL
Date Acquired : 15/04/2021 11:35:13 AM    Acquired by : Marwa Rahimi
Date Processed : 15/04/2021 11:49:16 AM    Processed by : Marwa Rahimi

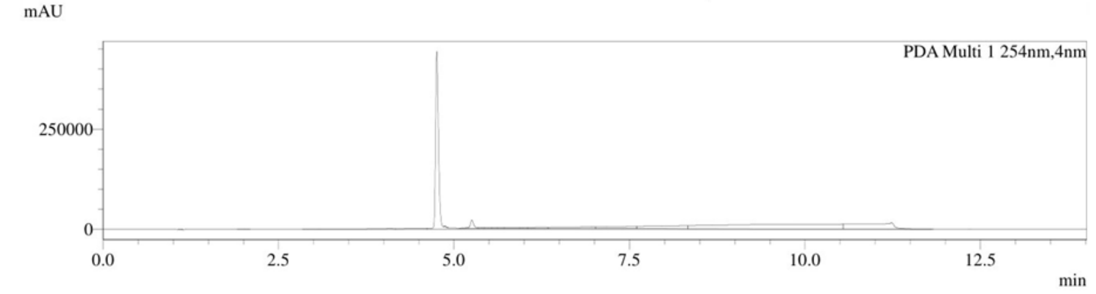


MS Chromatogram

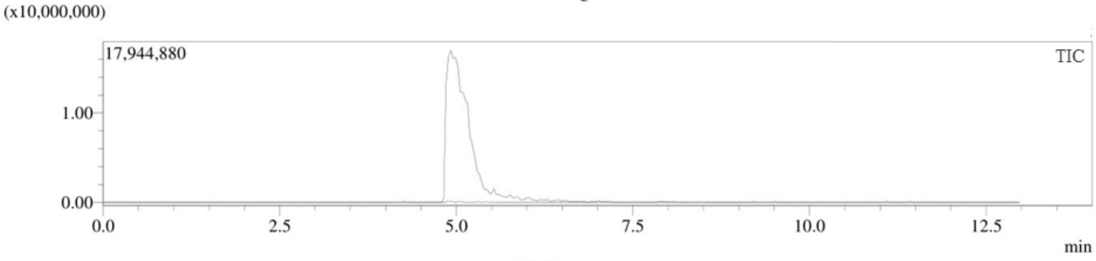


MS Spectrum

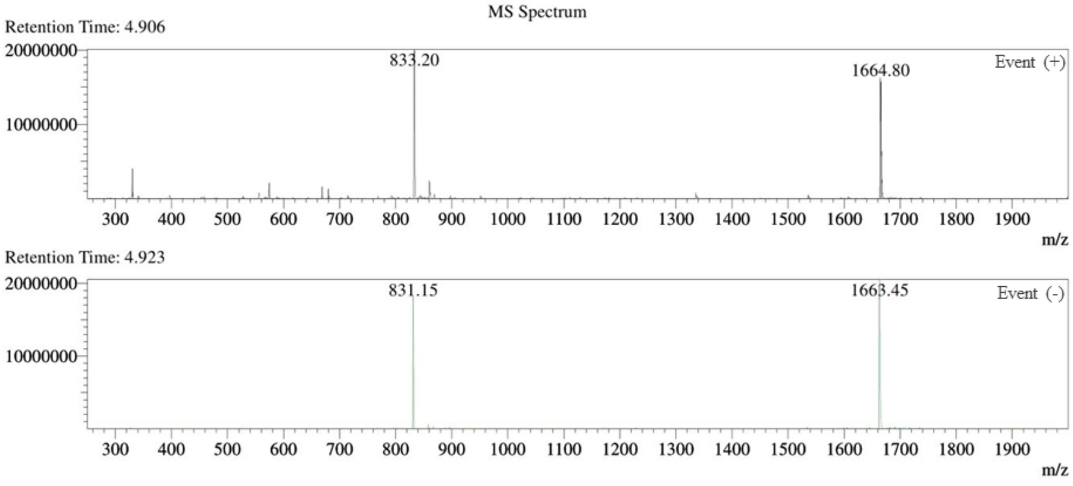

**N-terminal free GA8**

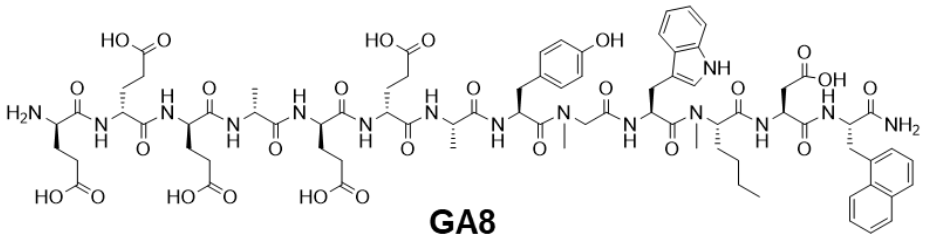

**GA8**

Exact Mass: 1663.70
Molecular Weight: 1664.75

SHIMADZU LabSolutions **Analysis Report**

**<Sample Information>**

| | | | |
|---|---|---|---|
| Sample Name | : GA8 | | |
| Method Filename | : 15to90ov7min_LCMS_210325.lcm | | |
| Batch Filename | : 20210329.lcb | | |
| Injection Volume | : 10 uL | | |
| Date Acquired | : 29/03/2021 2:11:54 PM | Acquired by | : Marwa Rahimi |
| Date Processed | : 29/03/2021 2:25:57 PM | Processed by | : Marwa Rahimi |

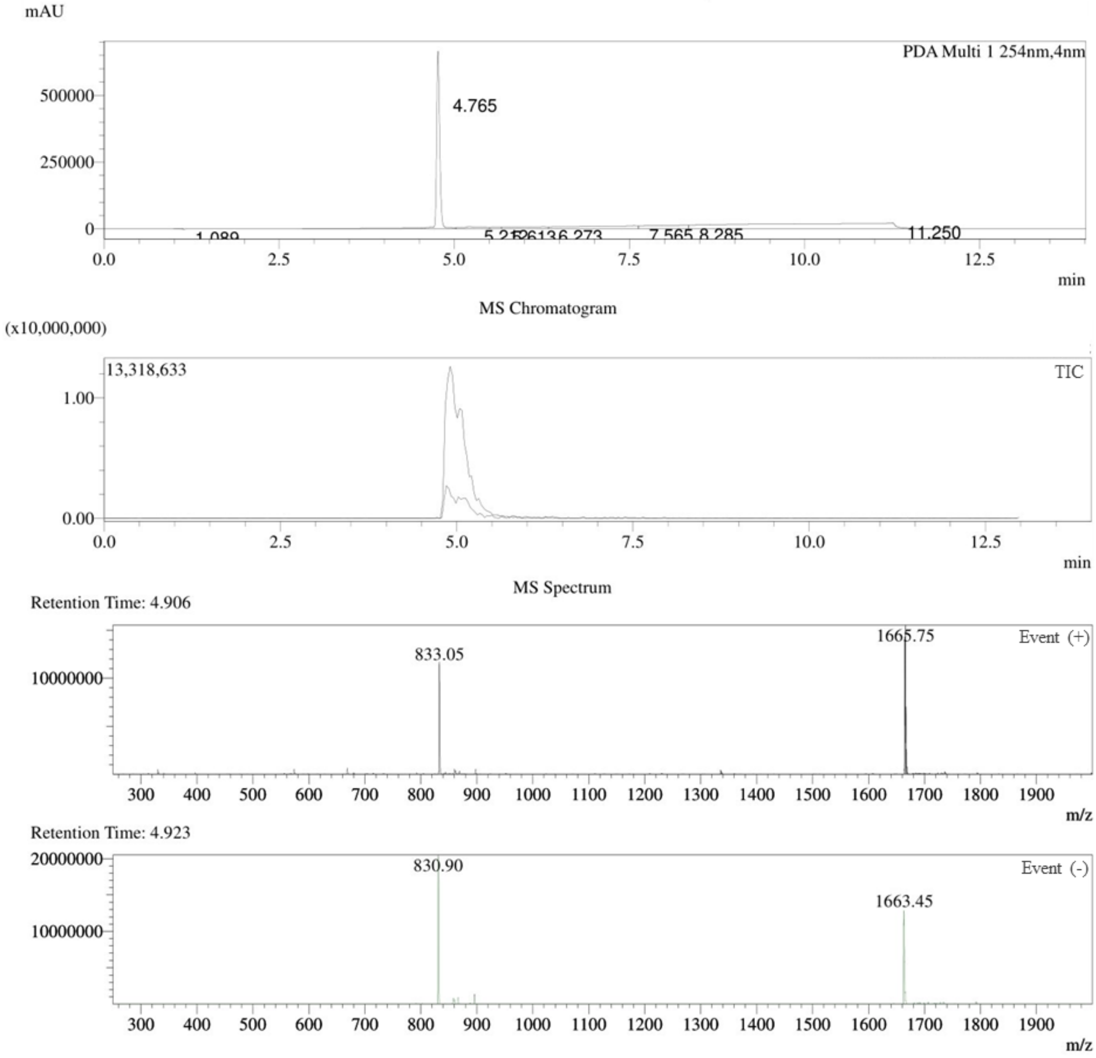

**N-terminal free GA9**

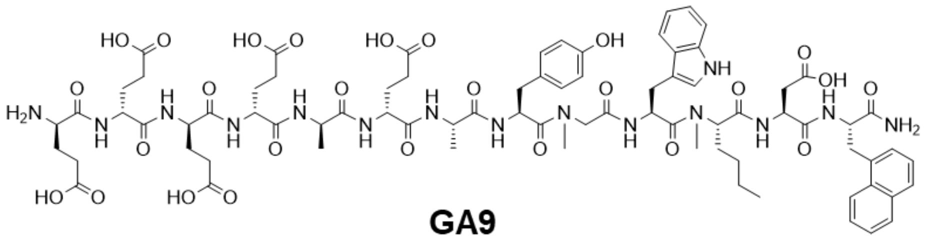

GA9

Exact Mass: 1663.70
Molecular Weight: 1664.75

SHIMADZU LabSolutions **Analysis Report**

**<Sample Information>**

| | | | |
|---|---|---|---|
| Sample Name | : GA9 | | |
| Method Filename | : 15to90ov7min_LCMS_210325.lcm | | |
| Batch Filename | : 20210329.lcb | | |
| Injection Volume | : 10 uL | | |
| Date Acquired | : 29/03/2021 2:41:16 PM | Acquired by | : Marwa Rahimi |
| Date Processed | : 29/03/2021 2:55:20 PM | Processed by | : Marwa Rahimi |

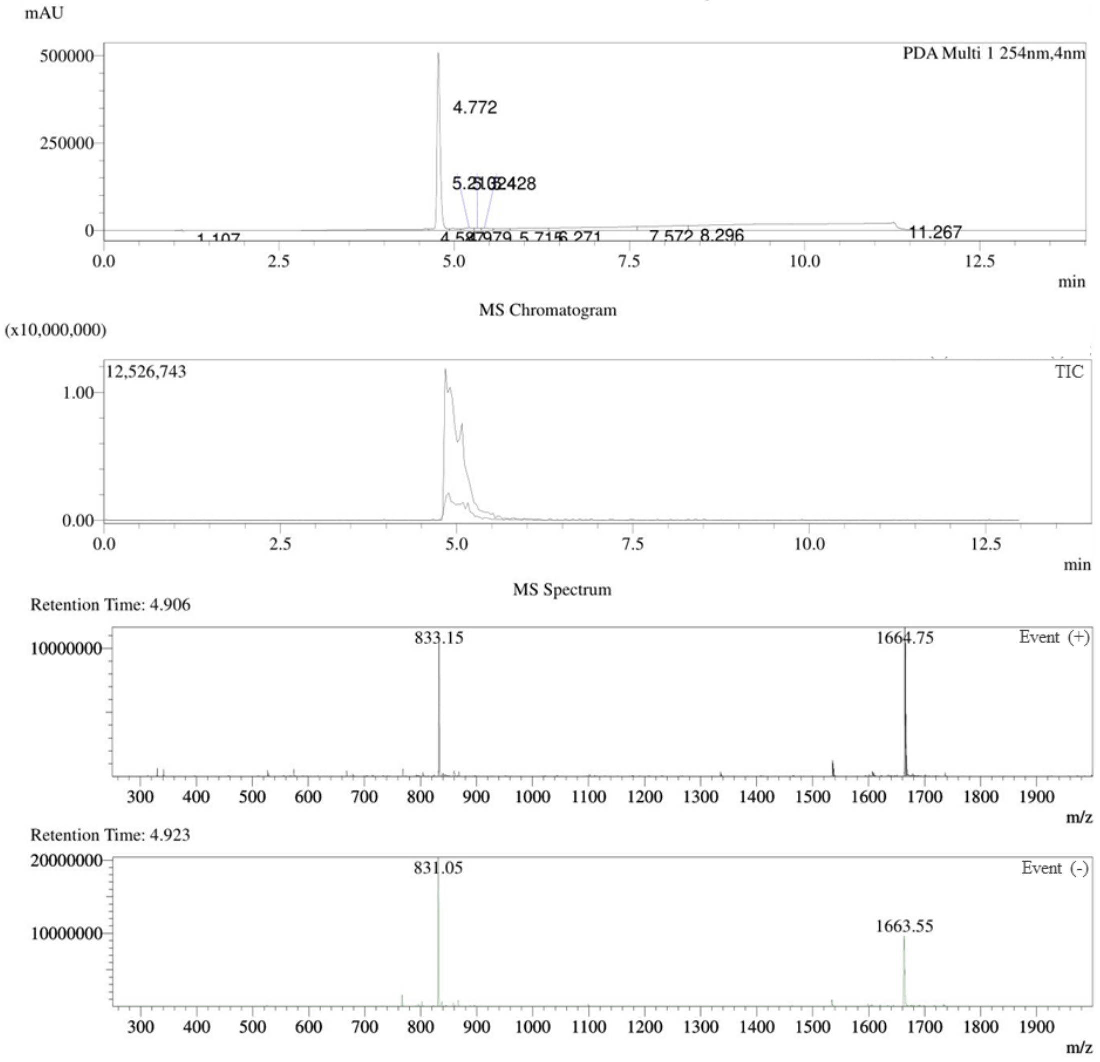

**N-terminal free GA10**

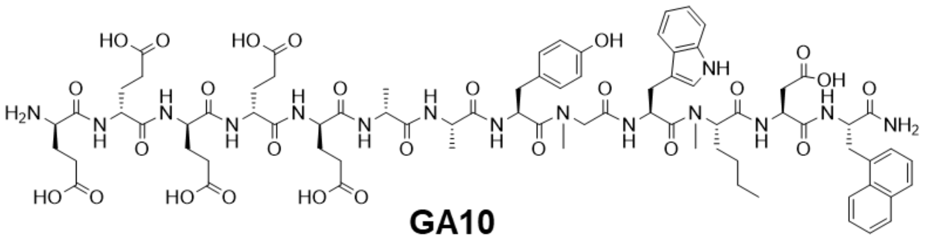

**GA10**

Exact Mass: 1663.70
Molecular Weight: 1664.75

SHIMADZU LabSolutions

# Analysis Report

**<Sample Information>**

Sample Name : GA10
Method Filename : 15to90ov7min_LCMS_210325.lcm
Batch Filename : 20210329.lcb
Injection Volume : 10 uL
Date Acquired : 29/03/2021 3:40:03 PM   Acquired by : Marwa Rahimi
Date Processed : 29/03/2021 3:54:06 PM   Processed by : Marwa Rahimi

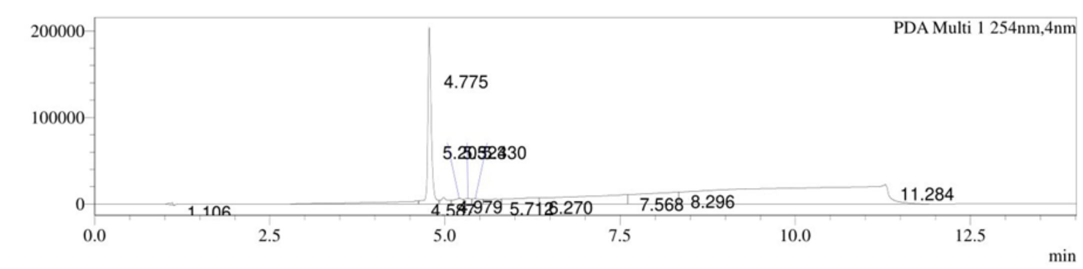


MS Chromatogram

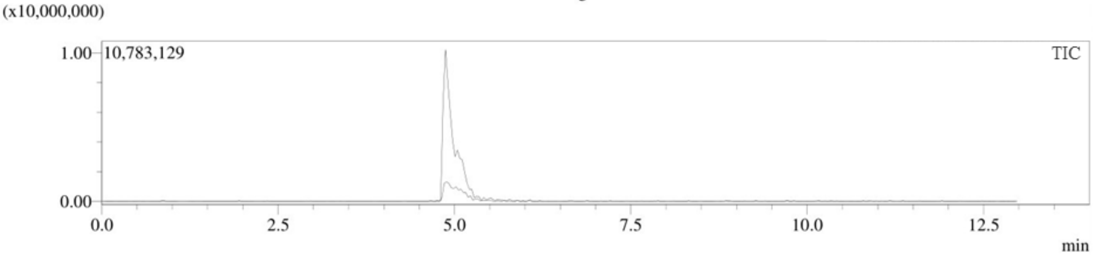


MS Spectrum

Retention Time: 4.838

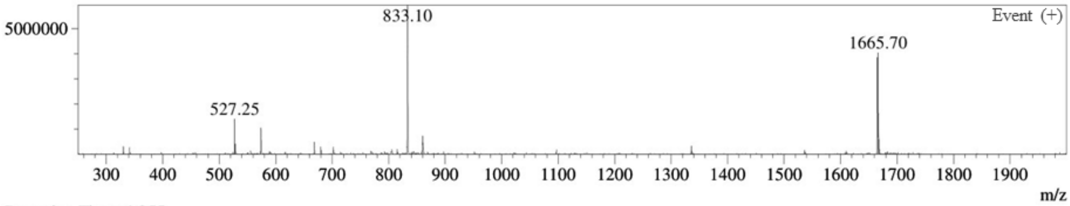


Retention Time: 4.855

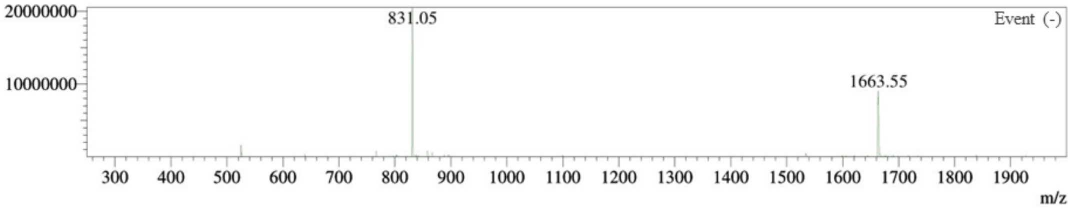

**N-terminal free GA4**

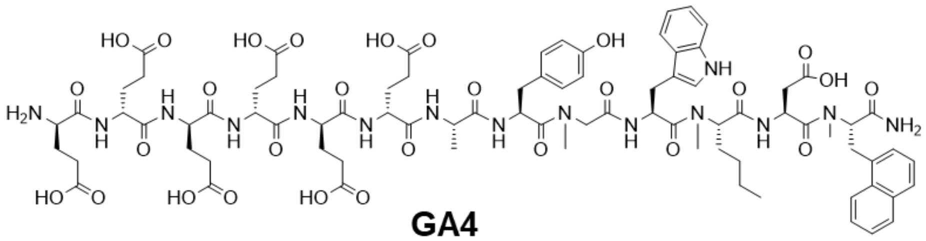

**GA4**

Exact Mass: 1735.73
Molecular Weight: 1736.81

SHIMADZU LabSolutions **Analysis Report**

**<Sample Information>**

Sample Name : GA4
Method Filename : 15to90ov7min_LCMS_210325.lcm
Batch Filename : 20210415.lcb
Injection Volume : 10 uL
Date Acquired : 15/04/2021 1:17:04 PM   Acquired by : Marwa Rahimi
Date Processed : 15/04/2021 1:31:08 PM   Processed by : Marwa Rahimi

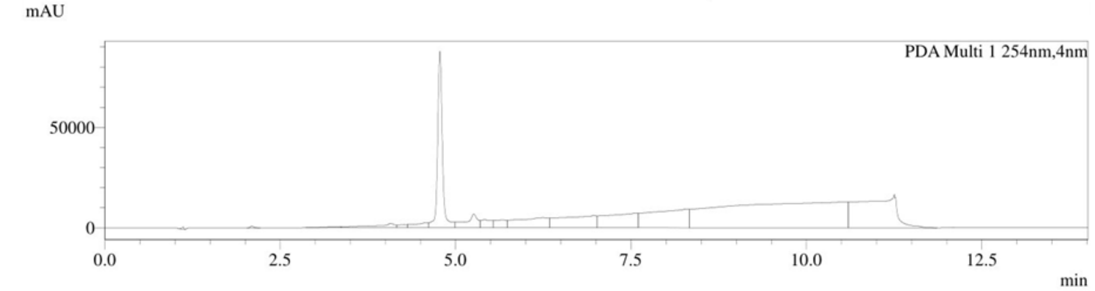


MS Chromatogram

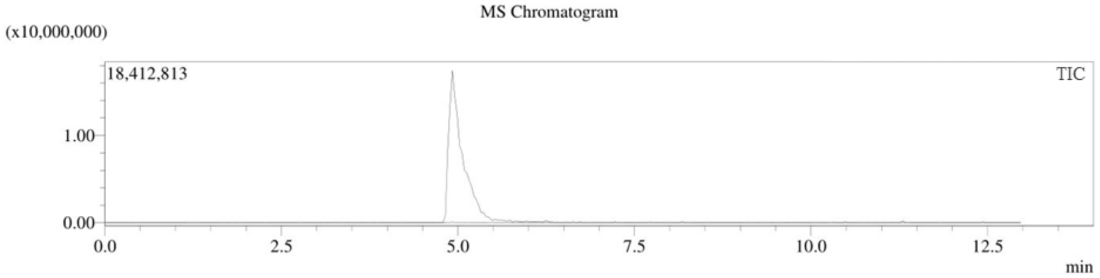


MS Spectrum

Retention Time: 4.906

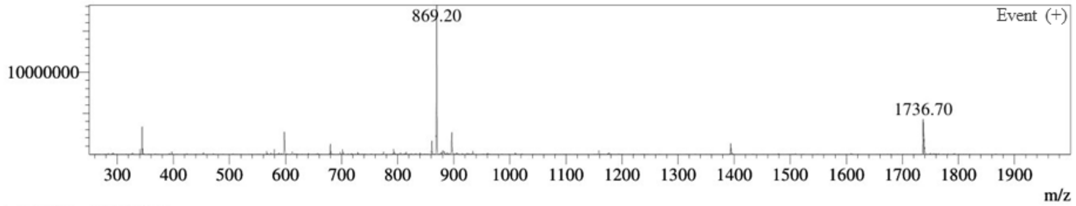


Retention Time: 4.923

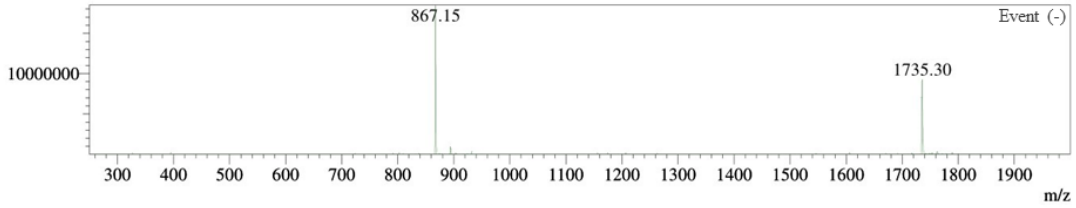

**N-terminal free GA11**

**GA11**

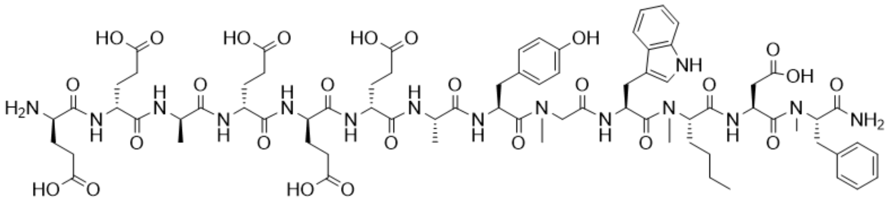


Exact Mass: 1627.70
Molecular Weight: 1628.71

SHIMADZU LabSolutions

# Analysis Report

**<Sample Information>**

Sample Name : GA11
Method Filename : 15to90ov7min_LCMS_210325.lcm
Batch Filename : 20210415.lcb
Injection Volume : 10 uL
Date Acquired : 15/04/2021 11:49:53 AM Acquired by : Marwa Rahimi
Date Processed : 15/04/2021 12:03:58 PM Processed by : Marwa Rahimi

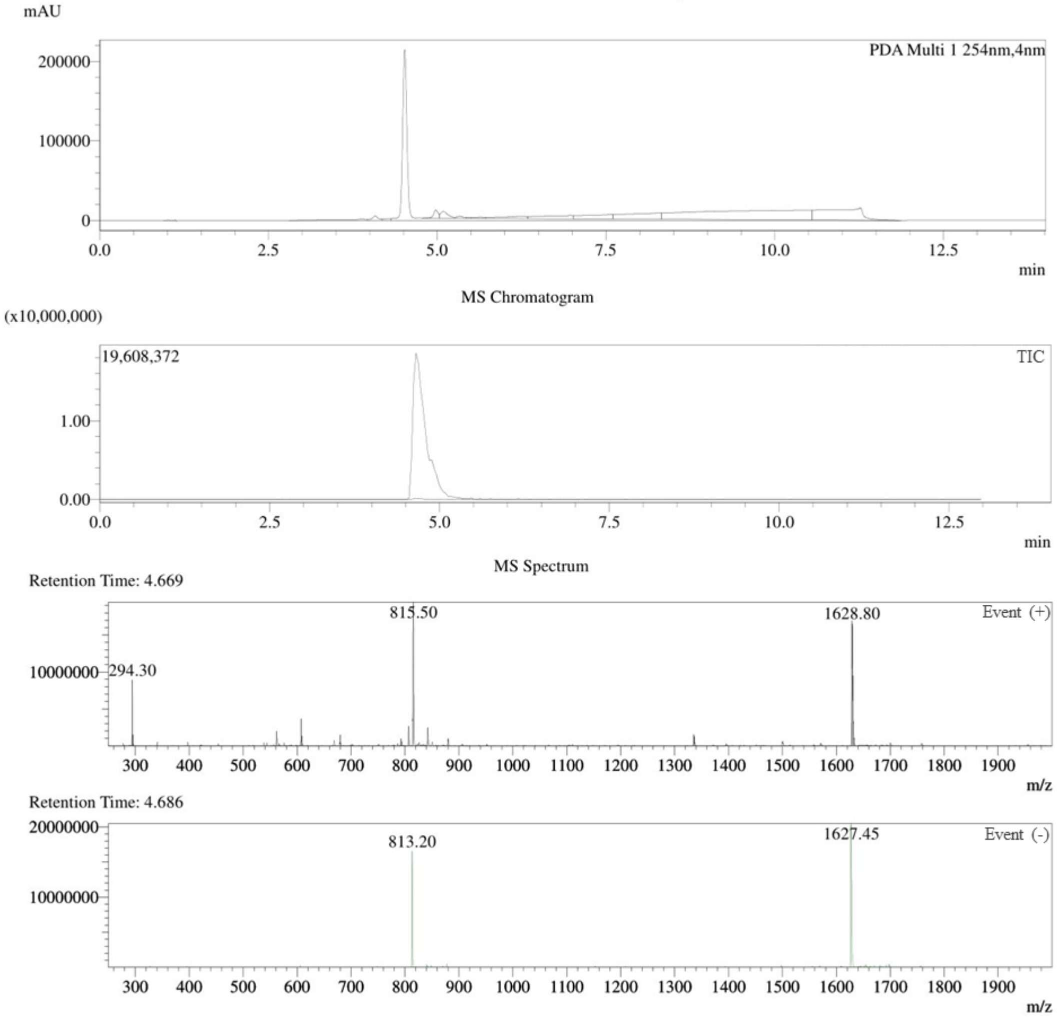

**N-terminal free GA12**

**GA12**

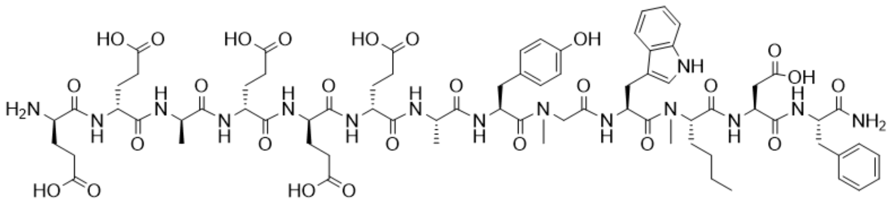

Exact Mass: 1613.69
Molecular Weight: 1614.69

SHIMADZU LabSolutions **Analysis Report**

**<Sample Information>**

Sample Name : GA12
Method Filename : 15to90ov7min_LCMS_210325.lcm
Batch Filename : 20210415.lcb
Injection Volume : 10 uL
Date Acquired : 15/04/2021 12:04:34 PM Acquired by : Marwa Rahimi
Date Processed : 15/04/2021 12:18:37 PM Processed by : Marwa Rahimi

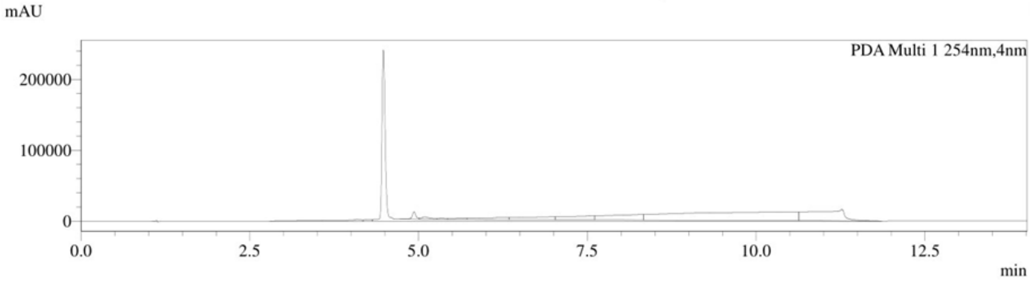


MS Chromatogram

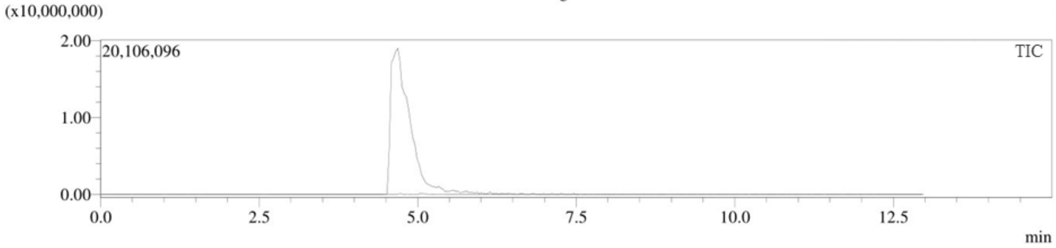


MS Spectrum

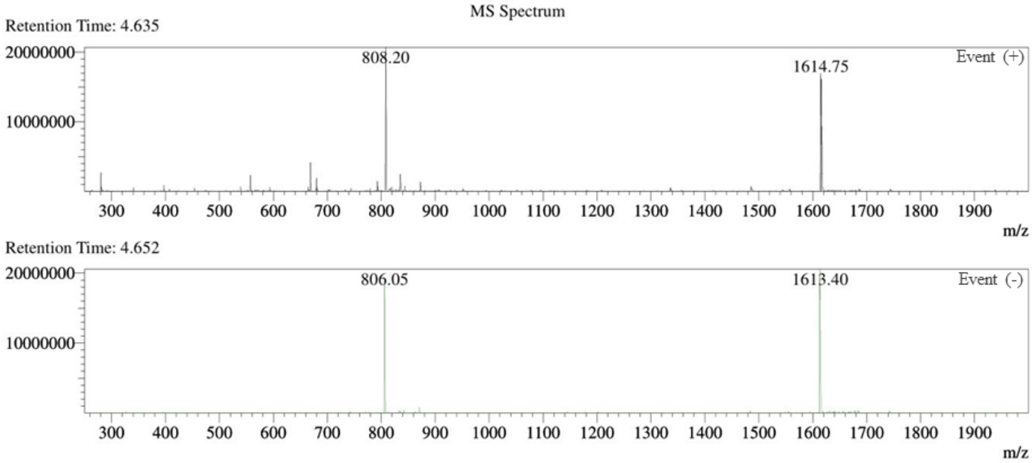

**N-terminal free GA13**

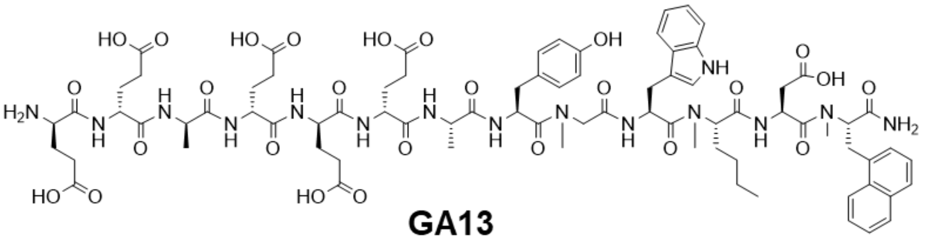

**GA13**

Exact Mass: 1677.72
Molecular Weight: 1678.77

SHIMADZU LabSolutions **Analysis Report**

**<Sample Information>**

Sample Name : GA13
Method Filename : 15to90ov7min_LCMS_210325.lcm
Batch Filename : 20210415.lcb
Injection Volume : 10 uL
Date Acquired : 15/04/2021 12:19:14 PM    Acquired by : Marwa Rahimi
Date Processed : 15/04/2021 12:30:48 PM    Processed by : Marwa Rahimi

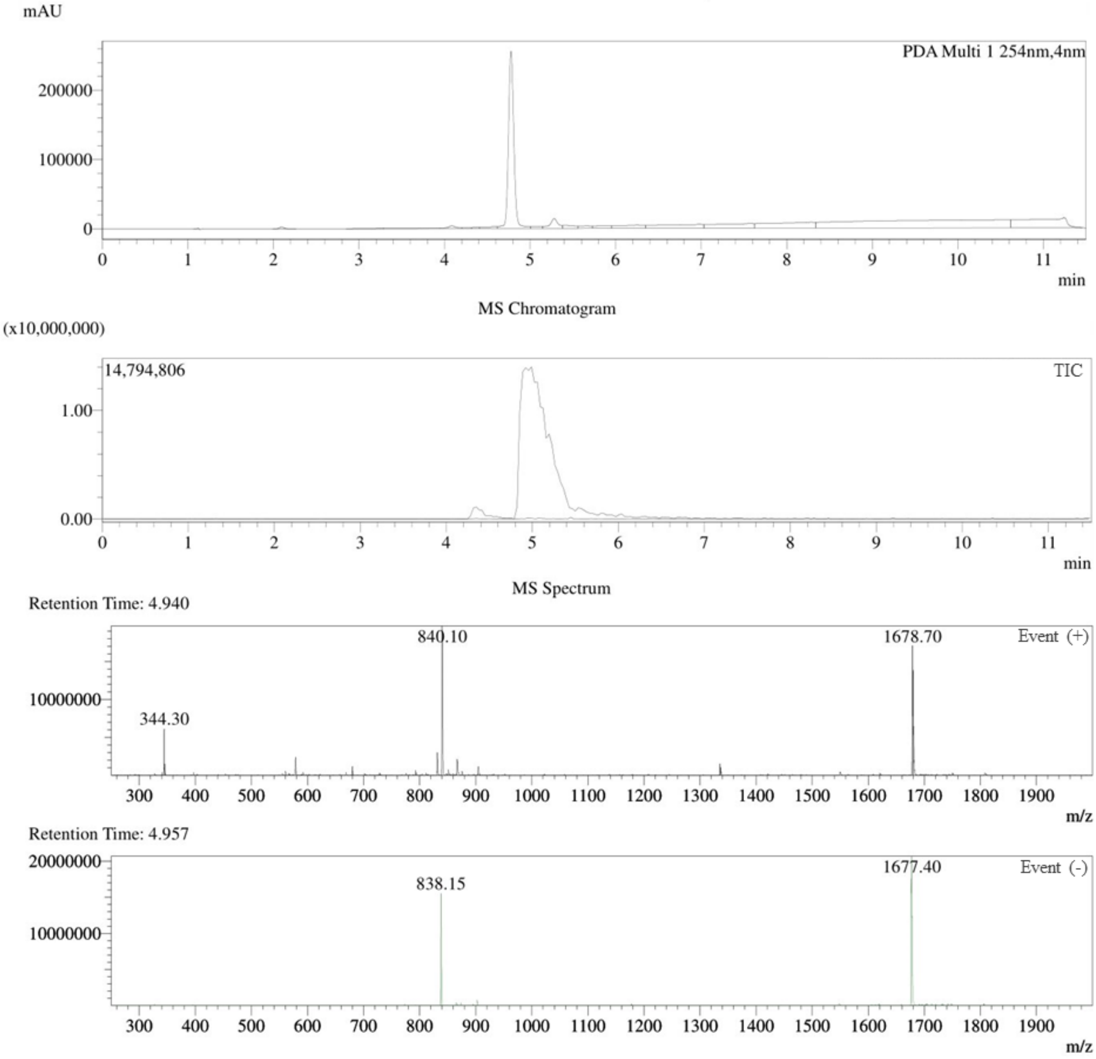

**DOTA-GA4**

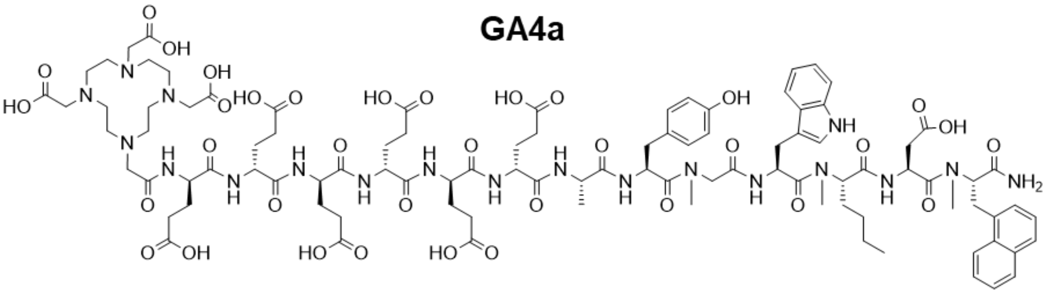


Exact Mass: 2121.91
Molecular Weight: 2123.21

SHIMADZU LabSolutions **Analysis Report**

**<Sample Information>**

| | |
|---|---|
| Sample Name | : DOTA-GA4 |
| Method Filename | : 15to90ov7min_LCMS_210325.lcm |
| Batch Filename | : 20210518.lcb |
| Injection Volume | : 10 uL |
| Date Acquired | : 18/05/2021 3:41:16 PM |
| Date Processed | : 20/05/2021 3:00:38 PM |
| Acquired by | : Marwa Rahimi |
| Processed by | : Marwa Rahimi |

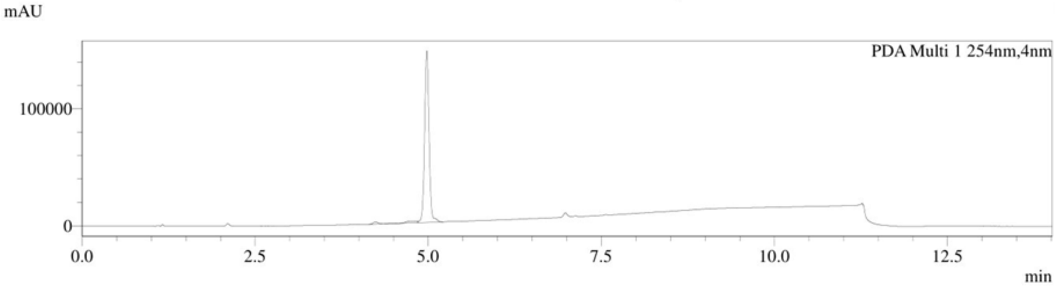


MS Chromatogram

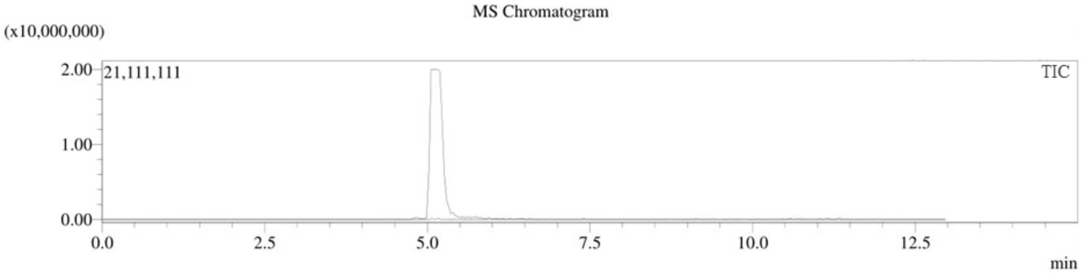


MS Spectrum

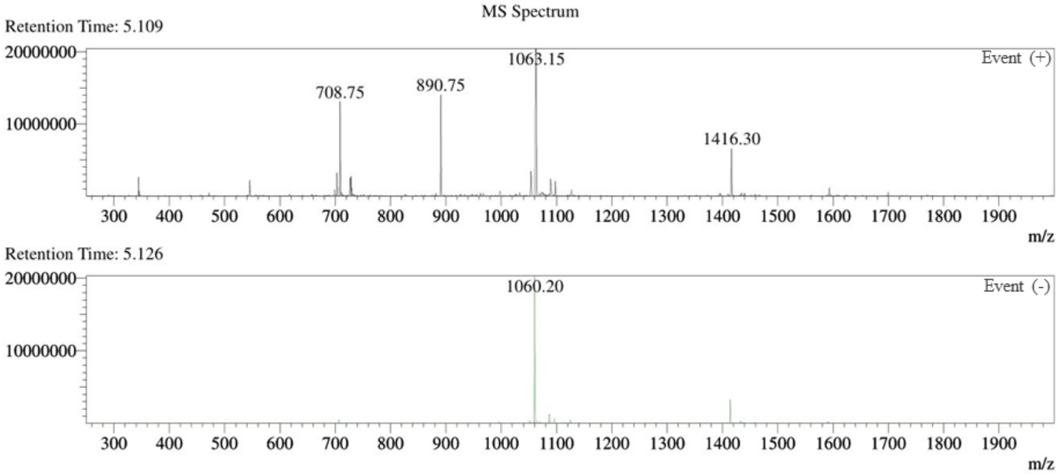

**DOTA-GA7**

GA7a

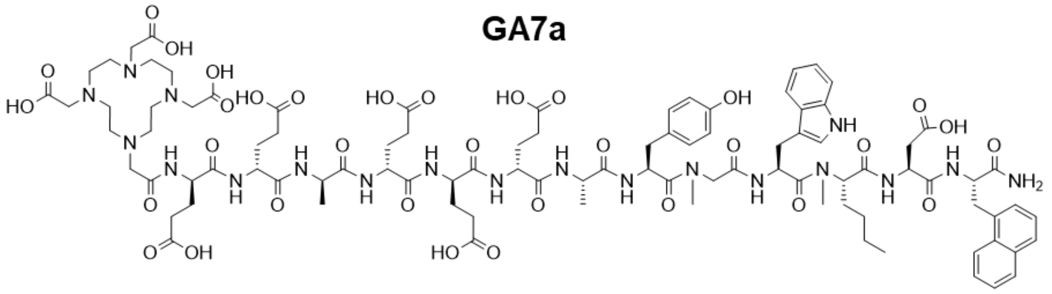

Exact Mass: 2049.88
Molecular Weight: 2051.15

SHIMADZU LabSolutions **Analysis Report**

<Sample Information>

| | | | |
|---|---|---|---|
| Sample Name | : DOTA-GA7 | | |
| Method Filename | : 15to90ov7min_LCMS_210325.lcm | | |
| Batch Filename | : 20210518.lcb | | |
| Injection Volume | : 10 uL | | |
| Date Acquired | : 18/05/2021 3:55:58 PM | Acquired by | : Marwa Rahimi |
| Date Processed | : 19/05/2021 6:52:32 AM | Processed by | : Marwa Rahimi |

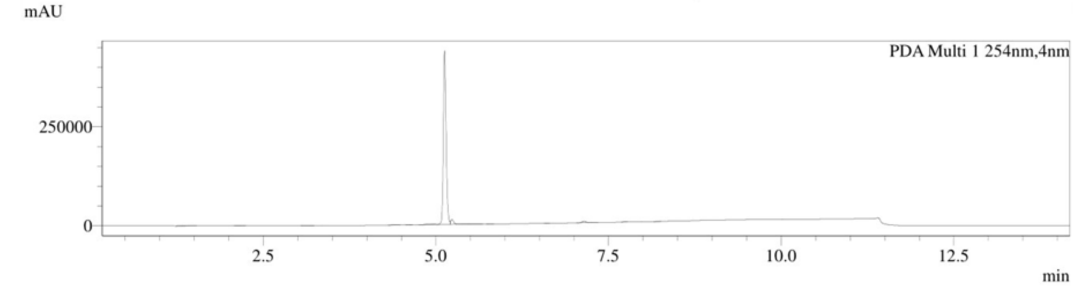


MS Chromatogram

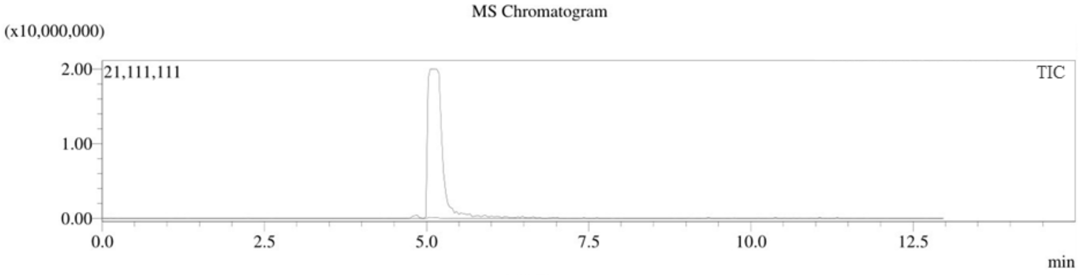


MS Spectrum

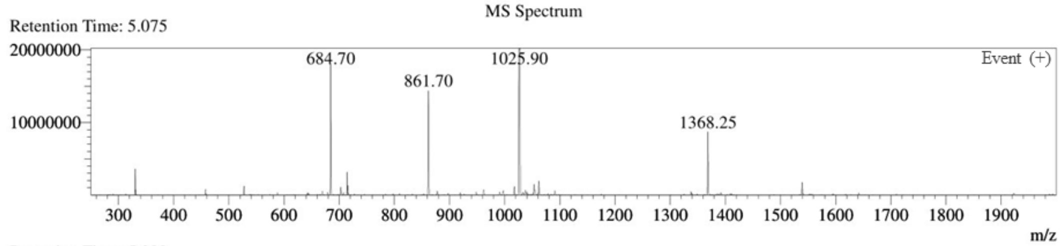


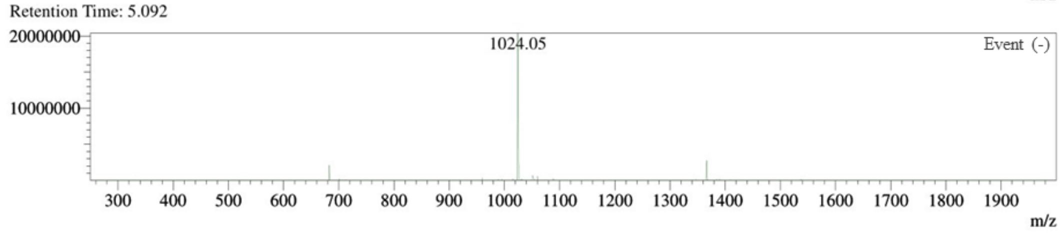

**DOTA-GA11**

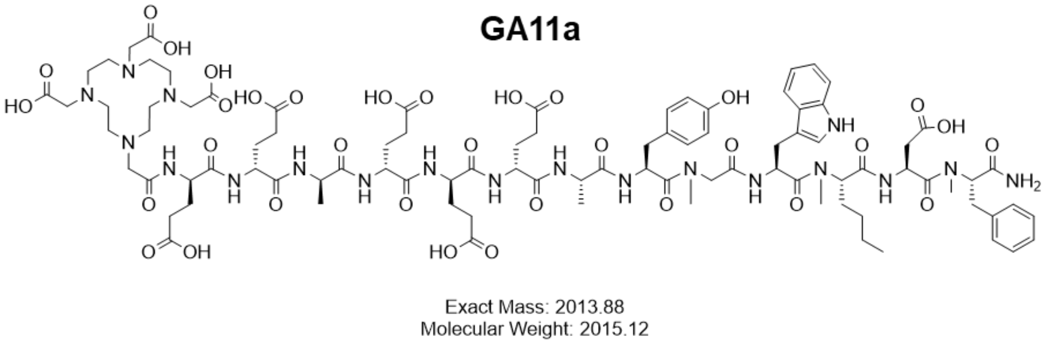


# Analysis Report

SHIMADZU LabSolutions

**<Sample Information>**

Sample Name : DOTA-GA11
Method Filename : 15to90ov7min_LCMS_210325.lcm
Batch Filename : 20210518.lcb
Injection Volume : 10 uL
Date Acquired : 18/05/2021 4:10:40 PM
Date Processed : 20/05/2021 3:00:57 PM
Acquired by : Marwa Rahimi
Processed by : Marwa Rahimi

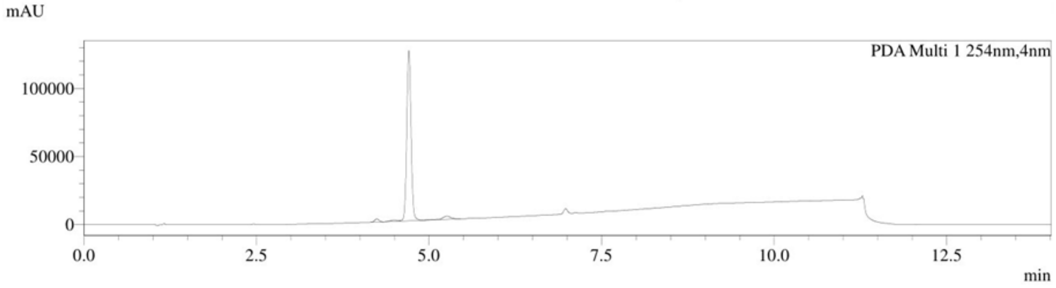


MS Chromatogram

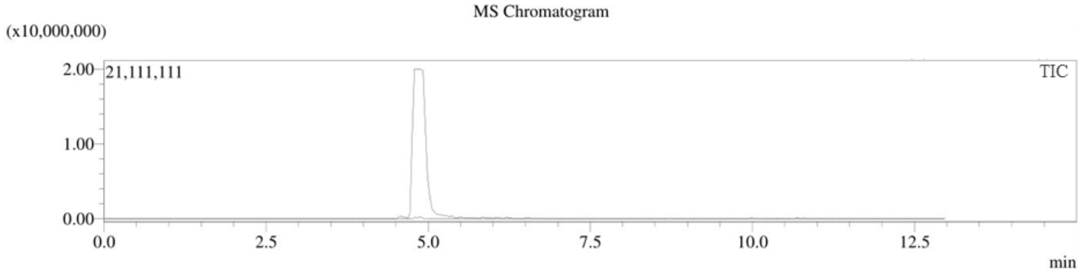


MS Spectrum

Retention Time: 4.771

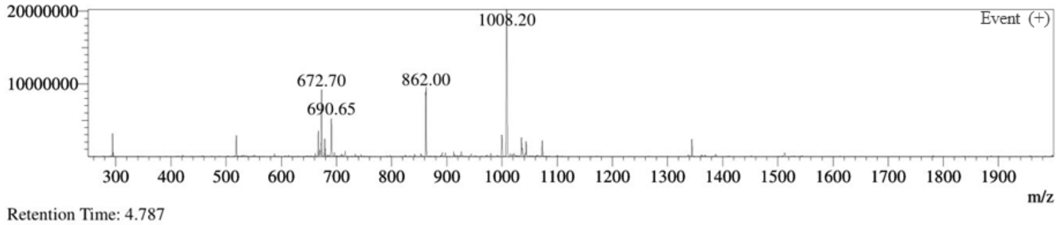


Retention Time: 4.787

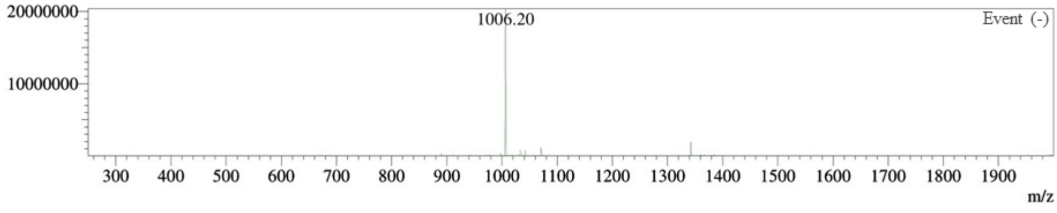

**DOTA-GA12**

GA12a

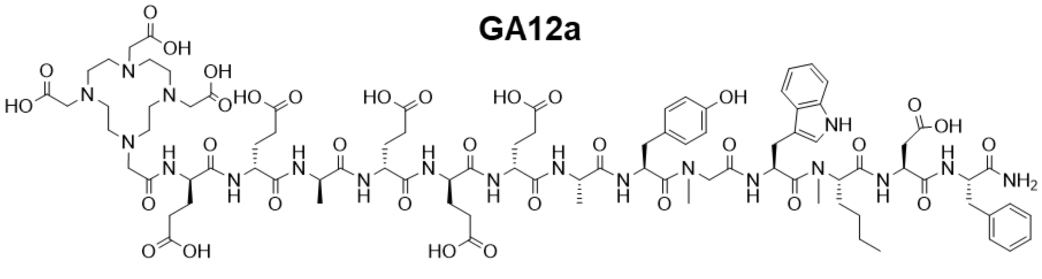


Exact Mass: 1999.87
Molecular Weight: 2001.09

SHIMADZU LabSolutions **Analysis Report**

**<Sample Information>**

| | | | |
|---|---|---|---|
| Sample Name | : DOTA-GA12 | | |
| Method Filename | : 15to90ov7min_LCMS_210325.lcm | | |
| Batch Filename | : 20210518.lcb | | |
| Injection Volume | : 10 uL | | |
| Date Acquired | : 18/05/2021 4:25:23 PM | Acquired by | : Marwa Rahimi |
| Date Processed | : 20/05/2021 3:01:35 PM | Processed by | : Marwa Rahimi |

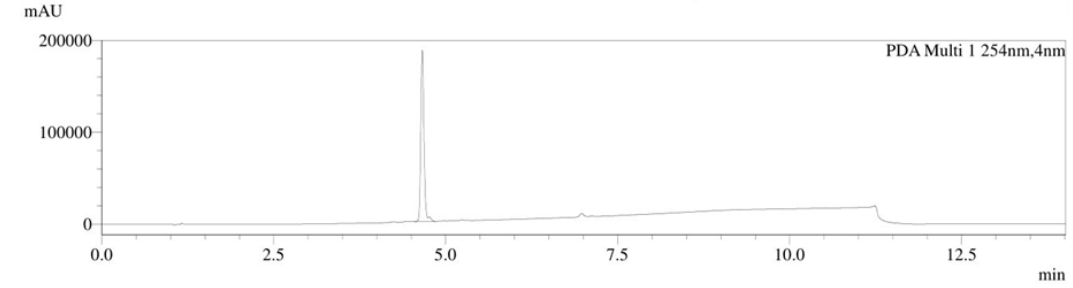


MS Chromatogram

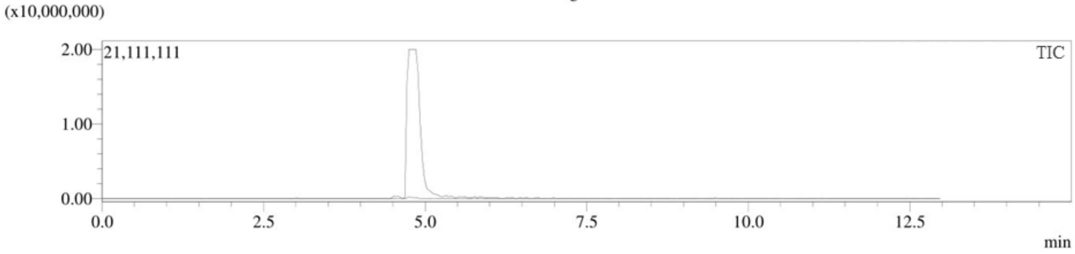


MS Spectrum

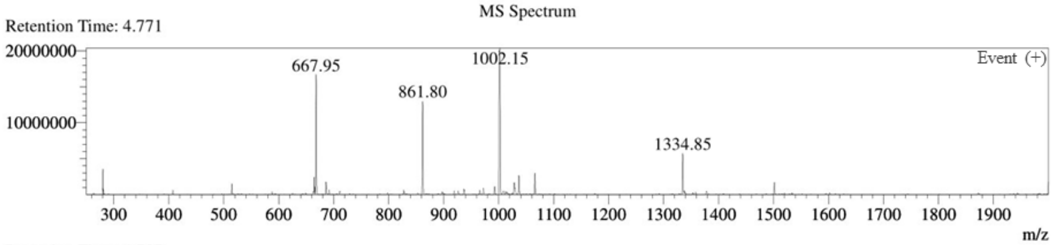


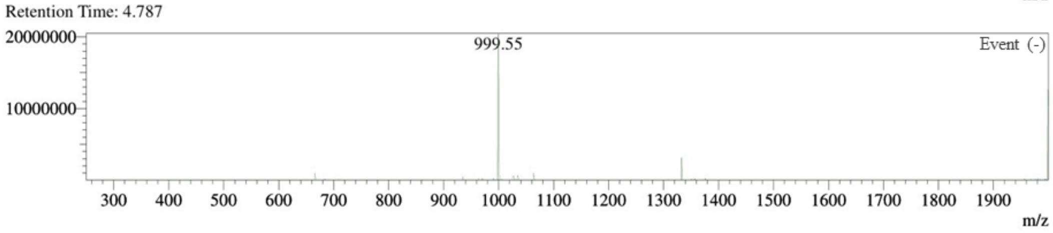

## DOTA-GA13

GA13a

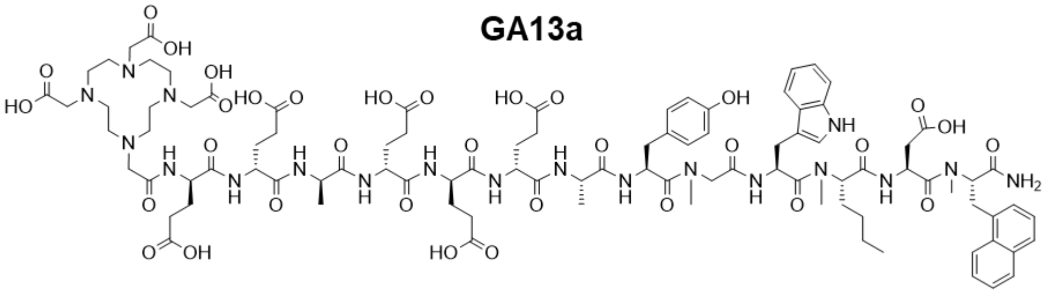

Exact Mass: 2063.90
Molecular Weight: 2065.18

SHIMADZU LabSolutions

# Analysis Report

**<Sample Information>**

| | | | |
|---|---|---|---|
| Sample Name | : DOTA-GA13 | | |
| Method Filename | : 15to90ov7min_LCMS_210325.lcm | | |
| Batch Filename | : 20210518.lcb | | |
| Injection Volume | : 10 uL | | |
| Date Acquired | : 18/05/2021 4:40:04 PM | Acquired by | : Marwa Rahimi |
| Date Processed | : 20/05/2021 3:00:00 PM | Processed by | : Marwa Rahimi |

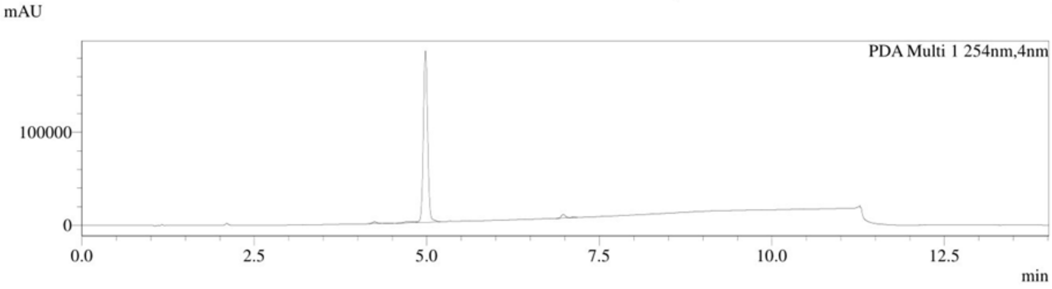


MS Chromatogram

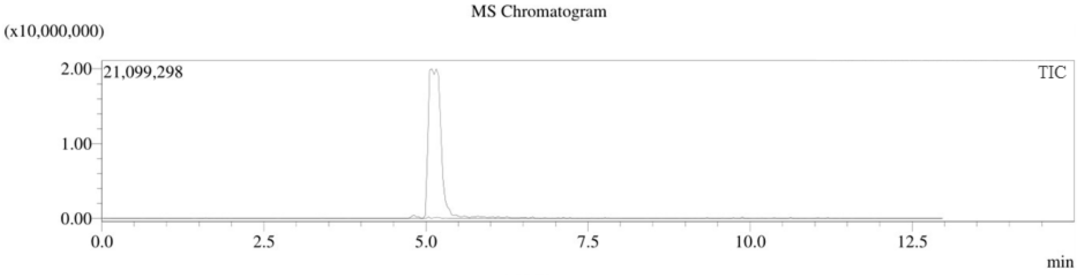


MS Spectrum

Retention Time: 5.143

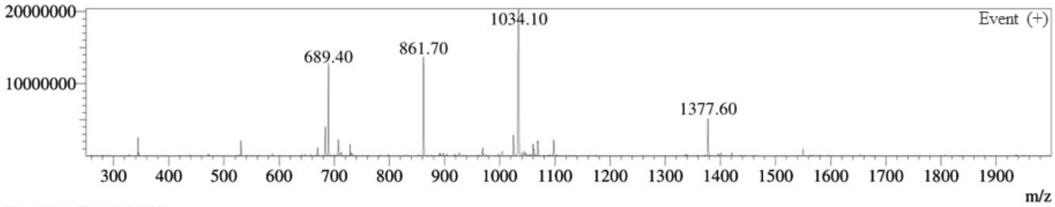


Retention Time: 5.160

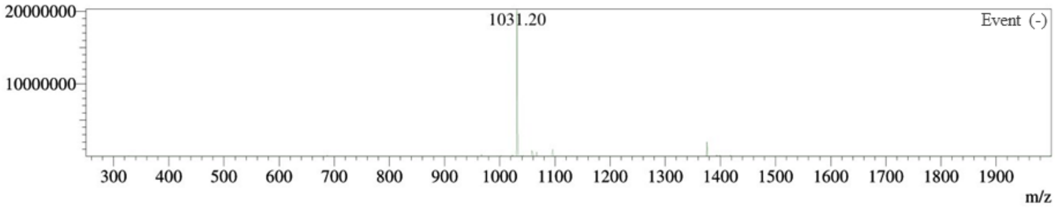

**Radiolabeled [$^{68}$Ga]Ga-DOTA-GA4**

**[$^{68}$Ga]Ga-GA4b**

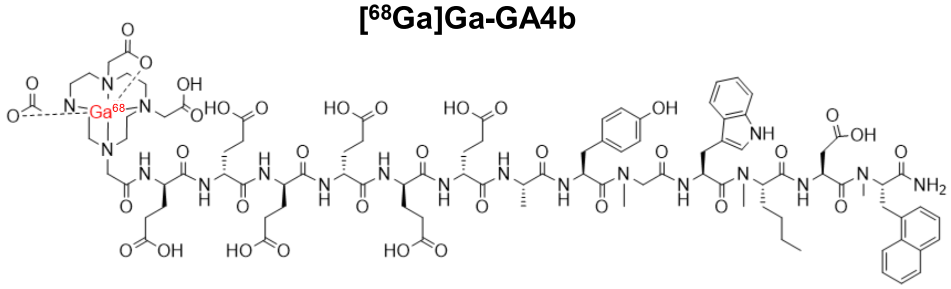

Exact Mass: 2188.82
Molecular Weight: 2190.92

SHIMADZU LabSolutions **Analysis Report**

**<Sample Information>**

Sample Name : Ga68-DOTA-GA4
Method Filename : 15to90ov7min_LCMS_QC.lcm
Batch Filename :
Injection Volume : 10 uL
Date Acquired : 20/05/2021 2:50:48 PM    Acquired by : Marwa Rahimi
Date Processed : 20/05/2021 3:04:51 PM    Processed by : Marwa Rahimi

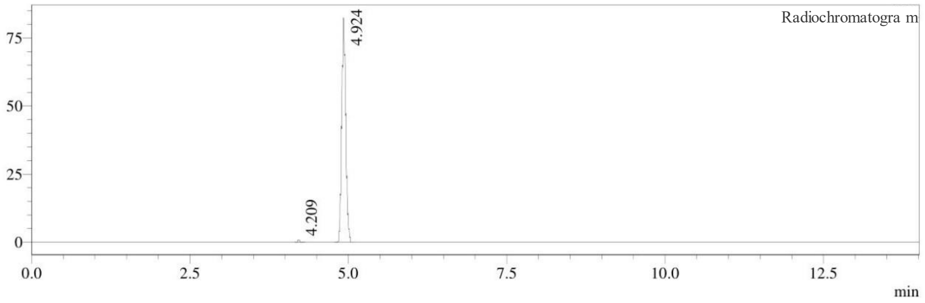


| Peak# | Ret. Time | Area | Area% |
|---|---|---|---|
| 1 | 4.209 | 1795 | 0.486 |
| 2 | 4.924 | 367501 | 99.514 |
| Total | | 369296 | 100 |

Radiochromatogram of [$^{68}$Ga]Ga-DOTA-**GA4**. Desired product obtained at >99% purity. Minor radioactive peak (<1%) appearing at retention time 4.209 min represents radiolysis product. Kinetex XB-C18 column (5 µm, 100 Å, 150 × 4.60 mm) eluted at 1.5 mL/min with a gradient of MeCN: 0.1% (w/v) formic acid, starting at 15% MeCN, increased to 50% B over 7 min and then increased to 90% and maintained at 90% MeCN for 3 min.

### Radiolabeled [$^{68}$Ga]Ga-DOTA-GA7

**[$^{68}$Ga]Ga-GA7b**

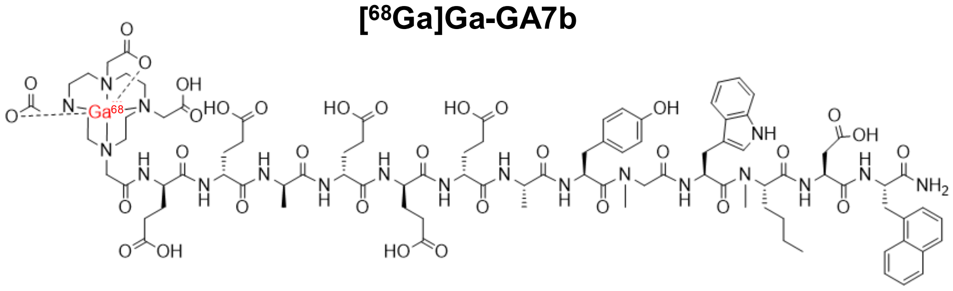

Exact Mass: 2116.79
Molecular Weight: 2118.86

**Analysis Report**

**<Sample Information>**

| | | | |
|---|---|---|---|
| Sample Name | : Ga68-DOTA-GA7 | | |
| Method Filename | : 15to90ov7min_LCMS_QC.lcm | | |
| Batch Filename | : 20210604.lcb | | |
| Injection Volume | : 10 uL | | |
| Date Acquired | : 4/06/2021 4:07:03 PM | Acquired by | : Marwa Rahimi |
| Date Processed | : 7/06/2021 11:57:24 AM | Processed by | : Marwa Rahimi |

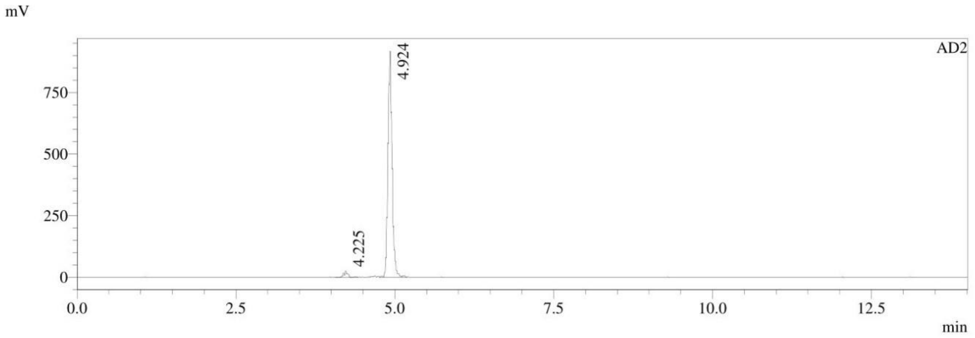


| Peak# | Ret. Time | Area | Area% |
|---|---|---|---|
| 1 | 4.21 | 67040 | 0.881 |
| 2 | 4.901 | 7545069 | 99.119 |
| Total | | 7612109 | 100 |

Radiochromatogram of [$^{68}$Ga]Ga-DOTA-**GA7**. Desired product obtained at >99% purity at retention time of 4.21 min. Minor radioactive peak (<1%) appearing at retention time 4.21 min represents radiolysis product. Kinetex XB-C18 column (5 µm, 100 Å, 150 × 4.60 mm) eluted at 1.5 mL/min with a gradient of MeCN: 0.1% (w/v) formic acid, starting at 15% MeCN, increased to 50% B over 7 min and then increased to 90% and maintained at 90% MeCN for 3 min.

## Radiolabeled [$^{68}$Ga]Ga-DOTA-GA11

**[$^{68}$Ga]Ga-GA11b**

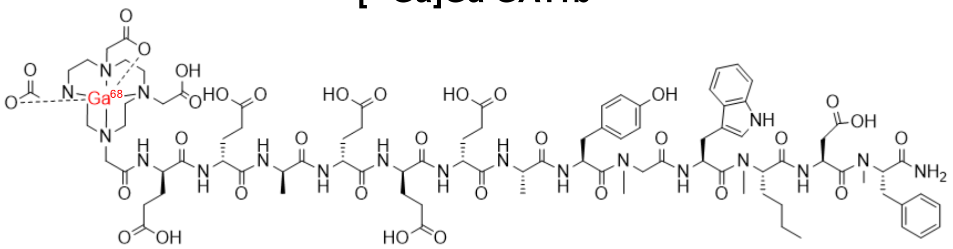

Exact Mass: 2080.79
Molecular Weight: 2082.82

SHIMADZU LabSolutions

# Analysis Report

**<Sample Information>**

Sample Name : Ga68-DOTA-GA11
Method Filename : 15to90ov7min_LCMS_QC.lcm
Batch Filename :
Injection Volume : 10 uL
Date Acquired : 4/06/2021 2:52:40 PM
Date Processed : 4/06/2021 3:34:15 PM
Acquired by : Marwa Rahimi
Processed by : Marwa Rahimi

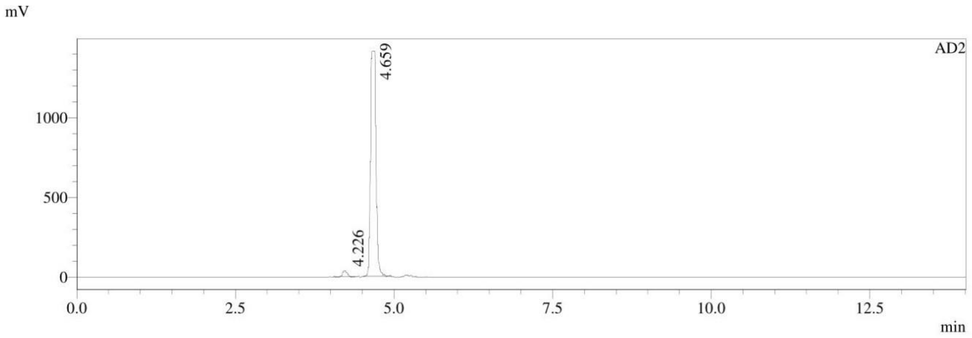


| Peak# | Ret. Time | Area | Area% |
|---|---|---|---|
| 1 | 4.226 | 171699 | 1.996 |
| 2 | 4.659 | 8431268 | 98.004 |
| Total | | 8602967 | 100 |

Radiochromatogram of [$^{68}$Ga]Ga-DOTA-**GA11**. Desired product obtained at >98% purity at retention time of 4.226 min. Minor radioactive peak (<2%) appearing at retention time 4.226 min represents radiolysis product. Kinetex XB-C18 column (5 µm, 100 Å, 150 × 4.60 mm) eluted at 1.5 mL/min with a gradient of MeCN: 0.1% (w/v) formic acid, starting at 15% MeCN, increased to 50% B over 7 min and then increased to 90% and maintained at 90% MeCN for 3 min.

## Radiolabeled [$^{68}$Ga]Ga-DOTA-GA12

**[$^{68}$Ga]Ga-GA12b**

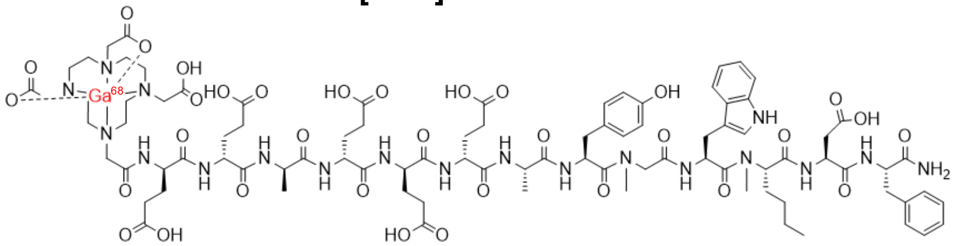

Exact Mass: 2066.78
Molecular Weight: 2068.80

**SHIMADZU LabSolutions Analysis Report**

**<Sample Information>**

Sample Name : Ga68-DOTA-GA12
Method Filename : 15to90ov7min_LCMS_QC.lcm
Batch Filename :
Injection Volume : 10 uL
Date Acquired : 4/06/2021 3:51:30 PM
Date Processed : 26/08/2023 12:38:48 PM
Acquired by : Marwa Rahimi
Processed by : Marwa Rahimi

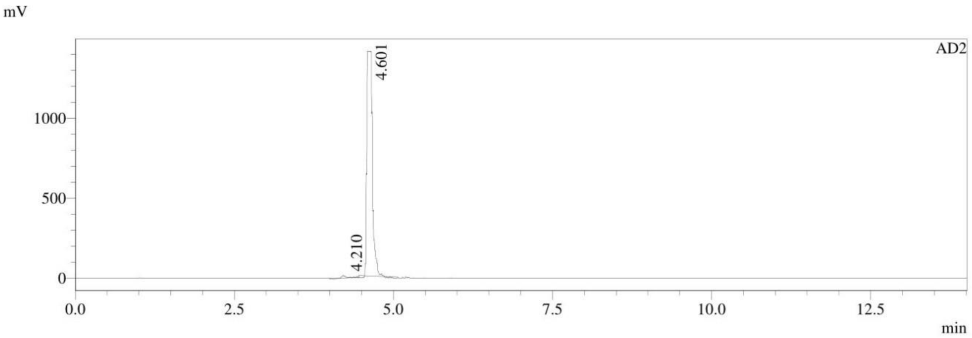


| Peak# | Ret. Time | Area | Area% |
|---|---|---|---|
| 1 | 4.21 | 106820 | 1.252 |
| 2 | 4.601 | 8425438 | 98.748 |
| Total | | 8532258 | 100 |

Radiochromatogram of [$^{68}$Ga]Ga-DOTA-**GA12**. Desired product obtained at >98% purity at retention time of 4.601 min. Minor radioactive peak (<2%) appearing at retention time 4.21 min represents radiolysis product. Kinetex XB-C18 column (5 µm, 100 Å, 150 × 4.60 mm) eluted at 1.5 mL/min with a gradient of MeCN: 0.1% (w/v) formic acid, starting at 15% MeCN, increased to 50% B over 7 min and then increased to 90% and maintained at 90% MeCN for 3 min.

**Radiolabeled [$^{68}$Ga]Ga-DOTA-GA13**

**[$^{68}$Ga]Ga-GA13b**

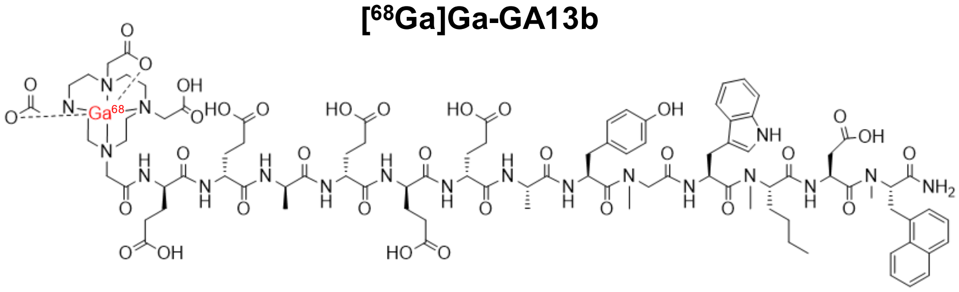

Exact Mass: 2130.81
Molecular Weight: 2132.88

SHIMADZU LabSolutions **Analysis Report**

**<Sample Information>**

Sample Name : DOTA-GA13b
Method Filename : 15to90ov7min_LCMS_QC.lcm
Batch Filename :
Injection Volume : 10 uL
Date Acquired : 4/06/2021 2:20:37 PM    Acquired by : Marwa Rahimi
Date Processed : 4/06/2021 4:03:30 PM    Processed by : Marwa Rahimi

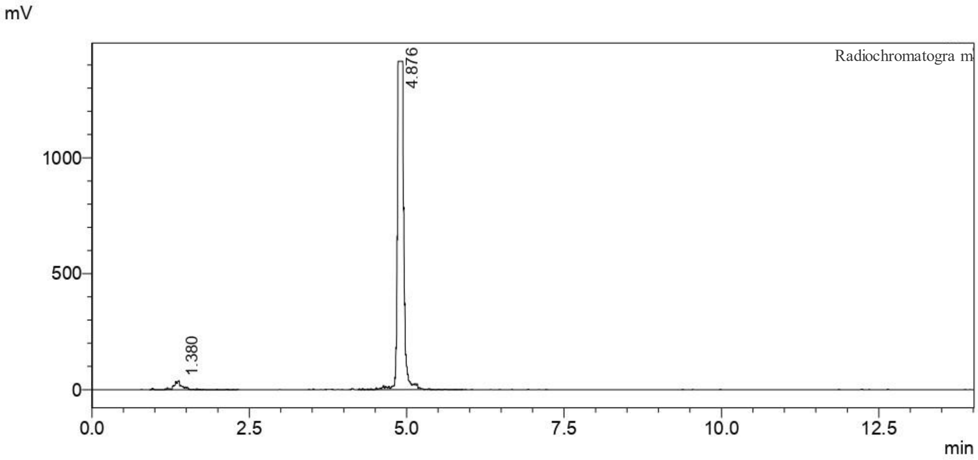


| Peak# | Ret. Time | Area | Area% |
|---|---|---|---|
| 1 | 1.38 | 325476 | 1.177 |
| 2 | 4.876 | 9917696 | 98.823 |
| Total | | 10243172 | 100 |

Radiochromatogram of [$^{68}$Ga]Ga-DOTA-**GA13**. Desired product obtained at >98% purity at retention time of 4.876 min. Minor radioactive peak (<2%) appearing at retention time 1.38 min represents unlabelled radioisotope at solvent front. Kinetex XB-C18 column (5 µm, 100 Å, 150 × 4.60 mm) eluted at 1.5 mL/min with a gradient of MeCN: 0.1% (w/v) formic acid, starting at 15% MeCN, increased to 50% B over 7 min and then increased to 90% and maintained at 90% MeCN for 3 min.

## Materials and Methods: Biology

### Circular Dichroism (CD)

Circular dichroism (CD) measurements were acquired on a Chirascan-plus spectropolarimeter (Applied photophysics, United Kingdom). The CD experiments were performed between 195 nm and 260 nm in triplicate with 1 nm step size, 1 nm bandwidth, 1 s time-per-point and 1 mm quartz cell (Starna, United Kingdom). Signal was recorded as millidegree. The DPC micelle background was subtracted, and the spectra were baseline corrected to 0 mdegree at 260 nm.

### *In Vitro* $CCK_2R$-Ligand Competitive Binding Assays

All $CCK_2R$ binding experiments were conducted using A431 (human epidermoid carcinoma) cells stably transfected to over-express the human full-length $CCK_2R$ (A431-$CCK_2R$) (gifted by Dr. Elisabeth von Guggenberg, Medizinische Universität Innsbruck).[41] As a negative control, A431 cells stably transfected with an empty vector were analysed simultaneously (A431-EV). All A431 cells were maintained in DMEM (Gibco, Australia) media supplemented with 10% foetal calf serum and 250 µg/mL G418 as described previously.[32]

The affinity of all peptides for the $CCK_2R$ was evaluated using competitive binding assays against [$^{177}$Lu]Lu-DOTA-**CP04** in A431-$CCK_2R$ cells. Briefly, 48 hours before the experiment cells were plated at a density of 300,000 cells per well in 12 well plates. On the day of the experiment, linear peptides were diluted in DMEM supplemented with 1% foetal calf serum to at least 7 different concentrations (0–400 nM) and approximately 500,000 cpm of [$^{177}$Lu]Lu-DOTA-**CP04** was added to each dilution. Cells were washed twice in ice-cold binding buffer (DMEM supplemented with 1% foetal calf serum) and resuspended in 1 mL of the pre-prepared peptide mix before being incubated at room temperature for 1 hour. Post-incubation the media was removed and kept for analysis (unbound fraction) and the cells were washed twice in ice-cold PBS which was pooled with the unbound fraction. Finally, 1 M NaOH (150µL) was added

to the wells and allowed to incubate for 5 min before lysates were collected (bound fraction). A small volume of lysate was kept aside to determine protein concentration using the Pierce® BCA protein assay kit (Thermo Fisher, Australia). All samples were analysed using the Perkin Elmer 2480 Wizard2™ gamma counter (PerkinElmer, Massachusetts, USA) and were normalised according to protein concentration. Half maximal inhibitory concentration ($IC_{50}$) was determined using nonlinear regression calculated using GraphPad Prism Software (GraphPad Software, California, USA).

**LogD$_{7.4}$ Determination**

To determine the distribution coefficient (logD$_{7.4}$), $^{68}$Ga radiolabeled peptides [$^{68}$Ga]Ga-DOTA-**GA4**, [$^{68}$Ga]Ga-DOTA-**GA7**, and [$^{68}$Ga]Ga-DOTA-**GA11**–**GA13** were diluted to approximately 20 pmol/mL in PBS (pH 7.4) and an equal volume of n-octanol was added. The mixture was vortexed vigorously for 10 cycles of 1 min at room temperature before being centrifuged for 6 min to separate the two phases. Equal volumes from each phase were aliquoted into individual counting tubes and analysed for 1 min using the Perkin Elmer 2480 Wizard2™ gamma counter (PerkinElmer, Massachusetts, USA). The distribution coefficient was calculated using the following equation LogD$_{oct/wat}$ = $log\left(\frac{[cpm]oct}{[cpm]PBS}\right)$.

**Protein Binding and Free Fraction Determination**

$^{68}$Ga radiolabeled peptides [$^{68}$Ga]Ga-DOTA-**GA4**, [$^{68}$Ga]Ga-DOTA-**GA7**, and [$^{68}$Ga]Ga-DOTA-**GA11**–**GA13** were assayed in duplicate at a final concentration between 700–800 pmol/mL in pooled human serum. As a control, peptides were incubated in 20 mM HEPES, pH 7.3. Mixtures were incubated for 1 hour at 37 °C before being loaded onto a Centrifree® Ultrafiltration spin column with a MW cut-off of 30 kDa (MilliporeSigma, Massachusetts, USA). Samples were centrifuged for 45 min at room temperature and radioactivity in the eluate

(free radiolabeled peptide) and retentate (protein-bound radiolabeled peptide) were measured in a gamma counter.

**Cell Internalisation Experiments**

To determine whether binding of radiolabeled peptides induced CCK-2 receptor internalisation, assays were performed using A431-CCK$_2$R or EV control cells. Forty-eight hours prior to the experiment, cells were seeded at a density of 300,000/well in a 12 well plate and grown at 37 °C. On the day of the experiment, cells were washed twice in ice-cold binding buffer (DMEM supplemented with 1% foetal calf serum) before being resuspended in either 1 mL binding buffer (specific binding) or 1 mL blocking solution (13.3 µM YM022 in binding buffer, non-specific binding). Cells were incubated at 37 °C for 15 min before the media was removed and replaced with binding buffer containing $^{68}$Ga radiolabeled peptides [$^{68}$Ga]Ga-DOTA-**GA4**, [$^{68}$Ga]Ga-DOTA-**GA7**, and [$^{68}$Ga]Ga-DOTA-**GA11**–**GA13** at a final concentration of 40 nM. Cells were incubated at 37 °C for 30, 60, 120 or 240 min. At each time point incubation was terminated by collecting the media and washing the cells twice with ice-cold binding buffer. Like affinity binding assays, media and washes were pooled and aliquoted for counting (total radioactivity added). To determine the membrane-bound fraction, cells were washed twice with 1 mL of 50 mM glycine (pH 2.8, 5 min incubation) and each wash was pooled for counting. Finally, the cells were lysed with 1 M NaOH and lysates collected to determine the internalised fraction. To calculate specific peptide internalisation, non-specific and membrane bound peptide counts were subtracted from the internalised fraction and this value was expressed as a percentage of the total radioactivity added to cells. Each time point was assayed in triplicate and all values are expressed as the mean ±SD.

**Metabolic Studies**

To assess the stability of $^{68}$Ga radiolabeled peptides against enzymatic degradation, duplicate samples (750-900 pmol/mL, 2-5MBq) were incubated at 37 °C with pooled human serum, 15%

mouse kidney homogenate, 30% mouse liver homogenate or 20 mM HEPES, pH 7.3 (control). Human blood was collected, and serum prepared using SSTII advance vacutainer tubes according to the manufacturer's instructions (Becton Dickinson, New Jersey, USA). Mouse kidney and liver homogenates were prepared by washing the tissues in ice-cold HEPES buffer, pH 7.3 and placing them in pre-chilled tubes containing HEPES buffer and 2.38 mm metal beads (Mo-Bio Laboratories, Hilden, Germany). Tissues were homogenised using the PowerLyzer24 homogeniser (Qiagen, Hilden, Germany) according to the manufacturer's instructions. $^{68}$Ga radiolabeled peptides were then incubated with the various lysates at 37 °C and samples were collected at 15, 30, 60 and 90 min. Reactions were terminated by addition of an equal volume of acetonitrile and centrifugation for 2 min at 15,000 rpm. Supernatants were diluted in water before being analysed by HPLC using a Kinetex C18 XB column (5µm 4.6x150mm), eluted at 1.5 mL/min using MeCN in water containing 0.1% formic acid over 7 min.

**Small Animal PET Imaging and Biodistribution Studies**

Female NSG mice (age 11-14 weeks) were sourced from Animal Resources Centre, (Canning Vale, Western Australia) and were inoculated subcutaneously on the right flank with 1-3 x $10^6$ A431-CCK$_2$R cells in PBS:Matrigel (1:1). Mice were weighed and tumours measured twice weekly using electronic callipers with tumour volume (mm$^3$) calculated as 0.5 × length × width. Mice were assigned to imaging and/or biodistribution groups (tumour volumes: 119-327 mm$^3$). The respective $^{68}$Ga labelled tracer (1.63–3.81 MBq, 170µL, 13.7–14.2 pmoles) containing vehicle solution (75µL, DMSO:Tween 80: water, 2:2:6 v/v/v) was then administered to mice intravenously *via* tail vein injection. Three mice were euthanized for biodistribution 1 hour post injection (n=3) and selected tissues were excised, weighed, and counted using a Capintec (Captus 4000e) gamma counter. Three mice were anaesthetised using 1.5% isoflurane and imaged at 1 hour (n=3) and 2 hours (n=3) with a G8 Small Animal PET/CT scanner (Perkin

Elmer/Sofie Biosciences). A 10 min static PET scan was acquired, followed immediately by a CT scan (Supplementary Data, pS51–S55). PET images were acquired using the G8 acquisition engine software and reconstructed using a 3D maximal likelihood and expectation maximization (ML-EM) algorithm. PET images were analysed using VivoQuant software, version 3.0 (inviCRO Imaging Services and Software) to quantify maximum standardised uptake value in regions of interest ($SUV_{max}$). Background regions for imaging experiments were calculated by quantitating mean standardised uptake value (SUVmean) of the abdomen and gut region. The region of interest was selected due to low uptake in abdomen/gut during imaging. After imaging the 2-hour time point, mice organs were harvested for biodistribution analysis as above.

For blocking $CCK_2R$ binding a solution of N-[(3R)-2,3-Dihydro-1-[2-(2-methylphenyl)-2-oxoethyl]-2-oxo-5-phenyl-1H-1,4-benzodiazepin-3-yl]-N′-(3-methylphenyl)-urea (YM022; Sigma Aldrich) was used. YM022 (75µL of a 1 mg/ml solution constituting DMSO:Tween 80: water, 2:2:6 v/v/v) was added to the respective $^{68}$Ga labelled tracer (1.41–3.61 MBq, 100 µL, 13.7–14.2 pmoles) and this mixture (total 170 µL) was administered in three mice intravenously *via* tail vein injection and organs harvested at 1 hour post injection for biodistribution analysis, as above.

All animal experiments were performed with the approval of the Peter MacCallum Cancer Centre Animal Experimentation Ethics Committee and in accordance with the Australian code for the care and use of animals for scientific purposes, 8th Edition, 2013 (AEEC Approval E654).

# Biological assays results

## Metabolism studies: [$^{68}$Ga]Ga-DOTA-GA4

**HEPES (control)**

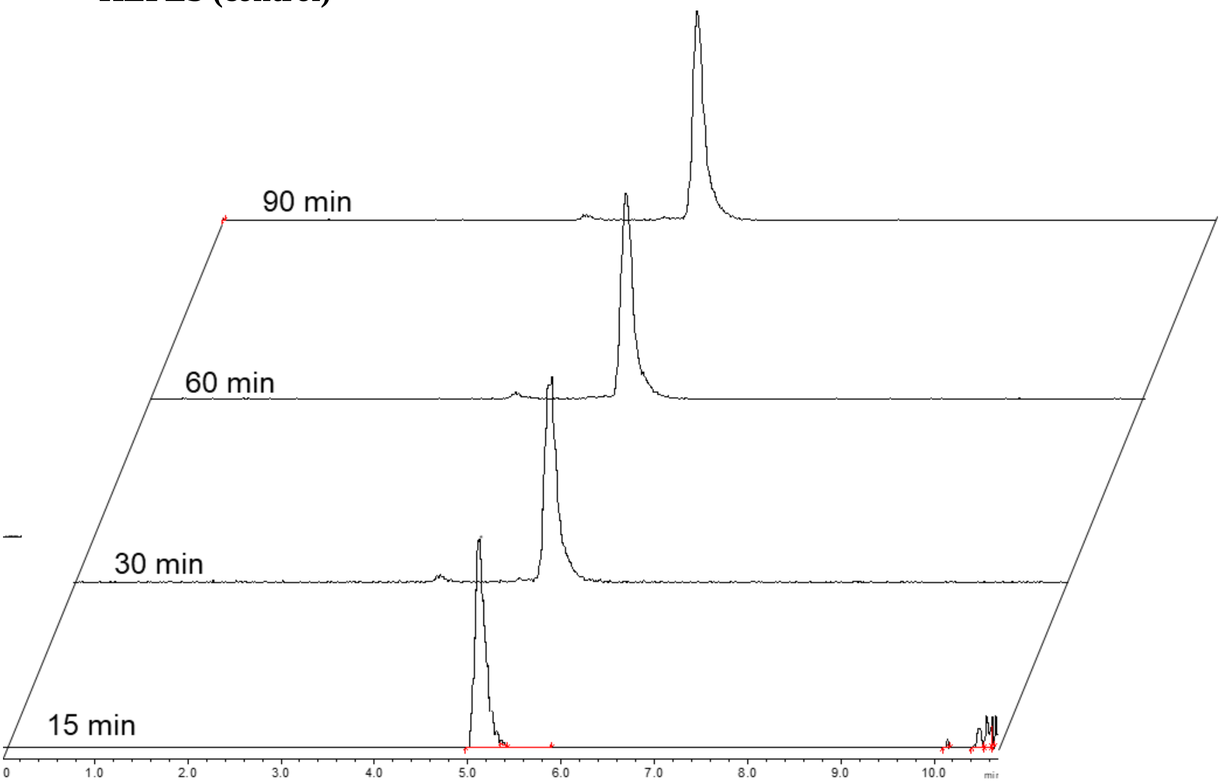

**Serum**

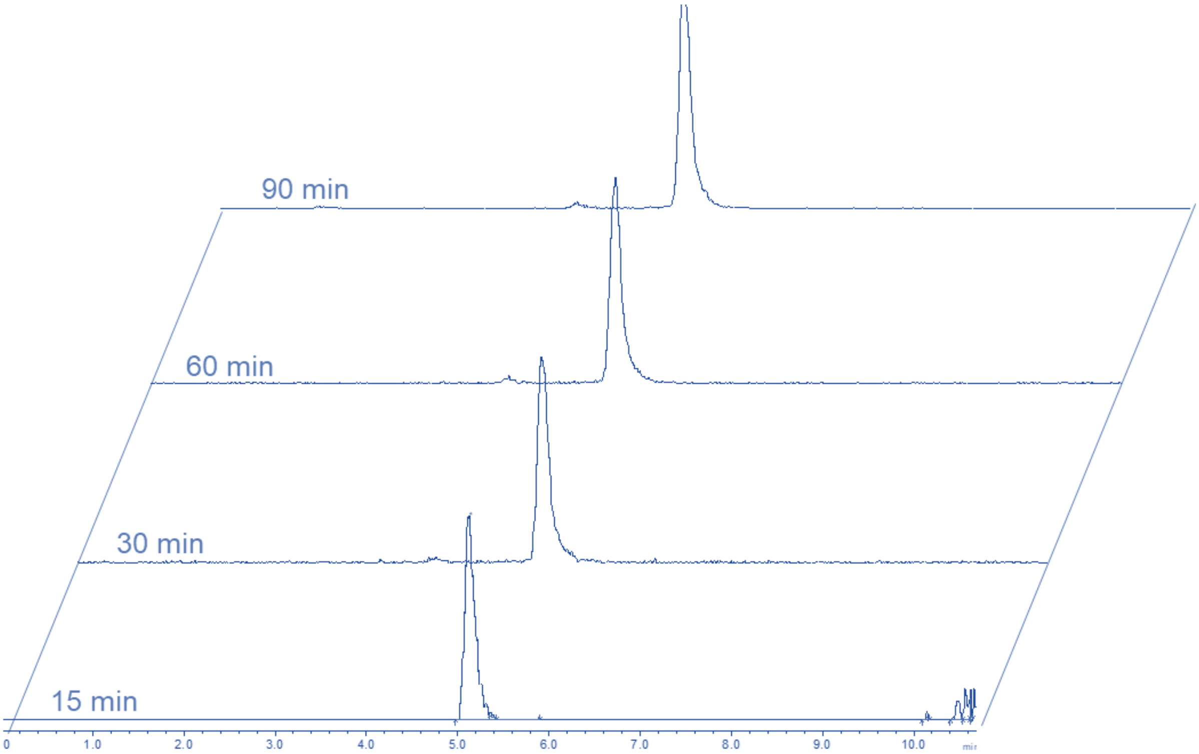


**Kidney**

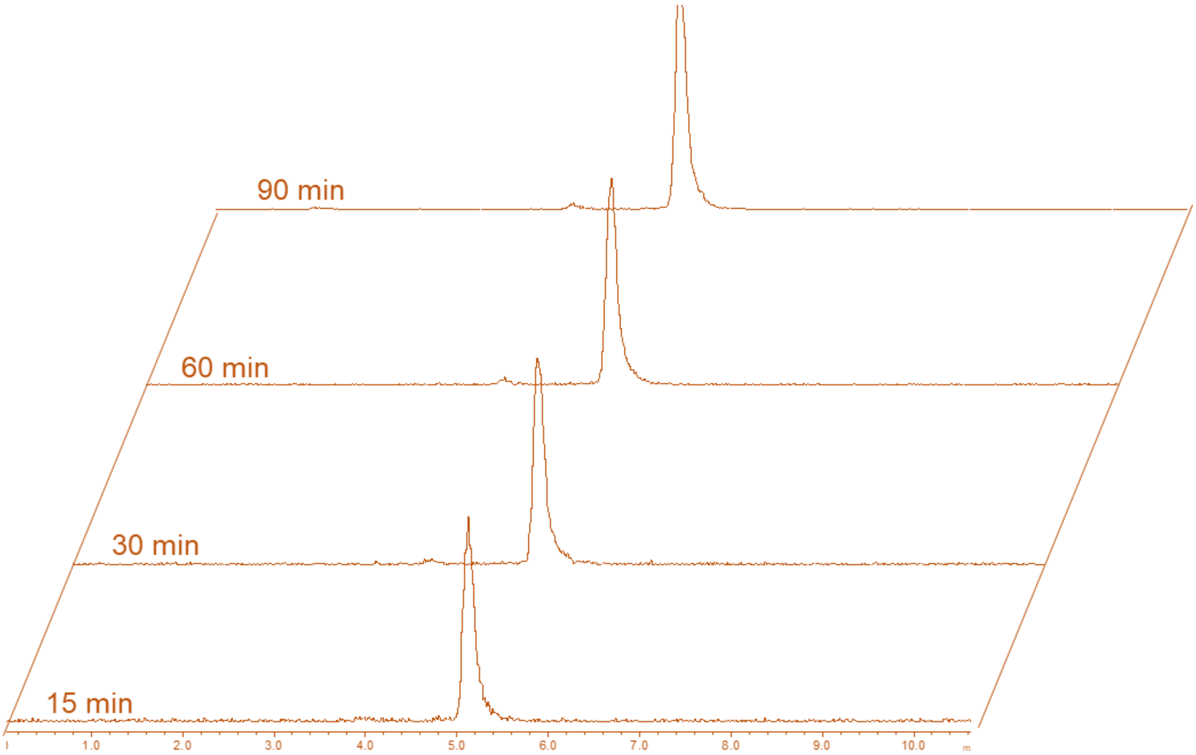


**Liver**

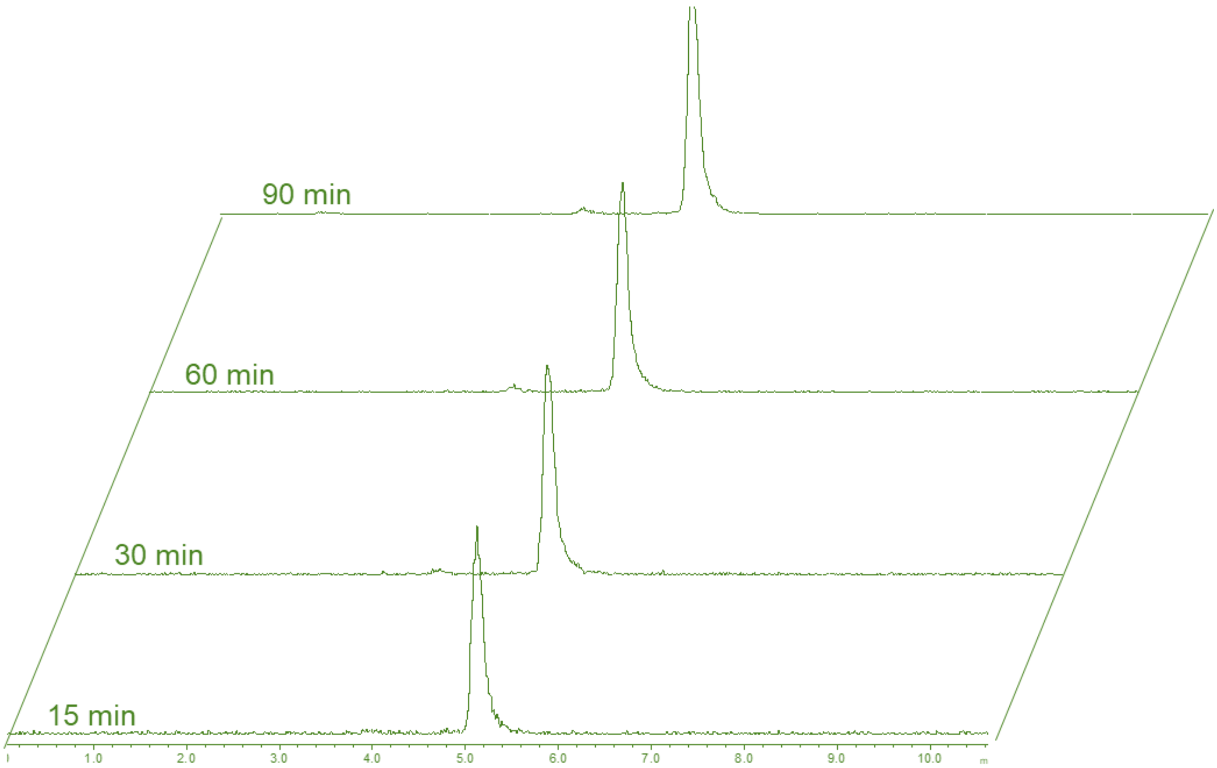
90 min
60 min
30 min
15 min
1.0
2.0
3.0
4.0
5.0
6.0
7.0
8.0
9.0
10.0

**Metabolism studies: [$^{68}$Ga]Ga-DOTA-GA7**

**HEPES (control)**

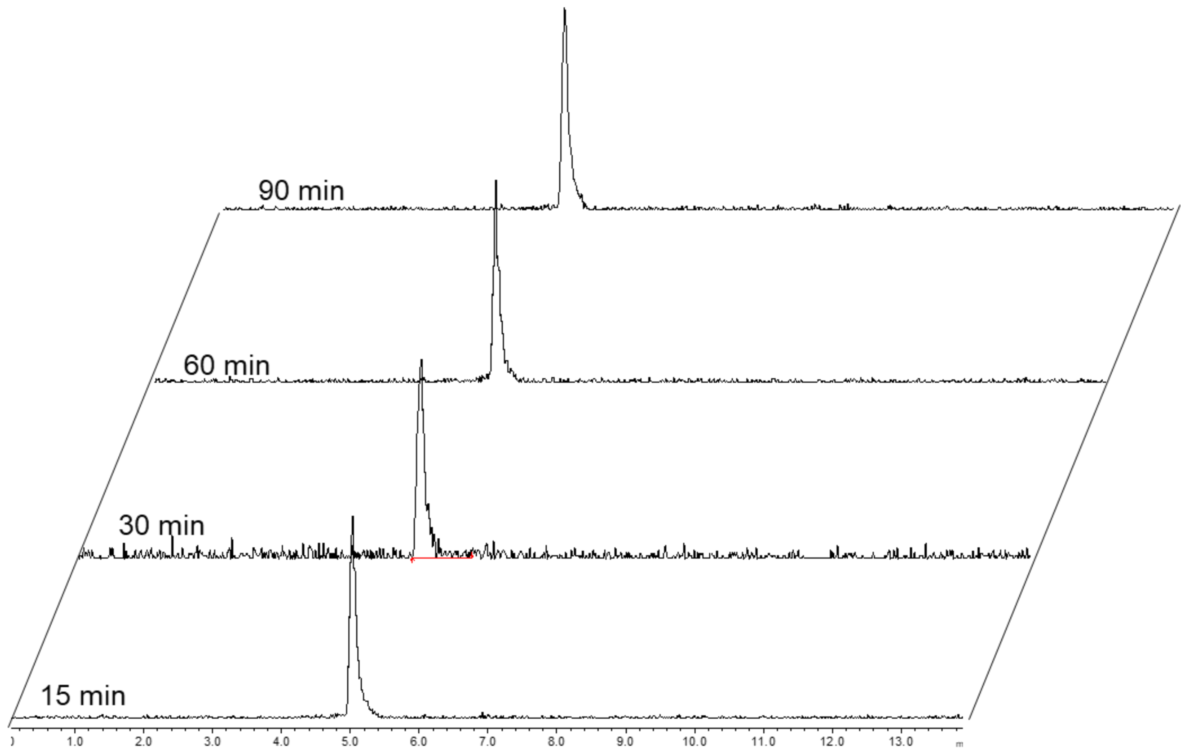


**Serum**

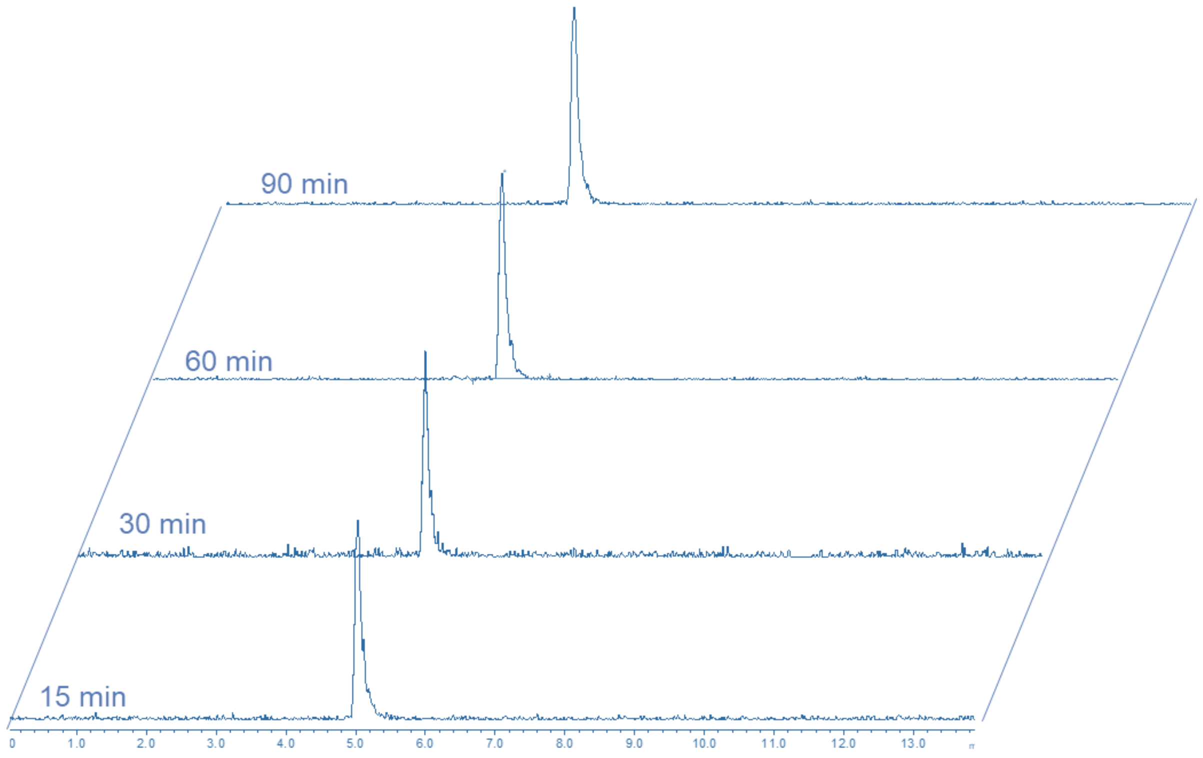

**Kidney**

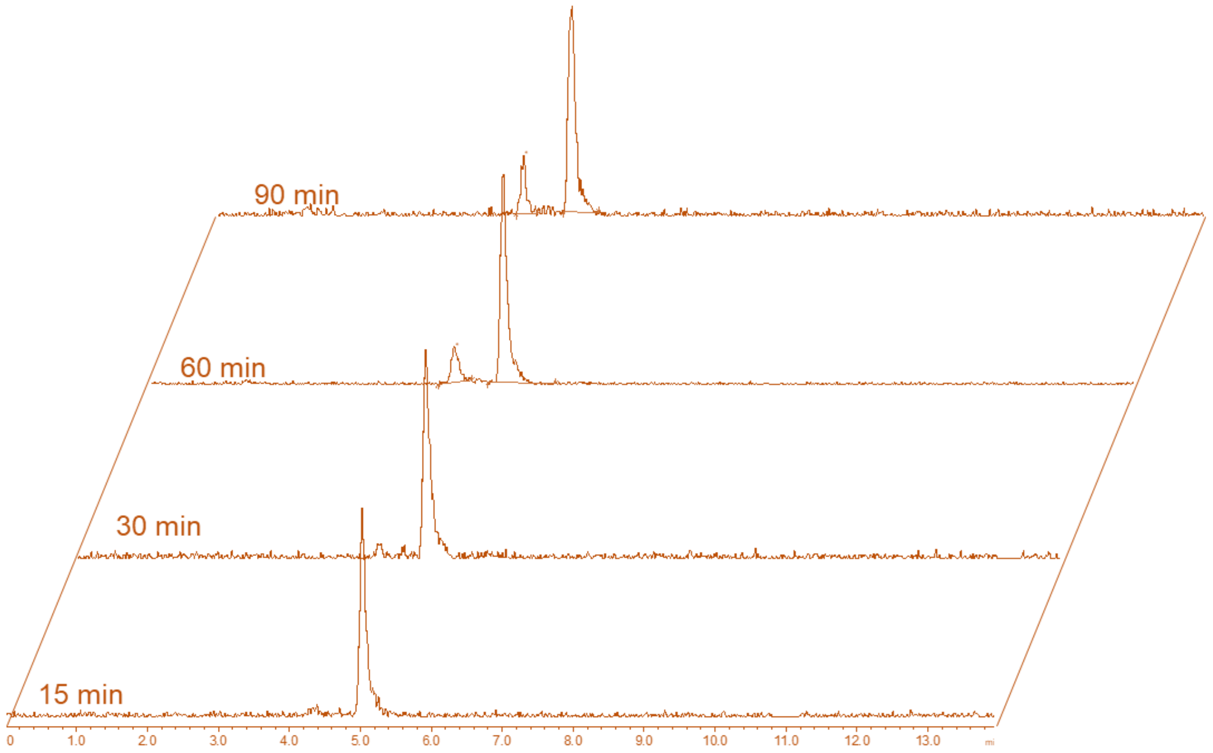


**Liver**

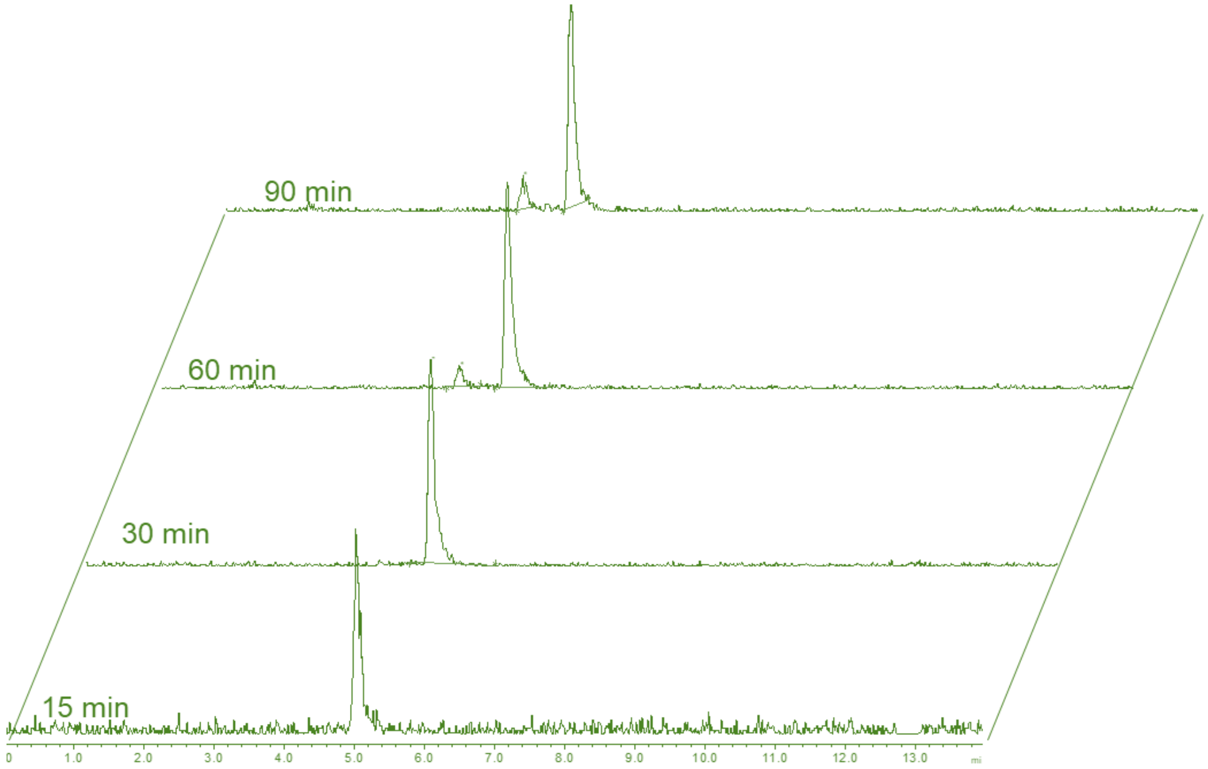

**Metabolism studies: [$^{68}$Ga]Ga-DOTA-GA11**

**HEPES (control)**

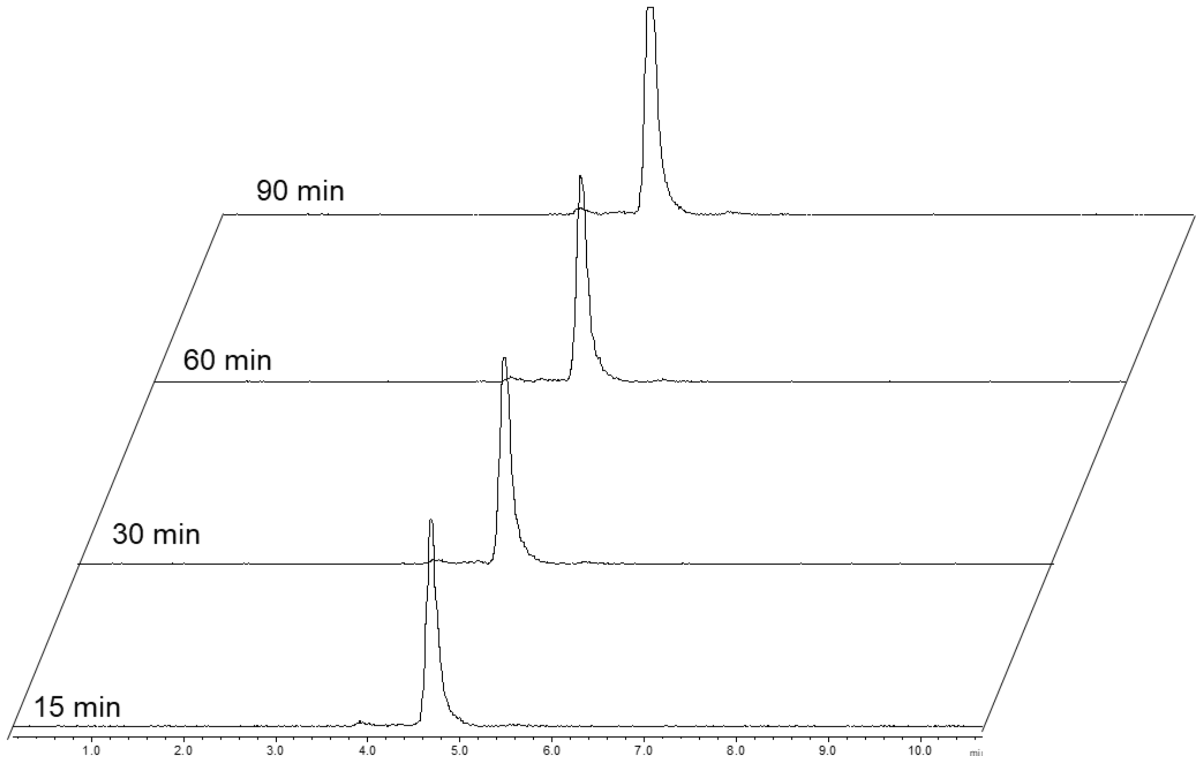


**Serum**

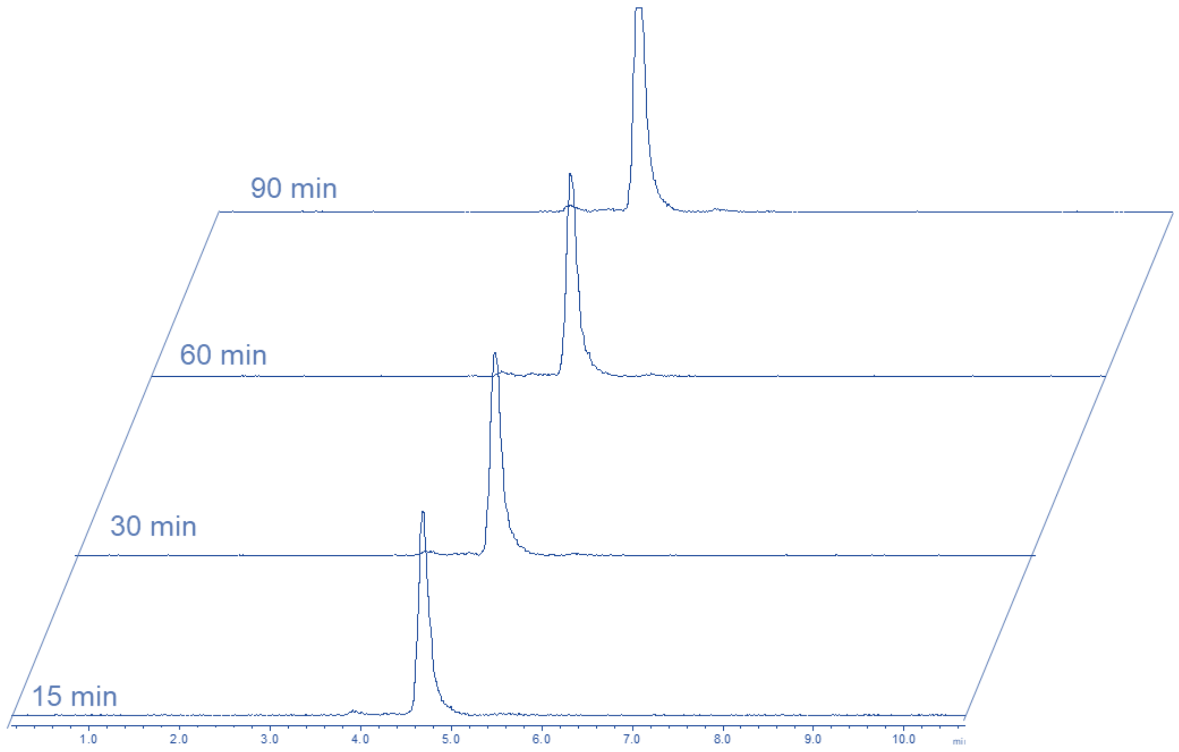

**Kidney**

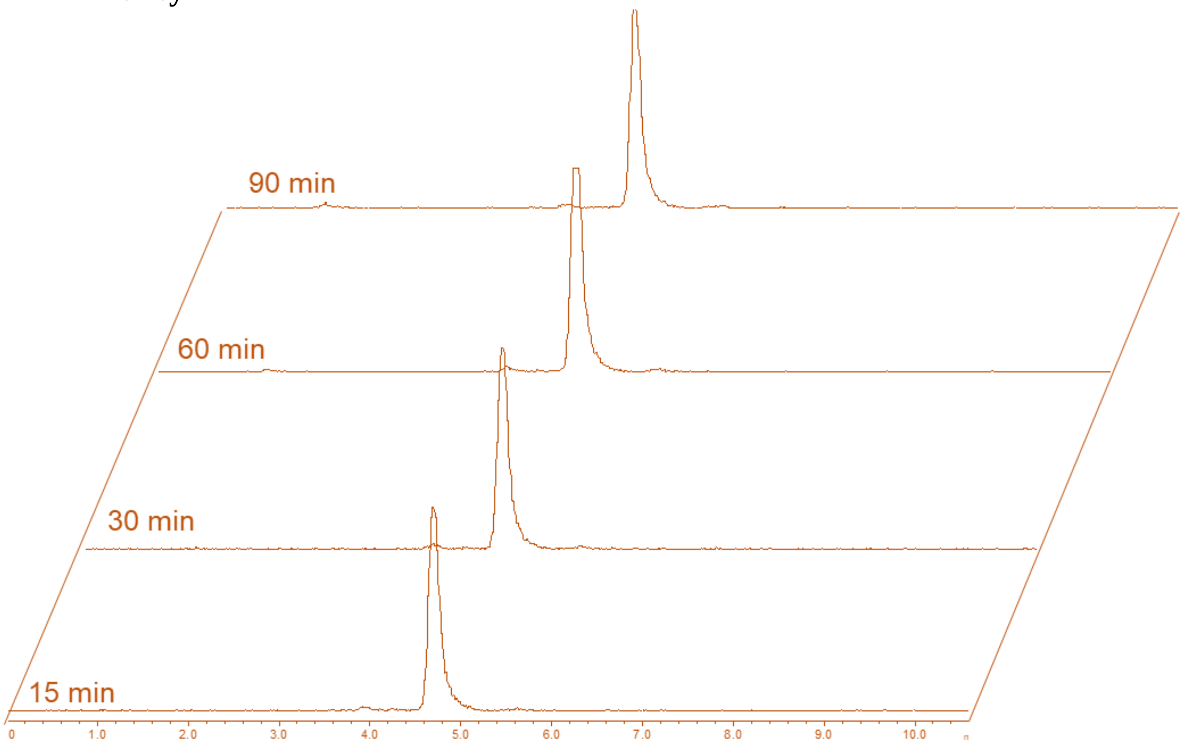


**Liver**

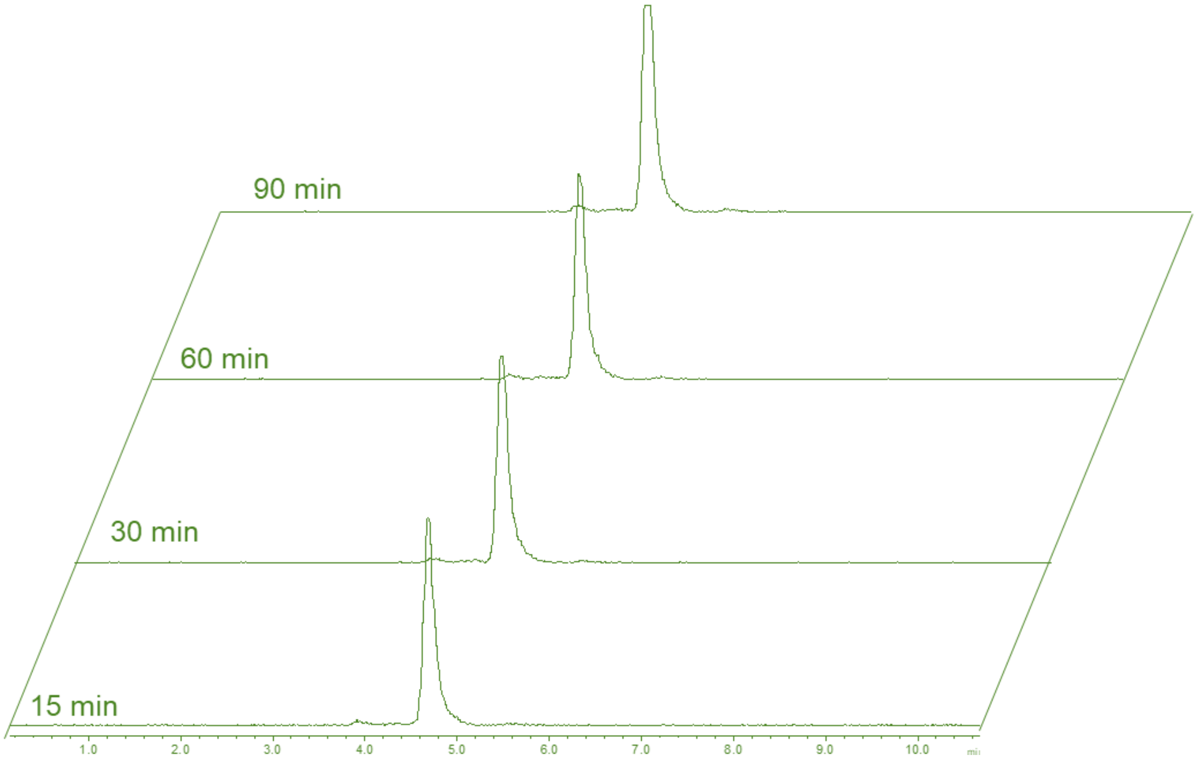

**Metabolism studies: [$^{68}$Ga]Ga-DOTA-GA12**

**HEPES (control)**

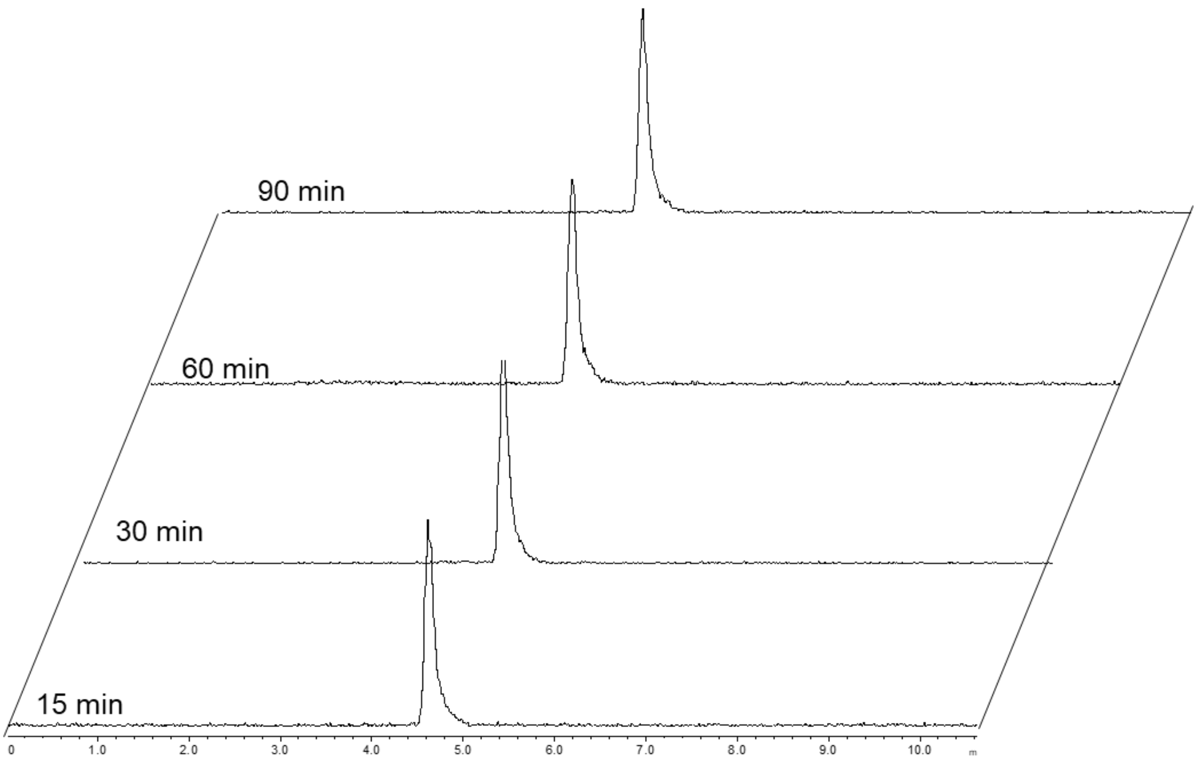


**Serum**

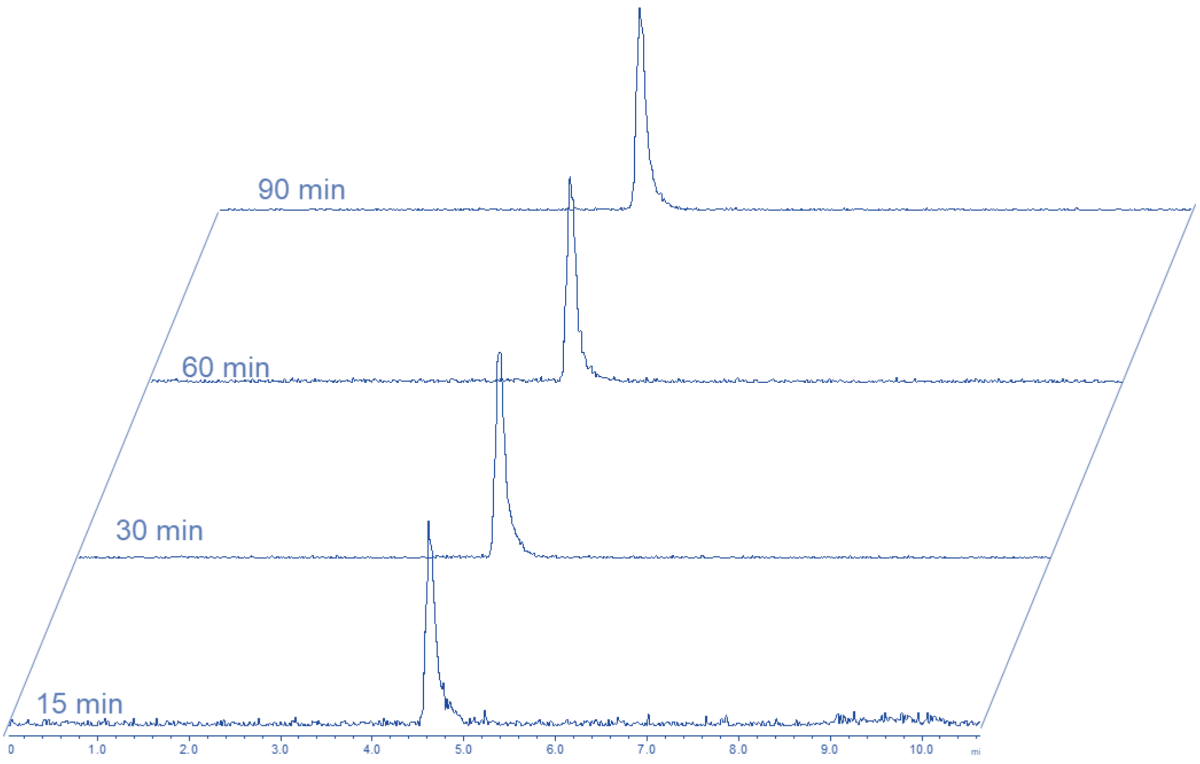

**Kidney**

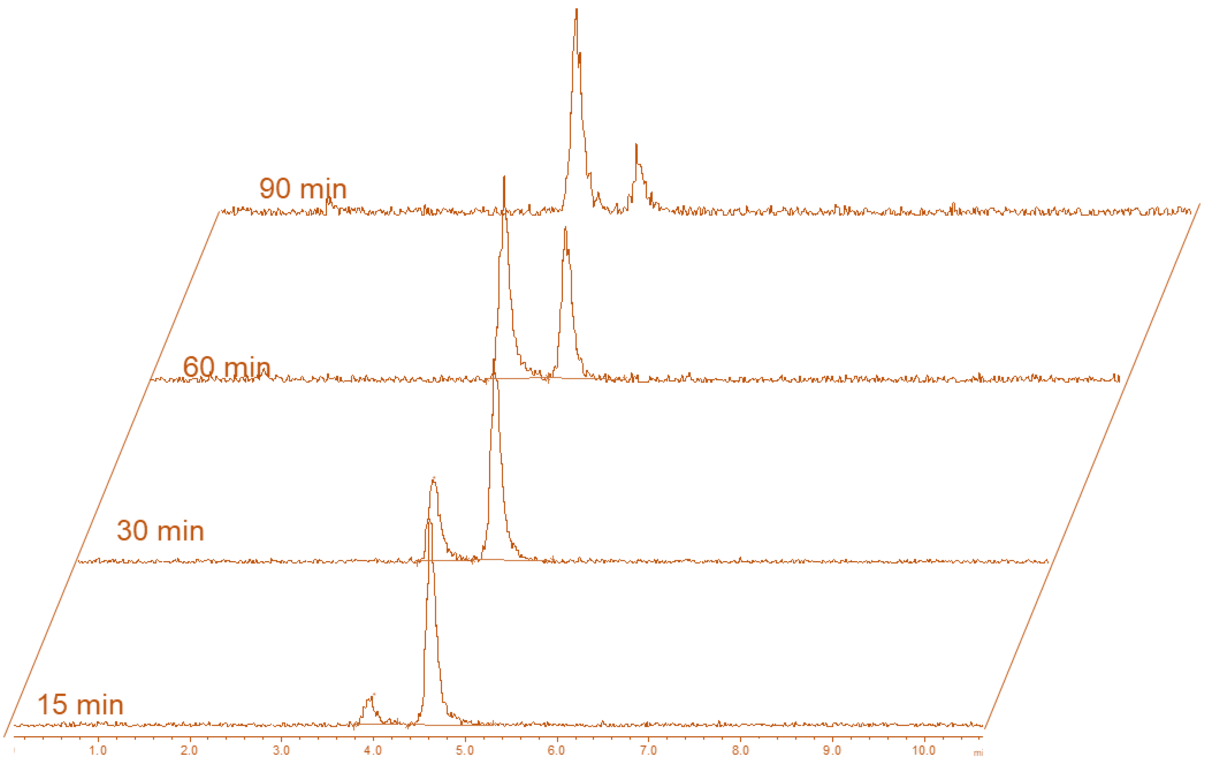


**Liver**

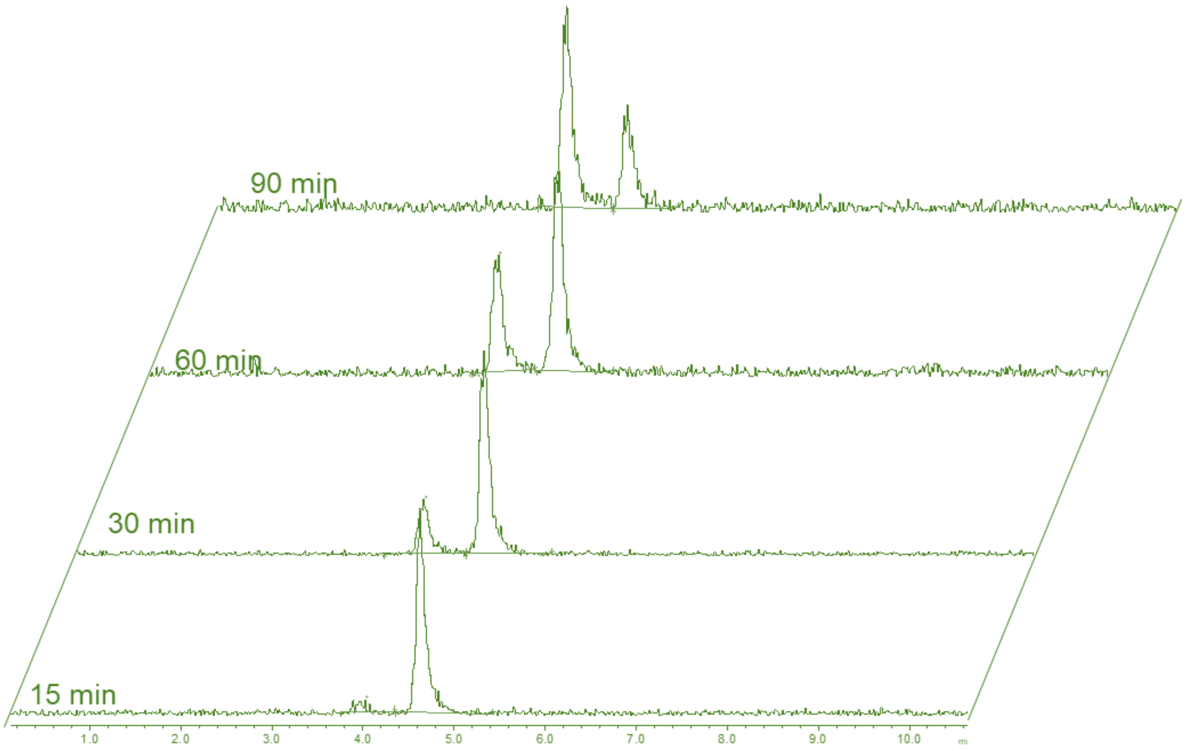

**Metabolism studies: [$^{68}$Ga]Ga-DOTA-GA13**

**HEPES (control)**

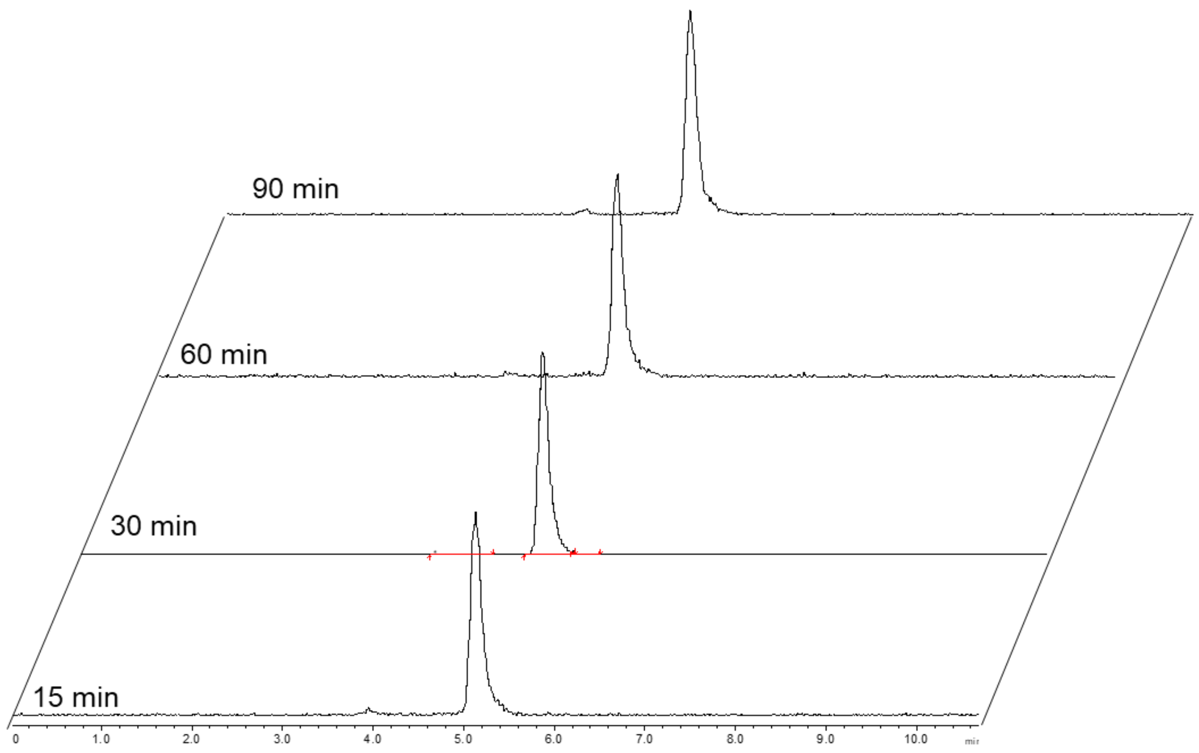


**Serum**

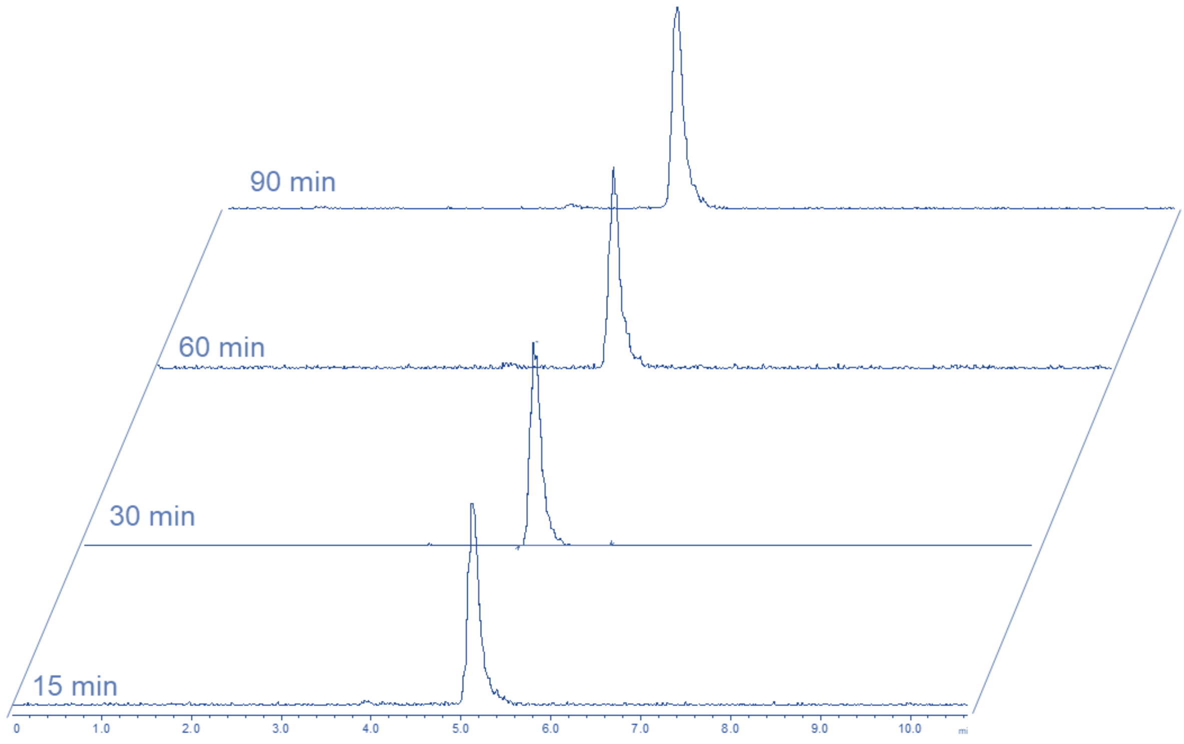

**Kidney**

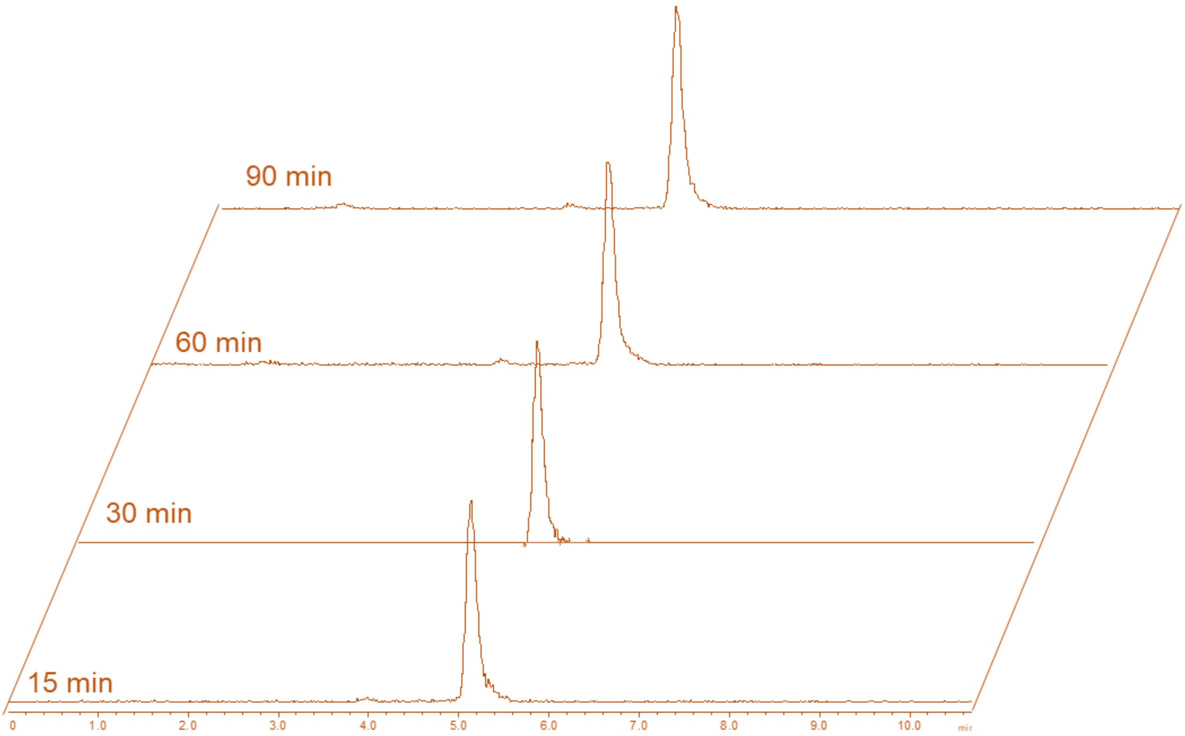


**Liver**

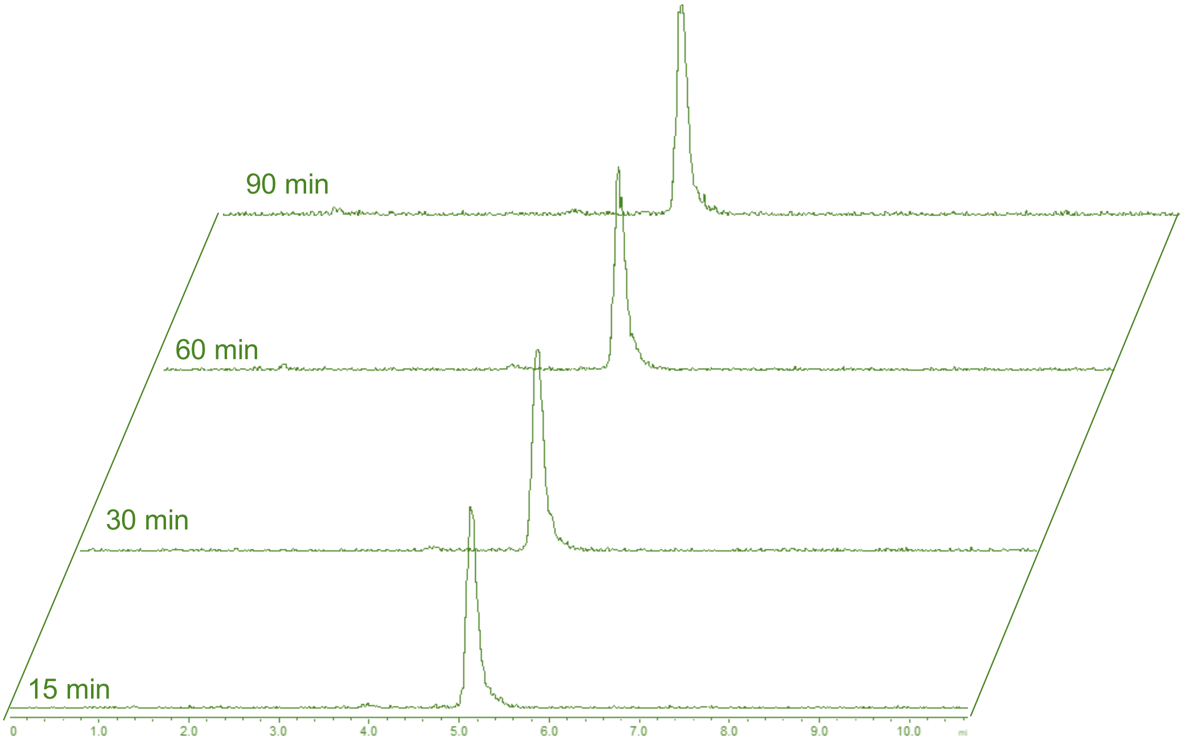

**Imaging SUV: [$^{68}$Ga]Ga-DOTA-GA4**

***Imaging scans, SUV$_{max}$ and Tumour:Organ Ratios at 1hr and 2hr***

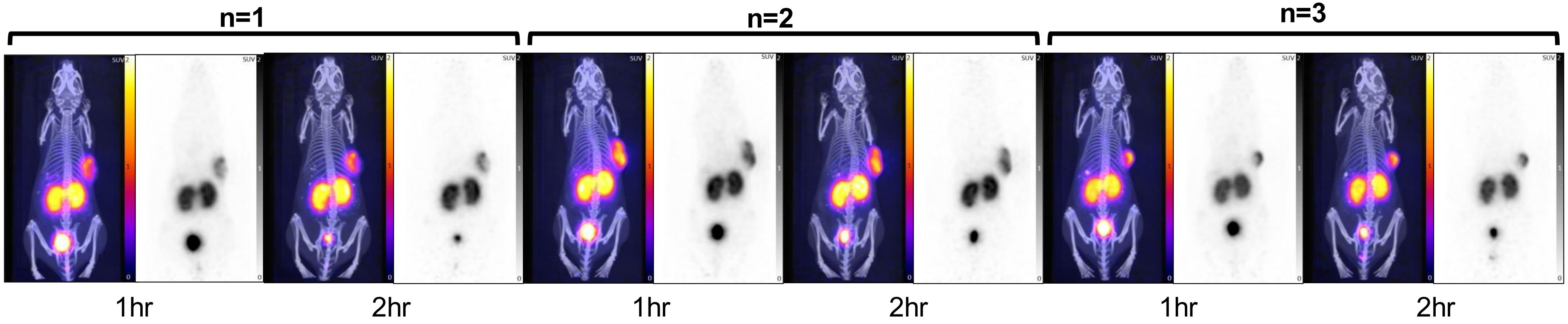


| **1 hr** | | | | | | | | | | |
|---|---|---|---|---|---|---|---|---|---|---|
| **Mouse** | SUV$_{max}$ | Tumour (T) | Background (Bkg) | T:Bkg | Liver (L) | T:L | Kidney (K) | T:K | Stomach (S) | T:S |
| **n=1** | 1.17 | 0.53 | 0.12 | 9.89 | 0.14 | 8.37 | 1.60 | 0.73 | 0.22 | 5.37 |
| **n=2** | 1.53 | 0.70 | 0.13 | 11.41 | 0.16 | 9.47 | 1.62 | 0.94 | 0.26 | 5.84 |
| **n=3** | 1.53 | 0.53 | 0.12 | 13.05 | 0.13 | 11.41 | 1.39 | 1.10 | 0.22 | 7.06 |
| **Blocking** | 0.49 | 0.28 | 0.18 | 2.81 | 0.17 | 2.98 | 1.76 | 0.28 | 0.18 | 2.69 |

| **2 hr** | | | | | | | | | | |
|---|---|---|---|---|---|---|---|---|---|---|
| **Mouse** | SUV$_{max}$ | Tumour (T) | Background (Bkg) | T:Bkg | Liver (L) | T:L | Kidney (K) | T:K | Stomach (S) | T:S |
| **n=1** | 1.30 | 0.49 | 0.05 | 28.25 | 0.09 | 14.58 | 1.54 | 0.84 | 0.17 | 7.66 |
| **n=2** | 1.49 | 0.64 | 0.06 | 25.37 | 0.10 | 14.55 | 1.62 | 0.92 | 0.25 | 5.99 |
| **n=3** | 1.46 | 0.50 | 0.05 | 27.85 | 0.08 | 18.01 | 1.42 | 1.03 | 0.22 | 6.54 |
| **Blocking** | 0.38 | 0.18 | 0.07 | 5.67 | 0.09 | 4.22 | 1.76 | 0.21 | 0.12 | 3.06 |

**Imaging SUV: [⁶⁸Ga]Ga-DOTA-GA7**

***Imaging scans, SUV$_{max}$ and Tumour:Organ Ratios at 1hr and 2hr***

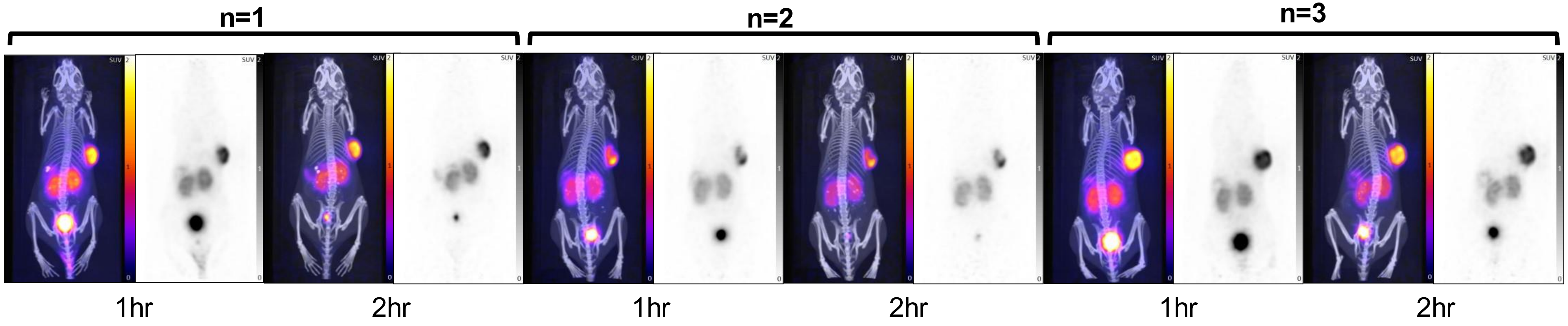


| **1 hr** | | | | | | | | | | |
|---|---|---|---|---|---|---|---|---|---|---|
| **Mouse** | SUV$_{max}$ | Tumour (T) | Background (Bkg) | T:Bkg | Liver (L) | T:L | Kidney (K) | T:K | Stomach (S) | T:S |
| **n=1** | 1.75 | 0.74 | 0.12 | 14.63 | 0.13 | 13.92 | 0.90 | 1.94 | 0.45 | 3.92 |
| **n=2** | 1.62 | 0.49 | 0.08 | 20.83 | 0.08 | 19.77 | 0.64 | 2.54 | 0.27 | 6.00 |
| **n=3** | 1.91 | 0.84 | 0.12 | 16.38 | 0.12 | 16.02 | 0.83 | 2.32 | 0.33 | 5.85 |
| **Blocking** | 0.65 | 0.27 | 0.09 | 7.09 | 0.09 | 7.10 | 0.61 | 1.07 | 0.16 | 4.19 |

| **2 hr** | | | | | | | | | | |
|---|---|---|---|---|---|---|---|---|---|---|
| **Mouse** | SUV$_{max}$ | Tumour (T) | Background (Bkg) | T:Bkg | Liver (L) | T:L | Kidney (K) | T:K | Stomach (S) | T:S |
| **n=1** | 1.89 | 0.70 | 0.04 | 53.06 | 0.07 | 25.46 | 0.78 | 2.44 | 0.30 | 6.42 |
| **n=2** | 1.62 | 0.46 | 0.04 | 41.86 | 0.06 | 28.34 | 0.60 | 2.70 | 0.24 | 6.85 |
| **n=3** | 1.82 | 0.77 | 0.04 | 44.31 | 0.07 | 24.37 | 0.71 | 2.58 | 0.27 | 6.71 |
| **Blocking** | 0.61 | 0.21 | 0.04 | 16.65 | 0.05 | 11.96 | 0.52 | 1.17 | 0.11 | 5.66 |

**Imaging SUV: [$^{68}$Ga]Ga-DOTA-GA11**

***Imaging scans, SUV$_{max}$ and Tumour:Organ Ratios at 1hr and 2hr***

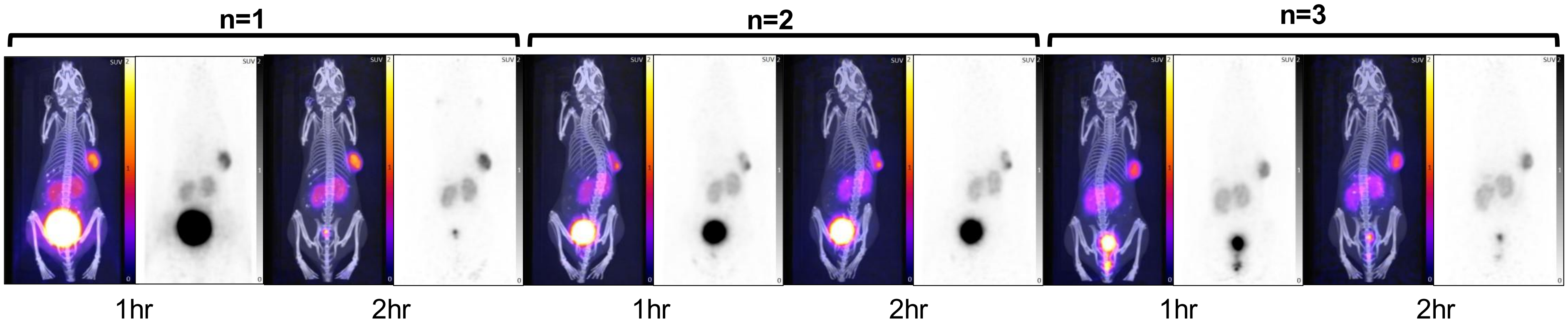


| 1 hr | | | | | | | | | | |
|---|---|---|---|---|---|---|---|---|---|---|
| **Mouse** | SUV$_{max}$ | Tumour (T) | Background (Bkg) | T:Bkg | Liver (L) | T:L | Kidney (K) | T:K | Stomach (S) | T:S |
| **n=1** | 1.29 | 0.58 | 0.13 | 10.30 | 0.14 | 9.09 | 0.67 | 1.92 | 0.21 | 6.23 |
| **n=2** | 1.12 | 0.34 | 0.06 | 18.05 | 0.07 | 15.76 | 0.51 | 2.17 | 0.13 | 8.79 |
| **n=3** | 0.95 | 0.40 | 0.06 | 14.78 | 0.07 | 13.62 | 0.50 | 1.88 | 0.17 | 5.55 |
| **Blocking** | 0.39 | 0.16 | 0.07 | 5.94 | 0.07 | 5.50 | 0.48 | 0.81 | 0.12 | 3.27 |

| 2 hr | | | | | | | | | | |
|---|---|---|---|---|---|---|---|---|---|---|
| **Mouse** | SUV$_{max}$ | Tumour (T) | Background (Bkg) | T:Bkg | Liver (L) | T:L | Kidney (K) | T:K | Stomach (S) | T:S |
| **n=1** | 1.35 | 0.53 | 0.04 | 31.08 | 0.06 | 21.07 | 0.59 | 2.28 | 0.15 | 8.90 |
| **n=2** | 1.19 | 0.33 | 0.03 | 39.34 | 0.05 | 25.00 | 0.46 | 2.60 | 0.15 | 7.71 |
| **n=3** | 0.97 | 0.38 | 0.04 | 26.89 | 0.05 | 17.82 | 0.43 | 2.25 | 0.17 | 5.60 |
| **Blocking** | 0.34 | 0.12 | 0.03 | 12.94 | 0.05 | 7.33 | 0.43 | 0.79 | 0.05 | 6.49 |

**Imaging SUV: [$^{68}$Ga]Ga-DOTA-GA12**

***Imaging scans, SUV$_{max}$ and Tumour:Organ Ratios at 1hr and 2hr***

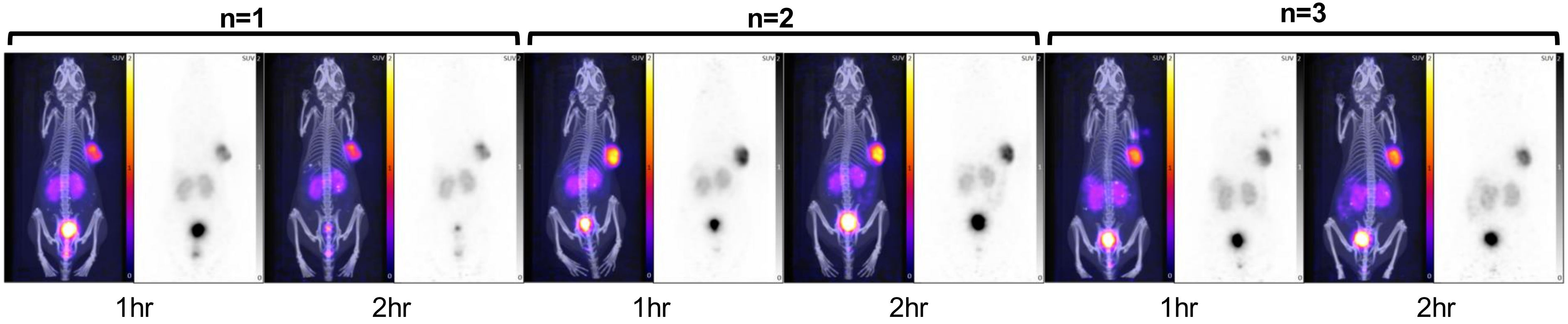


| **1 hr** | | | | | | | | | | |
|---|---|---|---|---|---|---|---|---|---|---|
| **Mouse** | SUV$_{max}$ | Tumour (T) | Background (Bkg) | T:Bkg | Liver (L) | T:L | Kidney (K) | T:K | Stomach (S) | T:S |
| **n=1** | 1.13 | 0.48 | 0.06 | 18.87 | 0.06 | 19.43 | 0.46 | 2.47 | 0.17 | 6.59 |
| **n=2** | 1.73 | 0.71 | 0.05 | 33.95 | 0.06 | 28.97 | 0.45 | 3.81 | 0.18 | 9.63 |
| **n=3** | 1.24 | 0.57 | 0.06 | 21.99 | 0.05 | 23.04 | 0.47 | 2.66 | 0.32 | 3.92 |
| **Blocking** | 0.50 | 0.24 | 0.05 | 9.78 | 0.05 | 9.18 | 0.35 | 1.45 | 0.05 | 10.24 |

| **2 hr** | | | | | | | | | | |
|---|---|---|---|---|---|---|---|---|---|---|
| **Mouse** | SUV$_{max}$ | Tumour (T) | Background (Bkg) | T:Bkg | Liver (L) | T:L | Kidney (K) | T:K | Stomach (S) | T:S |
| **n=1** | 1.15 | 0.44 | 0.02 | 46.17 | 0.04 | 30.91 | 0.41 | 2.79 | 0.14 | 8.18 |
| **n=2** | 1.66 | 0.68 | 0.03 | 63.43 | 0.05 | 35.45 | 0.41 | 4.07 | 0.22 | 7.57 |
| **n=3** | 1.37 | 0.54 | 0.03 | 52.84 | 0.04 | 34.79 | 0.43 | 3.21 | 0.21 | 6.48 |
| **Blocking** | 0.45 | 0.18 | 0.02 | 21.93 | 0.03 | 13.58 | 0.32 | 1.41 | 0.03 | 13.00 |

**Imaging SUV: [$^{68}$Ga]Ga-DOTA-GA13**

***Imaging scans, SUV$_{max}$ and Tumour:Organ Ratios at 1hr and 2hr***

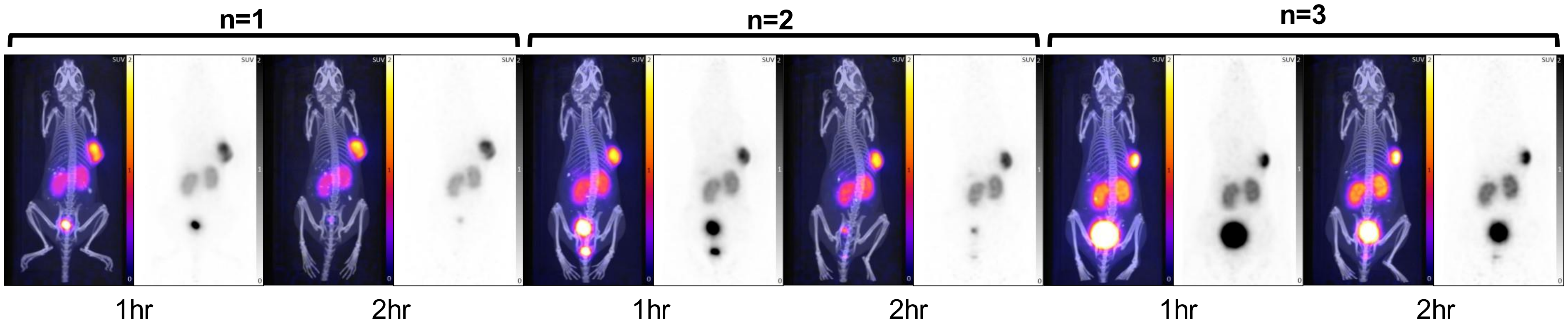


| 1 hr | | | | | | | | | | |
|---|---|---|---|---|---|---|---|---|---|---|
| **Mouse** | SUV$_{max}$ | Tumour (T) | Background (Bkg) | T:Bkg | Liver (L) | T:L | Kidney (K) | T:K | Stomach (S) | T:S |
| **n=1** | 1.63 | 0.71 | 0.07 | 22.27 | 0.10 | 17.01 | 0.62 | 2.61 | 0.18 | 9.08 |
| **n=2** | 1.76 | 0.74 | 0.10 | 17.74 | 0.11 | 15.69 | 0.84 | 2.09 | 0.23 | 7.62 |
| **n=3** | 2.18 | 0.86 | 0.12 | 18.05 | 0.12 | 17.88 | 1.07 | 2.04 | 0.31 | 7.15 |
| **Blocking** | 0.32 | 0.20 | 0.07 | 4.49 | 0.08 | 3.97 | 0.70 | 0.46 | 0.10 | 3.34 |

| 2 hr | | | | | | | | | | |
|---|---|---|---|---|---|---|---|---|---|---|
| **Mouse** | SUV$_{max}$ | Tumour (T) | Background (Bkg) | T:Bkg | Liver (L) | T:L | Kidney (K) | T:K | Stomach (S) | T:S |
| **n=1** | 1.64 | 0.68 | 0.03 | 49.00 | 0.07 | 24.70 | 0.62 | 2.66 | 0.07 | 23.36 |
| **n=2** | 1.66 | 0.70 | 0.03 | 49.96 | 0.07 | 25.52 | 0.80 | 2.07 | 0.15 | 10.82 |
| **n=3** | 2.12 | 0.81 | 0.05 | 45.70 | 0.07 | 30.34 | 1.03 | 2.06 | 0.22 | 9.50 |
| **Blocking** | 0.32 | 0.14 | 0.03 | 9.30 | 0.06 | 5.76 | 0.67 | 0.48 | 0.07 | 4.35 |
